# Genome‐wide identification of neuropeptides and their receptor genes in *Bemisia tabaci* and their transcript accumulation change in response to temperature stresses

**DOI:** 10.1111/1744-7917.12751

**Published:** 2020-05-25

**Authors:** Jiang‐Jie Li, Yan Shi, Gan‐Lin Lin, Chun‐Hong Yang, Tong‐Xian Liu

**Affiliations:** ^1^ Key Lab of Integrated Crop Pest Management of Shandong Province, College of Plant Health and Medicine Qingdao Agricultural University Qingdao Shandong China

**Keywords:** *Bemisia tabaci*, expression profiling, G‐protein‐coupled receptors, neuropeptide

## Abstract

Insect neuropeptides play an important role in regulating physiological functions such as growth, development, behavior and reproduction. We identified temperature‐sensitive neuropeptides and receptor genes of the cotton whitefly, *Bemisia tabaci*. We identified 38 neuropeptide precursor genes and 35 neuropeptide receptors and constructed a phylogenetic tree using additional data from other insects. As temperature adaptability enables *B. tabaci* to colonize a diversity of habitats, we performed quantitative polymerase chain reaction with two temperature stresses (low = 4 °C and high = 40 °C) to screen for temperature‐sensitive neuropeptides. We found many neuropeptides and receptors that may be involved in the temperature adaptability of *B. tabaci*. This study is the first to identify *B. tabaci* neuropeptides and their receptors, and it will help to reveal the roles of neuropeptides in temperature adaptation of *B. tabaci*.

## Introduction

*Bemisia tabaci* (Gennadius, Hemiptera: Aleyrodidae) is distributed in tropical, subtropical and some temperate regions of the world. It is an insect pest (Oliveira *et al*., 2001; González‐Zamora & Moreno, 2010; De Barro *et al*., 2011; Valle *et al*., 2012) with a wide host plant range. *B. tabaci* damages crops by direct feeding and by transmitting viral diseases. It often causes serious agricultural losses (Inbar & Gerling, 2008). It has developed resistance to many insecticides as a result of excessive insecticide use (Berg *et al*., 2007; Ahmad *et al*., 2010; Liang *et al*., 2012; Zheng *et al*., 2012). Therefore, novel *B. tabaci* management tools and strategies are needed.

More than 50 insect neuropeptide families have been identified. Identification and characterization of neuroendocrine‐related genes in genomes and transcriptomes have been proposed as the initial step in a “genome‐to‐lead” strategy for new insecticide discovery (Meyer *et al*., 2012). In insects, neuropeptides help regulate development, reproduction, feeding, courtship, olfaction, and circadian rhythms (Doerks *et al*., 2002; Nässel & Winther, 2010). Neuropeptides are important signaling molecules in insects and most activate G‐protein‐coupled receptors (GPCRs) to regulate physiological functions. These affect the signal transduction of neuropeptide signaling systems. For example, prothoracicotropic hormone (PTTH) in *Drosophila melanogaster* stimulates the secretion of ecdysone to regulate molting (Ghosh *et al*., 2010). Molting in *Rhodnius prolixus* is regulated by orcokinin (Wulff *et al*., 2017). The neuropeptide F (NPF) is involved in the regulation of feeding behavior in *Acyrthosiphon pisum* (Li *et al*., 2018). The CCHamide 1 receptor modulates sensory perception and olfactory behavior in starved *Drosophila* (Farhan *et al*., 2013). The SIF amide modulates sexual behavior in *Drosophila* (Terhzaz *et al*., 2007). Neuropeptide signaling molecules in insects regulate neuronal synthesis and secretion involved in physiological processes and behavior. They would satisfy the requirement of having a novel mode of action (Verlinden, 2014). Several studies have suggested the potential role of Central Nervous System (CNS) in temperature tolerance (Yoder *et al*., 2006). For example, the CAPA neuropeptide gene and its encoded peptides alter cold tolerance. The CAPA peptide signaling regulation of cellular ions and water in Malpighian tubules of *Drosophila* is a key physiological mechanism for recovery from cold stress (Terhzaz *et al*., 2015; Andersen *et al*., 2017; MacMillan *et al*., 2018). DH31‐PDFR signaling specifically regulates a preferred temperature decrease at night‐onset (Goda *et al*., 2016). However, there is still lack of research on the mechanism of neuropeptide regulation of *B. tabaci*.

In this study, we identified neuropeptide precursor genes and their receptors by analyzing the published genome and transcriptome of *B. tabaci* MEAM1 (Xie *et al*., 2017). We also identified neuropeptide precursors and receptors in the *B. tabaci* genome by comparison with five other insect species with well‐characterized neuropeptidomes. Using a liquid chromatography tandem mass spectrometry (LC‐MS/MS) approach, we confirmed the presence of mature neuropeptides encoded by some of these precursors in *B. tabaci*. We analyzed the expression profiles of neuropeptides and receptor genes in *B. tabaci* under different temperatures.

## Materials and methods

### Insect rearing and temperature treatments

*B. tabaci* were collected from cotton plants in a greenhouse in Jinan, Shandong Province, China in 2012. The laboratory population of *B. tabaci* MEAM1 was reared inside a cage (400 mm × 500 mm × 450 mm) in a greenhouse maintained at 27 °C ± 0.5 °C and a 16 : 8 (L : D) photoperiod. In the temperature treatments, *B. tabaci* were separately reared on tomato seedlings at 4 °C, 27 °C and 40 °C for 1 h and 4 h, respectively.

### Identification of the neuropeptides and their putative GPCRs in B. tabaci

Based on data from *Acyrthosiphon pisum* (Huybrechts *et al*., 2010), *R. prolixus* (Ons, 2017), *Tribolium castaneum* (Amare & Sweedler, 2007), *D. melanogaster* (Nässel & Winther, 2010), and *Zootermopsis nevadensis* (Veenstra, 2014), the neuropeptides of *B. tabaci* were analyzed. The neuropeptide precursors included the predicted signal peptides (http://www.cbs.dtu.dk/services/SignalP/) and the prediction of mature peptides. The predicted neuropeptide receptors of *B. tabaci* were based on the predicted receptor genes of *Diaphorina citri* (Wang *et al*., 2018), *Nilaparvata lugens* (Tanaka *et al*., 2014), *Bombyx mori* (Fan *et al*., 2010), *T. castaneum* (Hauser *et al*., 2008), *D. melanogaster* (Nässel & Winther, 2010; Audsley *et al*., 2015), and *Z. nevadensis* (Veenstra, 2014). The candidate neuropeptides and receptor genes were identified by tBLASTn analysis (Altschul *et al*., 1990; Tanaka *et al*., 2014; Ons *et al*., 2016) with the non‐redundant protein sequence (NR) at NCBI (http://www.ncbi.nlm.nih.gov/) and the Whitefly Genome Database (http://www.whiteflygenomics.org/cgi-bin/bta/index.cgi). Upon comparing the sequences of the genome alignment, we selected the high hit scaffold sequence and used the Softberry website (http://linux1.softberry.com/) to predict the protein sequence of target neuropeptide genes.

### Phylogenetic analysis

Phylogenetic trees of *B. tabaci* receptors were constructed with *B. mori*, *D. melanogaster*, *D. citri*, *N. lugens*, *T. castaneum* and *Z. nevadensis* data. The sequence name used is the same name as used in the literature and the amino acid sequences are shown in Supplementary data S1. We used the TMHMM Server, v.2.05 (http://www.cbs.dtu.dk/services/TMHMM//) website to predict the transmembrane regions and then deleted the non‐transmembrane regions before aligning the different sequences. Then, all processed sequences were aligned using ClustalX2 software with default settings. A neighbor‐joining tree was constructed in MEGA 5.2 with 1000 bootstrap replicates (Thompson *et al*., 1994; Kumar *et al*., 2008). The model chooses P‐distance and the Gaps/Missing DATA Treatment chooses Pairwise deletion. The data were converted into a figure by Evolview (http://www.evolgenius.info/evolview/#login).

### Identification of neuropeptides

*B. tabaci* adults were taken from the laboratory population and transferred to a grinding tube with a protein lysing solution (8 mol/L urea containing a protease inhibitor cocktail). The whiteflies were ground using a high throughput tissue grinder (Bullet Blender Blue, Troy, NY, USA) and triturated three times for 40 s each. The mixture was left on ice for 30 min, centrifuged once to obtain the supernatant, and then centrifuged a second time for ultrafiltration. The collected flow‐through solution was desalted using a Waters Oasis HLB μElution Plate 30 *μ*m desalting column (Waters, Milford, MA, USA), followed by drying in a freeze‐concentration dryer (Alpha 1–2 Ldplus/RVC2‐18 Cdplus, Christ, Germany). Ten percent of the samples were dissolved in 60 *μ*L of distilled water and their protein concentrations were estimated using a NanoDrop 2000 spectrophotometer (Thermo Fisher, Waltham, MA, USA).

The polypeptide samples were purified by Oasis MCX *μ*Elution Plate 30 *μ*m (Waters) and then applied to EASY‐nLC 1200 and Q‐Exactive (Thermo Fisher). The data acquisition software used was Thermo Xcalibur 4.0 (Thermo Fisher). The chromatographic separation time was 90 min and flow rate was 300 nL/min. MS scan range (m/z) was 350–1300, acquisition mode data‐dependent acquisition and the 20 strongest signals were selected in the parent ion for secondary fragmentation. Primary MS resolution was 70 000; the fragmentation method was high‐energy collision‐induced dissociation; the secondary resolution was 17 500; the dynamic exclusion time was 18 s. We used PEAKS Studio 8.5 to search for parameters. Dynamic modification selects oxidation (M), and acetyl (Protein N Terminus). The value of the enzyme name was set to unspecific. Precursor mass and fragment mass tolerance were set to 10 ppm and 0.05 Da, respectively. The results of MS were obtained directly from the neuropeptide gene database of the whitefly by the BLAST program.

### Effects of different temperatures on expression

Total RNA was extracted from 1–2 d old *B. tabaci* adults. The quantitative polymerase chain reaction (qPCR) primers (Supplementary data S2) were designed using Primer 3 (http://bioinfo.ut.ee/primer3-0.4.0/) and synthesized by Sangon Biotech (Shanghai, China). The primer efficiencies were between 90% and 110%. *Succinate dehydrogenase complex subunit A* (SDHA) was selected as the housekeeping gene for the qPCR (Li *et al*., 2013). The PCR conditions were 95 °C for 30 s, and 40 cycles of 95 °C for 5 s and 60 °C for 20 s, and at the end, these conditions were changed to 95 °C for 10 s, 65 °C for 60 s and 97 °C for 1 s.

### Statistical analysis

Three biological replicates, taken as three independent samples, were performed for each treatment and analyzed using Excel 2016 and SPSS 20. Student's *t*‐tests were used to determine the significance of differences between the treatment and control in the different temperature treatments. Means ± SE (standard error) were determined based on three biological replications.

## Results

### Neuropeptide and neurohormone catalog

**Fig. 1:**
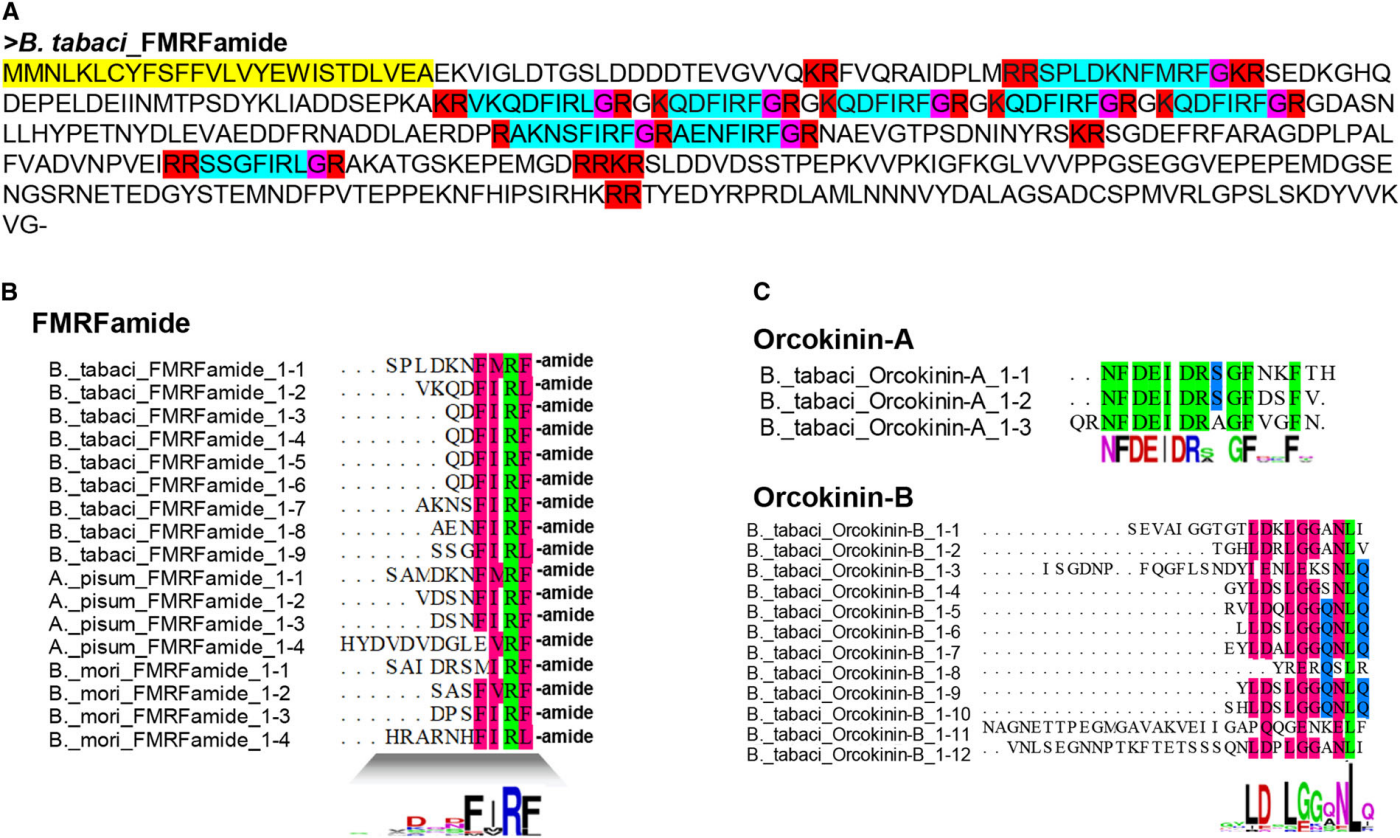
Alignment of the precursor and mature peptide sequences of FMRFamide and Orcokinin. (A) The yellow amino acid indicates the putative signal peptides, light blue indicates the mature peptide, red indicates cleavage signals and pink indicates amidation signals. (B,C) Sequence alignment of mature peptides of FMRFamide, Orcokinin‐A and Orcokinin‐B.

**Fig. 2:**
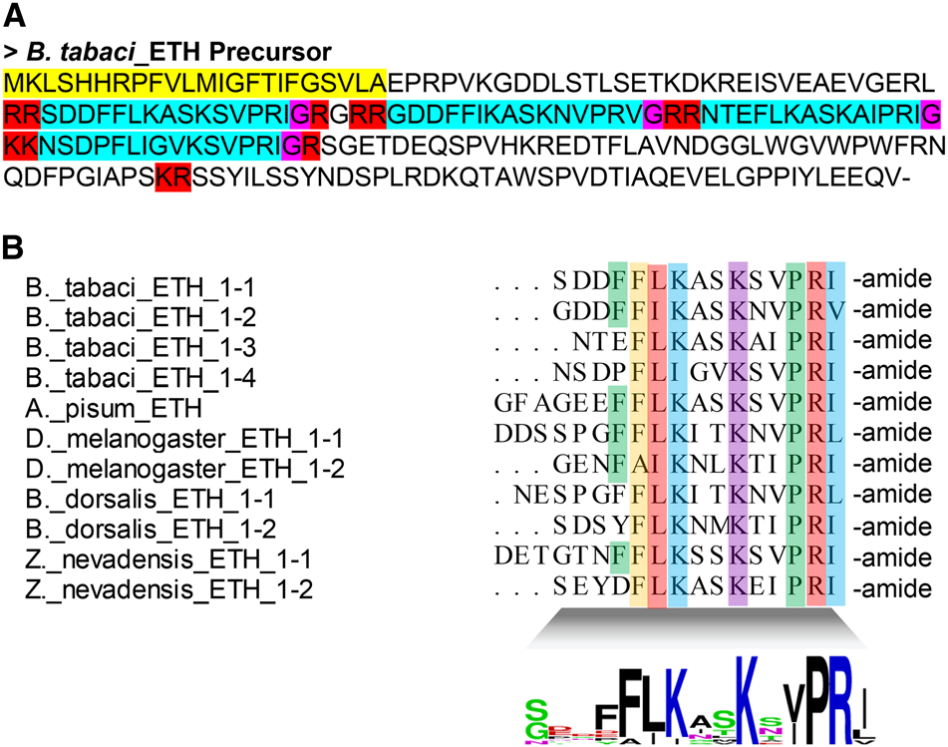
Alignment of the precursor and mature peptide sequences of ecdysis triggering hormone (ETH). (A) Deduced amino acid sequences of *Bemisia tabaci* ETH. The yellow amino acid indicates the putative signal peptides, light blue indicates the mature peptide, red indicates cleavage signals and pink indicates amidation signals. (B) Sequence alignment of mature peptides of ETH.

Based on the *B. tabaci* genome and transcriptome data, we predicted and annotated 38 neuropeptide precursors (Table 1; Supplementary data S3) compared to those from published data on five other species: *D. melanogaster*, *D. citri*, *N. lugens*, *T. castaneum* and *Z. nevadensis*. We identified peptide sequences associated with 16 different neuropeptide precursors, including Allatostatin A, Allatostatin B, CAPA, and 13 others (Supplementary data S3). All *B. tabaci* neuropeptide precursors showed the typical structure of neuropeptide precursors. In the *FMRFamide* gene (Fig. 1A; Supplementary data S3), we predicted nine mature peptides including four identical paracopies for QDFIRFs (Fig. 1A and B). Comparing all mature peptides to *A. pisum* and *B. mori*, we found the conserved motif xxxFxRF and detected RRSPLDKNFMRFamide and KQDFIRFamide by MS. These are partial sequences of the FMRFamide precursor (Supplementary data S3). We found that Orcokinin, as in other species (Chen *et al*., 2015), has two transcripts in *B. tabaci*, which have different conserved motifs (Fig. 1C). MS only detected the third paracopy of Orcokinin‐A. In addition, we identified four mature peptides by cleavage site in ecdysis triggering hormone (ETH) (Fig. 2A and B). The mature peptides were conserved with the xxxPRL (V/I) motif (Fig. 2B). Insulin‐related peptide (IRP) was also a conserved sequence. Two IRPs with the typical A‐ and B‐chains and cysteine bridges were also identified in *B. tabaci* (Supplementary data S3). Other neuropeptide precursor genes are annotated and shown in Supplementary data S3.

**Table 1:** Neuropeptide genes in *Bemisia tabaci* and other insects. The data of other insects are referred from *Acyrthosiphon pisum*, *Rhodnius prolixus*, *Tribolium castaneum*, *Drosophila melanogaster* and *Zootermopsis nevadensis*

Neuropeptide genes	Acronym	*B. tabaci*	*A. pisum*	*R. prolixus*	*T. castaneum*	*D. melanogaster*	*Z. nevadensis*
Adipokinetic hormone	*AKH*	+	+	+	2 genes	+	2 genes
Allatostatin A	*AST‐A*	+	+	+	−	+	+
Allatostatin B	*AST‐B*	+	+	+	+	+	+
Allatostatin CC	*AST‐CC*	+	+	+	−	+	+
Allatostatin CCC	*AST‐CCC*	+	+	+	+	+	+
Allatotropin	*AT*	+	+	+	+	−	+
Bursicon alpha	*Bur a*	+	+	+	+	+	+
Bursicon beta	*Bur b*	+	+	+	+	+	+
Cardio acceleratory peptide 2b	*CAPA*	+	+	+	+	+	−
Crustacean cardioactive peptide	*CCAP*	+	+	+	+	+	+
CCHamide 1	*CCHa 1*	+	+	+	+	+	+
CCHamide 2	*CCHa 2*	+	+			+	+
CNMamide 1	*CNMa 1*	+	−	+	+	+	+
CNMamide 2	*CNMa 2*	+					+
Corazonin	*Crz*	+	−	+	−	+	+
Diuretic hormone 31	*DH31*	+	+	+	+	+	−
Diuretic hormone 45	*DH45*	+	+	−	−	−	+
Ecdysis triggering hormone	*ETH*	+	+	+	+	+	+
Eclosion hormone 1	*EH 1*	+	+	+	+	+	+
Eclosion hormone 2	*EH 2*	+	+				+
FMRFamide	*FMRFa*	+	+	+	+	+	+
Insulin‐related peptide	*IRP*	2 genes	10 genes	4 genes	4 genes	7 genes	5 genes
Ion transport peptide short isform	*ITPs*	+	+	+	+	+	+
Ion transport peptide long isform	*ITP l*	+					+
Leucokinin	*LK*	+	+	+	−	+	+
Myossuppressin	*MS*	+	+	+	+	+	+
Natalisin	*NTL*	+	−	+	−	−	+
Neuroparsin	*NP*	+	−	+	+	−	+
Neuropeptide F	*NPF*	+	+	+	−	+	2 genes
Orcokinin‐A	*OK‐A*	+	+	3 genes	+	+	+
Orcokinin‐B	*OK‐B*	+					+
Pheromone Biosynthesis Activating Neuropeptide	*PBAN*	+	+	+	+	+	−
Proctolin	*Pro*	+	+	+	+	+	+
RYamide	*RYa*	+	−	+	−	−	+
SIFamide	*SIFa*	+	+	+	+	+	+
Short Neuropeptide F	*sNPF*	+	+	+	+	+	+
Tachykinin	*TK*	+	+	+	+	+	+

+, identified; −, not identified.

### GPCRs for neuropeptides

**Fig. 3:**
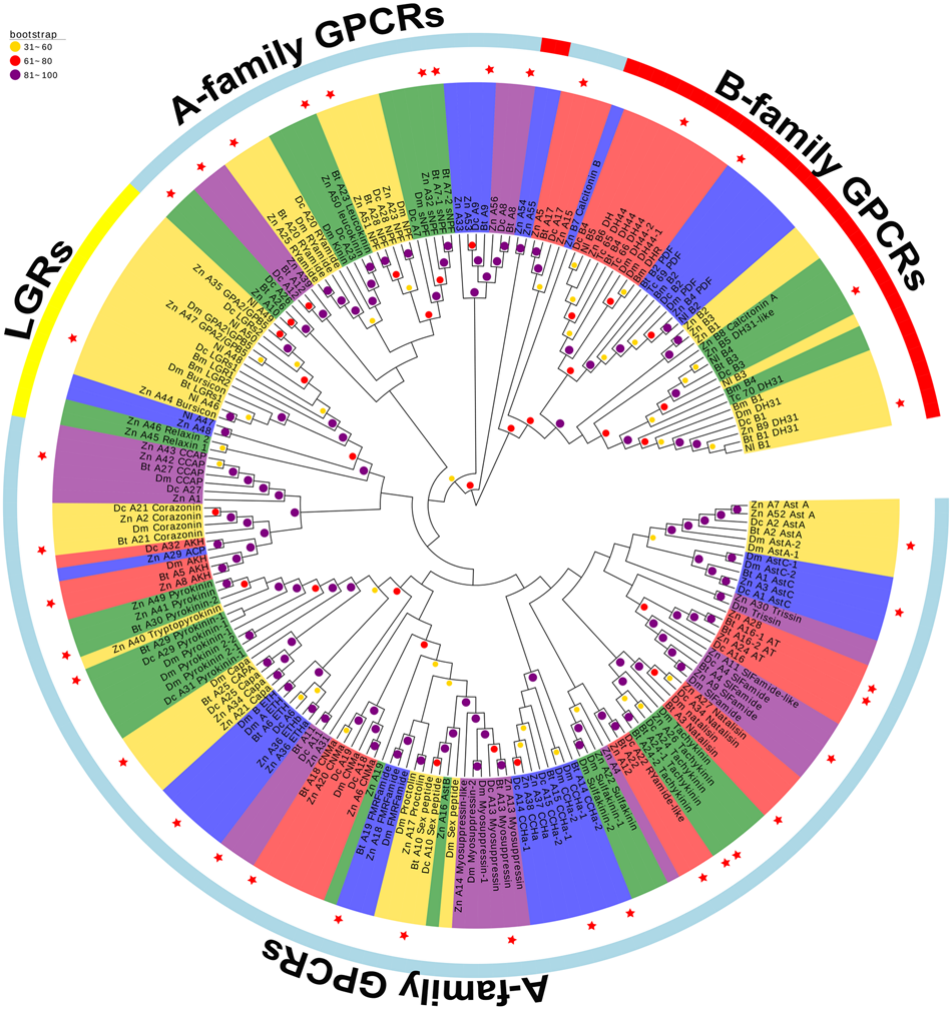
Phylogenetic tree of the G‐protein‐coupled receptors (GPCRs). The neuropeptide GPCRs of *Bemisia tabaci* are shown in red star. Bt, *B. tabaci*; Bm, *Bombyx mori*; Dc, *Diaphorina citri*; Dm, *Drosophila melanogaster*; Nl, *Nilaparvata lugens*; Tc, *Tribolium castaneum*; Zn, *Zootermopsis nevadensis*.

Based on the published neuropeptide receptors, we predicted 35 neuropeptide receptor genes (Table S1), including 30 A‐families GPCRs, four B‐families GPCRs, and one leucine‐rich repeat‐containing GPCRs (LGRs). We constructed a phylogenetic tree (Fig. 3) with the predicted receptors for *D. citri*, *D. melanogaster*, *N. lugens*, *T. castaneum*, *B. mori* and *Z. nevadensis*.

For family A GPCRs (Fig. 3), phylogenetic analysis allowed the identification of Ast C‐R (Bt A1), Ast‐B‐R (Bt A2), Natalisin‐R (Bt A3), SIFa‐R (Bt A4), AKH‐R (Bt A5), ETH‐R (Bt A6), sNPF‐R (Bt A7), Sex peptide‐R (Bt A10), Myosuppressin‐R (Bt A13), CCHamide (Bt A14 and Bt A15), AT‐R (Bt A16), CNMa‐R (Bt A18), FMRFa‐R (Bt A19), RYa‐R (Bt A20), Corazonin‐R (Bt A21), Leucoinin‐R (Bt A23), Tachykinin‐R (Bt A24), CCAP‐R (Bt A25), CAPA‐R (Bt A27), NPF‐R (Bt A28), and Pyrokinin‐R (Bt A29 and Bt A30). Although Trissin‐R and Proctolin‐R occur in *D. melanogaster* and *Z. nevadensis*, we did not find them in *B. tabaci* (Fig. 3). The transcript Bt A22 is closely related to RYamide like‐R (Dc A22) and Zn A12; considering that the likely ligand of Zn A12 was not found. Bt A22 could be an RYamide‐like‐R, given its sequence and configuration in the phylogenetic analysis. The transcripts Bt A8, Bt A9, Bt A11, Bt A12 and Bt A17 encode family A GPCRs that are grouped with orphan receptors from *D. citri* and *Z. nevadensis*.

In the B family, we identified Bt B1, Bt B2, and Bt B4 as DH31, PDF, and DH44 receptors, respectively. Bt B3 and the other three B family GPCRs were clustered on one branch. LGRs can be identified as three main types (types A, B, and C). However, in *B. tabaci*, we only predicted one Bt LGRs1 (Bursicon receptor), which was type A (Fig. 3).

### Expression of neuropeptides under different temperatures

**Fig. 4:**
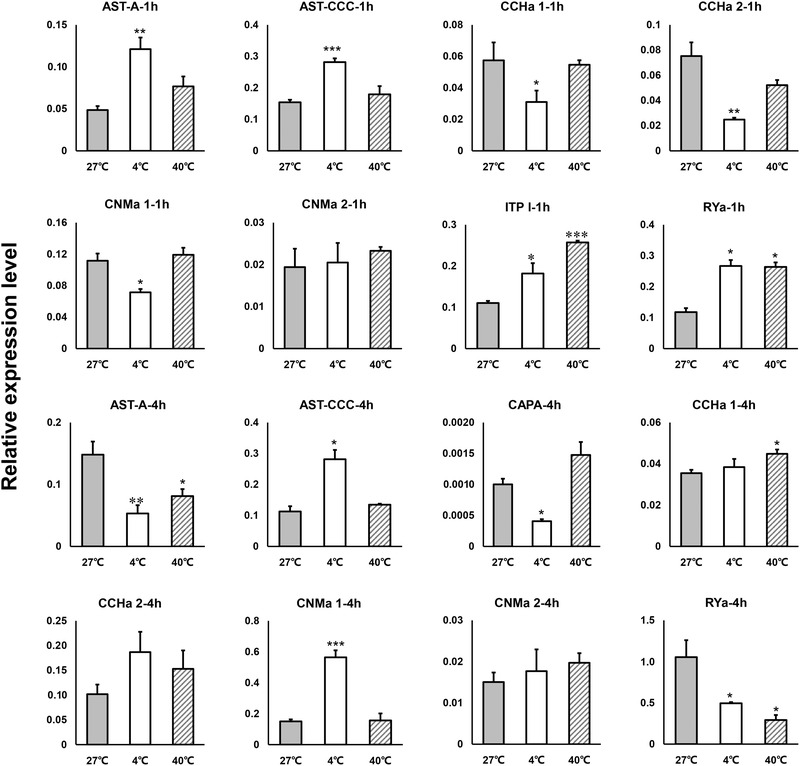
The expression of neuropeptide genes under temperature stress in *Bemisia tabaci*. Data are presented as means ± SE based on three independent experiments (*P < 0.05, **P < 0.01, ***P < 0.001, independent samples *t*‐test).

The stress responses of whitefly neuropeptides, under different temperature treatments, were measured using qPCR (Supplementary data S4). AST‐A, AST‐CCC, ITP long isoform (ITP l) and RYamide had the highest expression level at 1 h at 4 °C. CCHamide 1, CCHamide 2, and CNMamide 1 were significantly down‐regulated after 1 h at 4 °C. In the 40 °C treatment, ITP l and RYamide were significantly up‐regulated after 1 h. The expression of some neuropeptide genes at 4 h was different from that at 1 h. For example, AST‐A, RYamide decreased significantly in the 4 h‐4 °C treatment and in the 40 °C treatment. Interestingly, CCHamide 1 was significantly up‐regulated at 40 °C and CNMamide 1 had the highest expression level in the 4 h‐4 °C treatment (Fig. 4).

### Expression of neuropeptide receptors at different temperatures

**Fig. 5:**
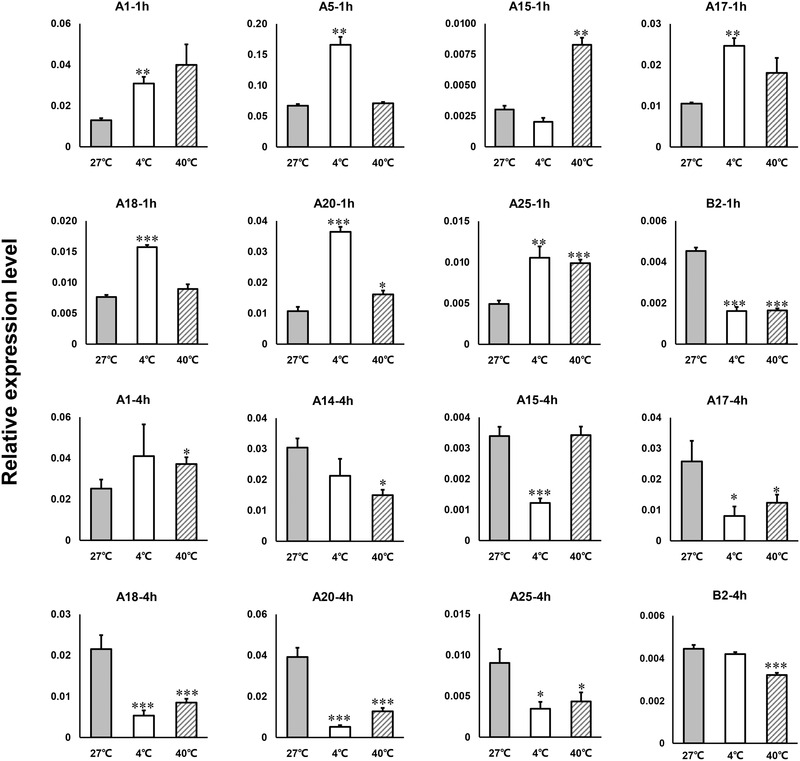
The expression of neuropeptide receptor genes under temperature stress in *Bemisia tabaci*. Data are presented as means ± SE based on three independent experiments (*P < 0.05, **P < 0.01, ***P < 0.001, independent samples *t*‐test).

We studied the expression of neuropeptide receptors in *B. tabaci* (Fig. 5), and found that many receptors such as A1 (Allatostatin C‐R), A5 (AKH‐R), A17 (CNMamide 1‐R), A18 (CNMamide 2‐R), A20 (RYamide‐R) and A25 (CAPA‐R) were up‐regulated after 1 h of 4 °C exposure. A20 (RYamide‐R) and A25 (CAPA‐R) were significantly up‐regulated after 1 h at 40 °C. Many genes, such as A15 (CCHamide‐1‐R), A17 (CNMamide 1‐R), A18 (CNMamide 2‐R), A20 (RYamide‐R) and A25 (CAPA‐R), had lower expression levels at 4 °C. A14 (CCHamide 2‐R), A17 (CNMamide 1‐R), A18 (CNMamide 2‐R), A20 (RYamide‐R), A25 (CAPA‐R) and B2 (PDF‐R), were also downgraded after 4 h at 40 °C. However, A1 (AST‐C‐R) was up‐regulated. The qPCR data for other neuropeptides and their receptors are shown in Supplementary data S4.

## Discussion

*B. tabaci* is a serious pest worldwide (Oliveira *et al*., 2001; González‐Zamora & Moreno, 2010; De Barro *et al*., 2011; Valle *et al*., 2012; Liu *et al*., 2015), and most current whitefly population management methods are unsatisfactory (He *et al*., 2007; Ahmad *et al*., 2010; Liang *et al*., 2012; Zheng *et al*., 2012). The use of neurological insecticides may represent a new approach for pest management (Audsley & Down, 2015). Identification of *B. tabaci* neuropeptides and their receptors are therefore important. Temperature is a critical environmental factor for *B. tabaci* and it can quickly adapt to temperature changes. Studying the invasion of *B. tabaci* into a new environment and determining the mechanisms by which the neuroendocrine system reacts to temperature resilience could be useful in the development of new management options.

We identified 38 neuropeptide precursor genes in *B. tabaci* that were similar to the neuropeptide precursors identified in other insect species (Amare & Sweedler, 2007; Huybrechts *et al*., 2010; Nässel & Winther, 2010; Veenstra, 2014; Ons, 2017). Many interesting neuropeptide precursor genes were found including ETH. The ETH precursor of *A. pisum* encodes only ETH while in *D. melanogaster*, *D. citri* and *B. dorsalis*, this precursor produces both ETH and pETH (Huybrechts *et al*., 2010; Nässel & Winther, 2010; Gui *et al*., 2017; Wang *et al*., 2018). We found four mature peptides in *B. tabaci* (Fig. 2A and B). Our future research will be aimed at determining if all four mature peptides are active and establishing the function of ETH. In other species, this precursor produces both NPF1a and NPF1b, but only NPF2 was found in *B. tabaci*. There is only one NPF in *A. pisum* and *N. lugens* (Huybrechts *et al*., 2010; Tanaka *et al*., 2014; Li *et al*., 2018). As only one NPF gene exists in these related species it is likely that the *B. tabaci* genome has only a single NPF gene. However, some neuropeptide genes may not have been identified due to incomplete genome and transcriptome data. Therefore, some of the neuropeptide sequences we predicted may be incorrect, especially at the 5′ end. This would affect our prediction of signal peptides. Second, the use of limited sequence homology may not have detected all of the genes in *B. tabaci*. Finally, during neuropeptide evolution, new neuropeptides may be acquired, and existing neuropeptides may be lost. The loss of genes may not be limited to *B. tabaci* but may also have occurred in other species of Hemiptera. In this case, these genes may be truly missing in *B. tabaci* (Veenstra, 2016). In general, many neuropeptide sequences are conserved in *B. tabaci* since it belongs to a relatively primitive group of Hemiptera (Misof *et al*., 2014).

Neuropeptides and their receptors play an important role in controlling various physiological processes. Some neuropeptides and receptors of *B. tabaci* are sensitive to temperature. AKH was up‐regulated at 4 °C for 1 h (Supplementary data S4). AKH may help maintain the normal biological activities of *B. tabaci* at low temperatures. AKH mobilizes energy substrates (lipids, trehalose or proline), and its function includes cardiostimulation and the inhibition of synthesis of RNA, fatty acids and proteins in the fat body (Vecera *et al*., 2007; Kodrík, 2008). Allatostatin A is also sensitive to low temperatures and is up‐regulated after 1 h at 4 °C and down‐regulated after 4 h at 4 °C (Fig. 4). The primary function of AST‐A may be myoinhibition. AST‐A also regulates aspects of feeding and metabolism in several other insect species (Lwalaba *et al*., 2010; Hergarden *et al*., 2012; Zandawala & Orchard, 2013; Hentze *et al*., 2015). Our results suggest that AST‐A is involved in low temperature adaptability in *B. tabaci*. CAPA peptides can stimulate Malpighian tubule secretion in other insects (Halberg *et al*., 2015). This secretion affects desiccation and cold stress tolerance in *D. melanogaster* (Terhzaz *et al*., 2015). We also found that A25 (CAPA‐R) was significantly up‐regulated after 1 h at 40 °C and 1 h at 4 °C (Fig. 5).

Neuropeptides act through receptors and, in many cases, GPCRs are specific for a particular neuropeptide. We found that RYamide and its receptors are equally sensitive to 4 °C and 40 °C. Its receptors were simultaneously up‐regulated at 1 h, while they were down‐regulated at 4 h (Figs. 4 and 5). In *Drosophila*, RYamide functions in regulation of water reabsorption (Veenstra *et al*., 2017). ITP was isolated as an ion transport peptide that seems to act as an antidiuretic hormone (Johard *et al*., 2009; Hermann‐Luibl *et al*., 2014). In *B. tabaci*, ITP l and RYamide were up‐regulated after 1 h at 4 °C (Fig. 4). This may have occurred because whiteflies can lower their temperature by adjusting the antidiuretic effect of evaporative cooling. Both ITP l and RYamide might be antidiuretic hormones. In the real‐time qPCR results, the expression levels of neuropeptides and their receptors were sometimes asynchronous. For example, CCHa 1 and CCHa 2 showed down‐regulation after 1 h at 4 °C, while CCHa 1 was up‐regulated after 4 h at 40 °C (Fig. 4). The receptor CCH1a‐R (A15) was up‐regulated after 1 h at 40 °C, and down‐regulated after 4 h at 4 °C. For CCH2a‐R (A14), it was down‐regulated simultaneously after 4 h under the 40 °C and 4 °C treatments (Fig. 5; Supplementary data S4). In contrast, the neuropeptide and receptor regulation feedback times were not synchronized. These differences may have several causes. First, there may be synergistic or antagonistic effects when the receptor responds to neuropeptides. Second, they may perform different functions separately. For example, ETH is associated with insect molting and also influences the reproductive capacity of *Drosophila* and *B. dorsalis* (Diao *et al*., 2016; Shi *et al*., 2017; Shi *et al*., 2019). CCHa2 can function in different diuretic processes in *R. prolixus*. CCHa2 enhances the serotonin‐induced secretion by Malpighian tubules and simultaneously inhibits serotonin‐induced absorption across the anterior midgut (Capriotti *et al*., 2019). Regulation of the diuresis of CCHa2 may affect insect temperature tolerance. Finally, one neuropeptide receptor may be activated by other neuropeptides. For example, the Myosuppressin receptor can be activated by both Myosuppressin and FMRF amide neuropeptides (Yamanaka *et al*., 2005; Yamanaka *et al*., 2006). Although the specific function of CNMamide is unclear (Jung *et al*., 2014), we found that CNMa 1, and its receptors A17 and A18, were sensitive to low temperatures. These findings suggest that CNMamide 1 functions at low temperatures in *B. tabaci*.

In conclusion, 38 neuropeptide genes were predicted and identified in *B. tabaci*. Among these, AKH, AST‐A, CAPA, CCHamide, RYamide and CNMamide were sensitive to low‐ or high‐temperature stress. This study provides neurophysiological information on how *B. tabaci* responds to temperature changes. More studies are needed to determine the specific mechanism(s) used by *B. tabaci* to adapt to temperature stress.

## Disclosure

The authors declare they have no conflicts of interest.

## Supporting information


**Supplementary data S1** Data of neuropeptide G‐protein‐coupled receptor (GPCR) genes. Bt, *B. tabaci*; Bm, *B. mori*; Dc, D*. citri*; Dm, *D. melanogaster*; Nl, *N. lugens*; Tc, *T. castaneum*; Zn, *Z. nevadensis*.

**Supplementary data S2** Primers used for real‐time quantitative polymerase chain reaction.

**Supplementary data S3** Predicted structures of neuropeptide precursors of *Bemisia tabaci*. Predicted signalpeptides (highlighted in yellow), cleavage signals (red), putative bioactive mature peptides (light blue), amidation signals (pink), N‐terminal Glutamate (Q) to Pyroglutamate (pQ) conversion (green) and cysteine residues (deep yellow) are indicated.

**Supplementary data S4** The expression of neuropeptide precursor and receptor genes under temperature treatment in *Bemisia tabaci*. The quantitative real‐time polymerase chain reaction analysis results of neuropeptides in *B. tabaci*. Data are presented as means ± SE based on three independent experiments (*P < 0.05, **P < 0.01, ***P < 0.001, independent samples *t*‐test).

**Table S1** Neuropeptide G‐protein‐coupled receptor (GPCR) genes putative identified from *Bemisia tabaci* comparison with *Diaphorina citri*, *Drosophila melanogaster* and *Zootermopsis nevadensis*.
