# Supplementary material for: Genome‐wide identification of neuropeptides and their receptor genes in Bemisia tabaci and their transcript accumulation change in response to temperature stresses
Source: Insect Sci. 2020 May 25;28(1):35–46. doi: 10.1111/1744-7917.12751 (PMC7818427; doi:10.1111/1744-7917.12751)
Supplement: Supplementary file 2 — Supplementary data S2 Primers used for real‐time quantitative polymerase chain reaction. [file INS-28-35-s002.pdf]

Supplementary data S2.

Primers used for real-time qPCR.

Neuropeptide precursor and receptor gene qPCR primers		
Gene name	Primer sequence	Primer efficiency
AKH-F	TCACAAATGCAATCCTCCTG	90.35%
AKH-R	GTGCATTCTTCTTGCGTGAC	
Allatostatin A-F	GATCCGACCGTACAACTTCG	103.75%
Allatostatin A-R	GTACATCTTGTTGCGCTTGC	
Allatostatin B1a-F	CCTGGGGTAAAAGAGATCCA	95.37%
Allatostatin B1a-R	TTGATTAGTGCGTCGTCAGC	
Allatostatin B1b/1c-F	AGCGTGGGAGAACCTGAAG	99.24%
Allatostatin B1b/1c-R	CCACTGGGACTGCTGCTC	
Allatostatin CCC-F	ACCAACCAAGCGAGAAAGAA	90.65%
Allatostatin CCC-R	GTAGTTGATCAGCGCCGTCT	
AllatostatinCC-F	CCTGTGTAGGCACATCATCG	93.98%
AllatostatinCC-R	TCTTGCGGTGTCTCATCTTG	
Allatotropin-F	GGTATGGGTGTGGGTGTGG	99.16%
Allatotropin-R	AGTAGCTCCGCATGGAGATG	
Bursicon alpha-F	TCAGGAGCTGGAACAGGACT	103.32%
Bursicon alpha-R	CATTCGTCCGATACAACACG	
Bursicon beta -F	CAAGCTCAAAATGCGAATCTC	90.63%
Bursicon beta -R	GGGCAATGTTTCACACTCCT	
CAPA-F	GGAAGATAGGCCACCCGTAT	108.75
CAPA-R	CGCTCATGATCACTTGTTGC	
CCAP-F	TCGCCGTCTTCTCATTTTTC	90.90%
CCAP-R	GGGTCGTACACTGCCTCTTC	
CCHamide1-F	AGGGGTGAAGGTGAGCCTAT	94.82%
CCHamide1-R	GAATGGCCGGTACTTGAGTC	
CCHamide2-F	ATATCCTCGACGACCAGACG	102.44%
CCHamide2-R	GATGCTGGCCAAGAGTCC	
CNMamide 1-F	GGCCAAGCAAGCTTTAGACC	93.87%
CNMamide 1-R	CACCCAGGTACTCGAAGAGG	
CNMamide2-F	CAACTACGGAGGTGGGATGT	102.25%
CNMamide2-R	AATCATCCTTGGGCATTGAG	
Corazonin-F	ATGCCGATGAGACAGTGG	96.75%
Corazonin-R	TGCGAGTACTGGAACGTCTG	
DH31-F	AGAAATGTTGGCGCGATTAG	91.35%
DH31-R	GCTGAGACCCAAATCCAGAC	
DH45-F	TTTCAAATGGCGGAACTTCT	105.73%
DH45-R	TCTTCGAGGCCCCTGTAGTA	

ETH-F	GTGAATGACGGTGGATTGTG	91.55%
ETH-R	CGCAAAGGTGAGTCGTTGTA	
EH 1-F	GCATGCGGAACTGCATACT	99.63%
EH 1-R	GGGGATCATTTTCCCTTTGT	
EH 2-F	AACTGCGCTCAGTGTGCGAA	103.42%
EH 2-R	CGGGAAGCTGACGCAGTATT	
FMRF-F	ATTTCATGAGGTTCGGCAAG	100.83%
FMRF-R	TCTGATGGCGTCATGTTGAT	
RYamide-F	GTGCGTGTACATCGGTTTTG	107.77%
RYamide-R	GCAATGACTCGACTTCGTGA	
ITP short-F	CATGTCTGGAGGCCCTTCT	90.42%
ITP short-R	GTTCCTCTTCTCACGCAGGT	
ITP long-F	TTCAAAGGCTGTTTGGAAGTT	90.56%
ITP long-R	GCCCCATGCAATTGCTTTAT	
Leucokinin-F	GTGGTCCCGCATTTTACAGT	96.89%
Leucokinin-R	CCAGAATGCCGGTTTCTTTA	
Myosuppressin-F	TGCTAGCAGCGGTTCAACT	99.40%
Myosuppressin-R	GGAACCTCCTCGATTGGATT	
Natalisin-F	AAAGCGGCAGCAGAGACTT	107.32%
Natalisin-R	GATCCACCTCGTCTTTCCAA	
Neuroparsin-F	ACTGTGTTTGCCGTGCATAG	108.09%
Neuroparsin-R	CTCCCAGTAACCGCAATGAT	
NeuropeptideF-F	AGCAGGACCATCGATGAAAG	91.63%
NeuropeptideF-R	AAACGTCCGAGCTATCCAAA	
Orcokinin A-F	TTCCCACCTCTACCCAGTTG	105.48%
Orcokinin A-R	ACCGAGATGTGGAACTCTGG	
Orcokinin B-F	GACCAACTAGGTGGGCAAAA	90.03%
Orcokinin B-R	CCAGAGCGTCGAGATACTCC	
PBAN-F	ACCTCATAGTGGCCGTCATC	96.35%
PBAN-R	TCCTCCAGGATATCCGACCT	
Proctolin-F	ACGAAGATTCCTGTGGATGC	94.48%
Proctolin-R	GAGCAGTGTTGGTCCTCCTC	
SIFamide-F	ATGGTGCTGACTCGTTTTCC	95.10%
SIFamide-R	TCTGAGCAATTCTCTGAGTCG	
sNPF-F	CTTGTCAATGCAACGCCTTA	90.56%
sNPF-R	GCCAGCTCGTTTAGTGCTTC	
Tachykinin-F	CTGCTGCTTCTGAGCCTCTT	90.48%
Tachykinin-R	CCTGGAGCTCGTAGTCGAAC	
IRP1-F	AAGGCGCTCTCTACCATCAA	112.30%
IRP1-R	GAGCAAGCGTTGTAACAGCA	
IRP2-F	TCGTGAGCGAAACTGAGATG	119.43%
IRP2-R	GCAGCATTCCTCGACAATTT	
dl-BtR-A1-F	CATGAACCCCATCCTCTACG	91.03%

dl-BtR-A1-R	AACACGCTGTTCTCCATGTG	
dl-BtR-A2-F	ATGATCTGCGCGCTTTATTT	105.36%
dl-BtR-A2-R	TCCTGGTGACCCTCTTCTTG	
dl-BtR-A3-F	CACATCCGTGAGACGACACT	93.52%
dl-BtR-A3-R	AGGAATGTCGGATTGTCTCG	
dl-BtR-A4-F	AGCAACGACTCCAACTCGAT	97.58%
dl-BtR-A4-R	GTCGGTCTTTGTGGAGAAGC	
dl-BtR-A5-F	GCCAAACCAGATCCACAGAT	109.54%
dl-BtR-A5-R	GCGTCCAACACACAAAGAAC	
dl-BtR-A6-F	CCCGATCCTCTACAACCTCA	104.25%
dl-BtR-A6-R	GTAGGAGCTGGAGCTGGTTG	
dl-BtR-A7-F	ACACCGTCACCAACCTCTTC	105.06%
dl-BtR-A7-R	ACGACCGACGAAGGAGTAGA	
dl-BtR-A8-F	ATCTGCTACCTGCCCATCAC	95.46%
dl-BtR-A8-R	TGGATTTATGCATGTGGTCAA	
dl-BtR-A9-F	CTGCCAAGAGATGGAGAAGG	90.65%
dl-BtR-A9-R	CGACAGCGTAAAGGTTCGAT	
dl-BtR-A10-F	AAGCGGGACAAACTCTTCAA	103.65%
dl-BtR-A10-R	ATGAGGAAGACGGTGACGAC	
dl-BtR-A11-F	CAAGATGTGCACCATCAACC	99.06%
dl-BtR-A11-R	CCGCGTATGTTCAAAGGTCT	
dl-BtR-A12-F	ACGTGACGATGCTGACCTTA	102.36%
dl-BtR-A12-R	CCTGTAGCGAAGACGTAGGC	
dl-BtR-A13-F	CTCGGCGATCAATTTCATTT	106.03%
dl-BtR-A13-R	TCAGCTCGTTATGCTGGTTG	
dl-BtR-A14-F	CGTGTTCATGCTGTGGTTCT	101.35%
dl-BtR-A14-R	TGATGCACGAGTTGAGGAAG	
dl-BtR-A15-F	ACCCCATTGCCCTCTATTTC	96.35%
dl-BtR-A15-R	TGATGCTTGATTTGGCATGT	
dl-BtR-A16-1-F1	GAGTACAAGCGGGCCTTTTT	98.78%
dl-BtR-A16-1-R1	GGTCCAGGTGGATGGTGTC	
dl-BtR-A16-2-F2	CTGCGTGGCGGGATTACT	94.85%
dl-BtR-A16-2-R2	CTGTGGCTGTTGCTGTTGTT	
dl-BtR-A17-F	TTGGAGTTCGACACGGAGAT	90.56%
dl-BtR-A17-R	AGTAGACCAGCGGGTTGATG	
dl-BtR-A18-F	CGTGGGTAACTTCCTGTCGT	108.56%
dl-BtR-A18-R	AGAAGAGGAAGCCGAAGTCC	
dl-BtR-A19-F	CTGCCCTCGAGGATATACCA	106.65%
dl-BtR-A19-R	GGTCGTCGTATTGGTCTGCT	
dl-BtR-A20-F	CTCTTCCAACCCTCCAAGTG	96.41%
dl-BtR-A20-R	ACGCAGTACTGGAGGACCAT	
dl-BtR-A21-F	GAGCGAGCGAAGATGAAATC	99.17%
dl-BtR-A21-R	CTGTCGGGTCTGAGGAAAAG	

dl-BtR-A22-F	TGAGAATTTCTGGCTGTTCCA	97.67%
dl-BtR-A22-R	AATGGTATTGAAAATATAGCCATTG	
dl-BtR-A23-F	GCCTGGAAATGCTCAGGATA	103.57%
dl-BtR-A23-R	GCCAACACAGTGCAAAGAGA	
dl-BtR-A24-F12	GTGAACGCGAGTATGGAACC	106.87%
dl-BtR-A24-R12	GTTGTACCCGGTCCCTGTCT	
dl-BtR-A25-F	GCAGCAGTATCCCTGGATGT	98.43%
dl-BtR-A25-R	AGATACCGCTCCATCGAGAA	
dl-BtR-A26-F	TCCTCAACGTTCTCCTGGTC	97.86%
dl-BtR-A26-R	AGAAGTGAGTGTCCGCGTTT	
dl-BtR-A27-F	CGCCAATCCCATCATTTATT	102.52%
dl-BtR-A27-R	AGCTGCTAGAATCCGTGCTC	
dl-BtR-A28-F	CACCTCATTGCCATGTCATC	107.88%
dl-BtR-A28-R	ACTTCTTGGGCAAAAGCTGA	
dl-BtR-A29-F	GCCTGCCAAGTGAAGATGAT	98.38%
dl-BtR-A29-R	AAGGCGTAGAGGACGGAGAT	
dl-BtR-A30-F	AACAACGCCAGCTTCAAGAG	103.45%
dl-BtR-A30-R	TTGTTGAGTTCGCCCATGTA	
dl-BtR-B1-F	ACACCGTATTGCGCCTTTAC	100.32%
dl-BtR-B1-R	TCAGGGCCATCGATAAACTC	
dl-BtR-B2-F	CTCGGGGTTGTTGTCCTAAA	94.65%
dl-BtR-B2-R	AGCTGCTCTCAGTGCTTTCC	
dl-BtR-B3-F	TCCCTGTCCTGATTGTAGCC	98.11%
dl-BtR-B3-R	GCATGAGGACTCATCCACCT	
dl-BtR-B4-F	TCCACCACATCCTCCTCTTC	96.45%
dl-BtR-B4-R	ACAGAAACCCTGCAAGGAGA	
dl-BtR-LGRs-F	GAATCTCGAATCGCCAAGAG	107.75%
dl-BtR-LGRs-R	TGCTCCAAAGTCACGAGTTG	
