# Supplementary material for: Genome‐wide identification of neuropeptides and their receptor genes in Bemisia tabaci and their transcript accumulation change in response to temperature stresses
Source: Insect Sci. 2020 May 25;28(1):35–46. doi: 10.1111/1744-7917.12751 (PMC7818427; doi:10.1111/1744-7917.12751)

Supplementary data S3.

Predicted structures of neuropeptide precursors of *Bemisia tabaci*. Predicted signal peptides (highlighted in yellow), cleavage signals (red), putative bioactive mature peptides (light blue), amidation signals (pink), N-terminal N-terminal Glutamate (Q) to Pyroglutamate (pQ) conversion (green) and cysteine residues (deep yellow) are indicated.

An alignment showing consensus sequence of *B. tabaci* and related insect putative mature peptides. The calculated consensus logo was shown at the bottom or side.

Mass spectrometry spectra verifying the presence of neuropeptides encoded in the precursors described, extracts from *B. tabaci*. Mass spectrum sequences are underlined and shaded.

AKH

> *B. tabaci* _ Adipokinetic Hormone（AKH）XM_019040727.1

MTCRTILVLLTNAILLSALCYAQVNFSPTWGKRTVTQEECTSKPSMELLMYLYKMIENEAQKISDCEKFRS-

A.pisum_AKH	QVNFTPTWGQ	-amide
B.tabaci_AKH	QVNFSPTW..	-amide
M.spermotrophus_AKH	QLTFSTGW..	-amide
N.giraulti_AKH	QLNFSTGW..	-amide
N.vitripennis_AKH	QLNFSTGW..	-amide
O.abietinus_AKH	QLNFSTGW..	-amide
P.puparum_AKH	QLNFSTGW..	-amide
T.podisi_AKH	QLNFSTGW..	-amide

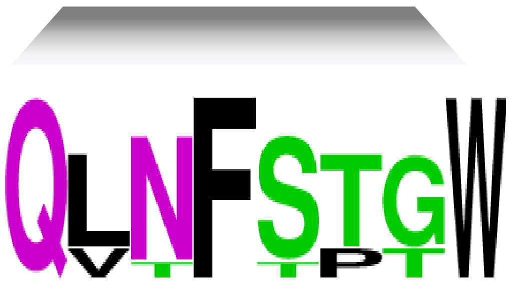

Allatostatin A

> *B. tabaci* _ Allatostatin A XM_019043162.1

MMLPLIMLVSLLRVTWGEEAEEADWESSRLGPDPGEDGPTSAEIYKRLYNFGLGKRAYSYVSEYKRLPVYNFGL
GKRSPRHYNFGLGKRADQYDDMLDDSYQTADLVKRIRPYNFGLGKRSPRQYNFGLGKRNKMYSFGLGKRAGLG
SDDYDQLDLDAYPYPPLPARPERSLHYNFGLGKRDPAPAKPQGPPASKESAQ-

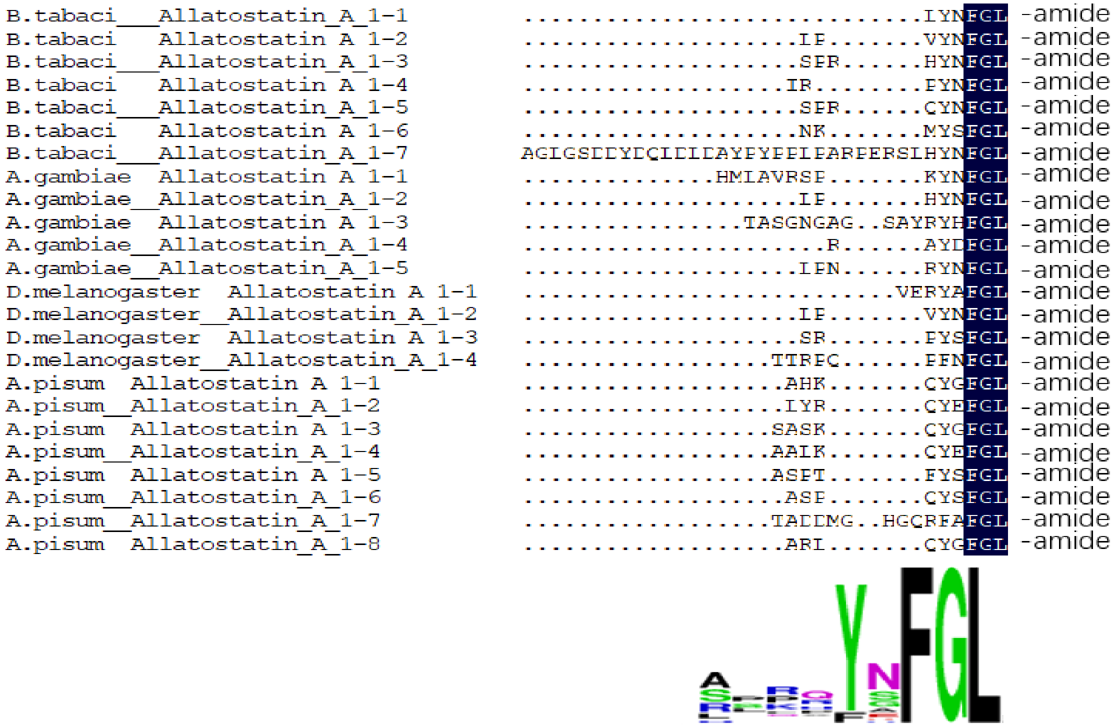

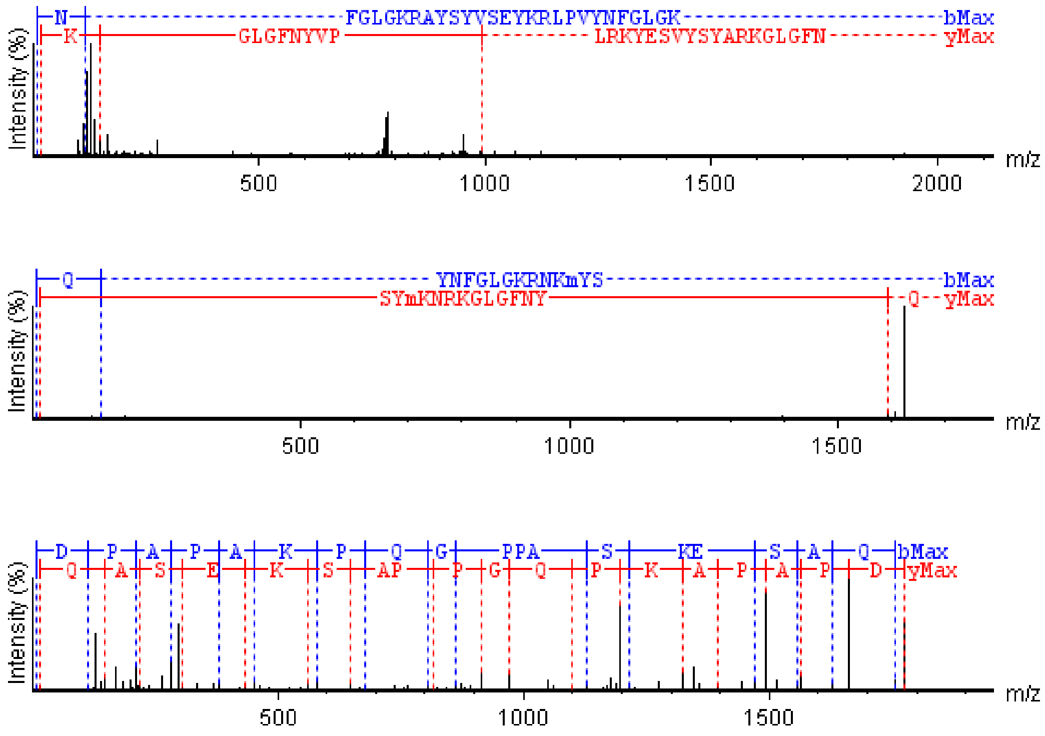

Allatostatin B

>*B . tabaci*_ Allatostatin B 1a XM_019044369.1

MHNLTVATALGLVSLLWLLALVAGETARQLSSSTSPLNQNQVQAASDPDSEGFEEREEEKRGWRDLQGGGWGKRAWENLKSGGWGKRSGSGGCSDSEQQSQWKRGWSDLQQAGWGKRGWSDLQGSGWGKRGWSNLHPGWGKRGWQNLHSSGWGKRAWQNLQGSWGKRDSPLDSQAQDDERLDEDKRSWNSLHSTWGKRSTSDWRSFGGSWGKRDPAWHNLKGVWGKRSLADDALIKFTPNIGYDDVANEYSLED-

> *B. tabaci*_ Allatostatin B 1b XM_019044370.1

MHNLTVATALGLVSLLWLLALVAGETARQLSSSTSPLNQNQVQAASDPDSEGFEEREEEKRGWRDLQGGGWGKRAWENLKSGGWGKRSGSGGCSDSEQQSQWKRGWSDLQQAGWGKRGWSDLQGSGWGKRGWSNLHPGWGKRGWQNLHSSGWGKRAWQNLQGSWGKRDSPLDSQAQDDERLDEDKRSWNSLHSTWGKRSTSDWRSFGGYDDVANEYSLED-

> *B. tabaci*_ Allatostatin B 1c XM_019044372.1

MHNLTVATALGLVSLLWLLALVAGETARQLSSSTSPLNQNQVQAASDPDSEGFEEREEEKRGWRDLQGGGWGKRAWENLKSGGWGKRSGSGGCSDSEQQSQWKRGWSDLQQAGWGKRGWSDLQGSGWGKRGWSNLHPGWGKRGWQNLHSSGWGKRAWQNLQGSWGKRDSPLDSQAQDDERLDEDKRSWNSLHSTWGKRSTSDWRSFGGES-

B.tabaci__Allatostatin_B_1-1	. . . . GWRDLQGGGW-amide
B.tabaci__Allatostatin_B_1-2	. . . . AWENLKSGGW-amide
B.tabaci__Allatostatin_B_1-3	. . . . GWSDLQQAGW-amide
B.tabaci__Allatostatin_B_1-4	. . . . GWSDLQGSGW-amide
B.tabaci__Allatostatin_B_1-5	. . . . GWSNLHPG.W-amide
B.tabaci__Allatostatin_B_1-6	. . . . GWQNLHSSGW-amide
B.tabaci__Allatostatin_B_1-7	. . . . AWQNLQGS.W-amide
B.tabaci__Allatostatin_B_1-8	. . . . SWNSLHST.W-amide
B.tabaci__Allatostatin_B_1-9	. . . . . STSDWRSFGGS.W-amide
B.tabaci__Allatostatin_B_1-10	. . . . DPAWHNLKGV.W-amide
A.pisum__Allatostatin_B_1-1	. . . . AWRDLQTAGW-amide
A.pisum__Allatostatin_B_1-2	. . . . GWQNLKTT.W-amide
A.pisum__Allatostatin_B_1-3	. . . . AQDWQNLHSS.W-amide
A.pisum__Allatostatin_B_1-4	. . . . QGWQKLHGG.W-amide
A.pisum__Allatostatin_B_1-5	. . . . GWKDMQSGGW-amide
A.pisum__Allatostatin_B_1-6	. . . . SWDNFQGS.W-amide
D.melanogaster__Allatostatin_B_1-1	. . . . AWQSLQSS.W-amide
D.melanogaster__Allatostatin_B_1-2	. . . . AWKSMNVA.W-amide
D.melanogaster__Allatostatin_B_1-3	. . . . RQAQGWNKFRGA.W-amide
D.melanogaster__Allatostatin_B_1-4	. . . . EPTWNNLKGM.W-amide
D.melanogaster__Allatostatin_B_1-5	. . . . DQWQKLHGG.W-amide
A.gambiae__Allatostatin_B_1-1	. . . . SWSKMNAA.W-amide
A.gambiae__Allatostatin_B_1-2	LSGGNRNSGWTKFGAA.W-amide
A.gambiae__Allatostatin_B_1-3	. . . . EPGWNNLKGL.W-amide

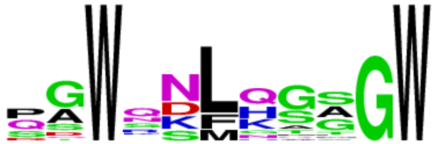

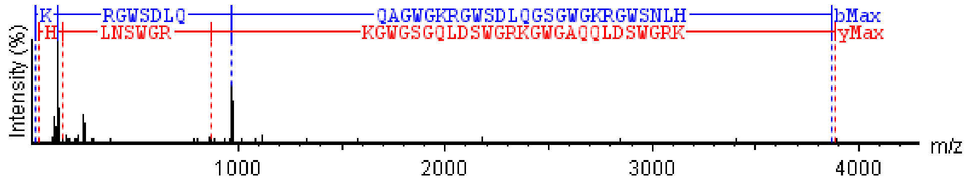

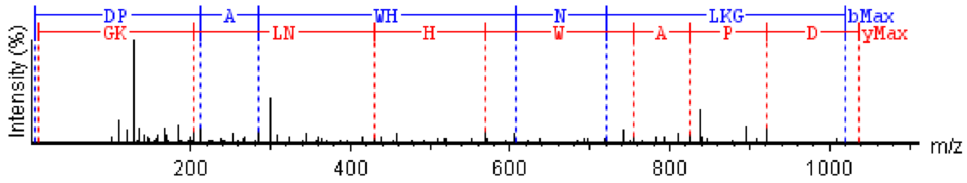

Allatostatin CC

> *B. tabaci_* Allatostatin CC XM_019053340.1

MGLFVTVLLLLLTAVSSLCRHIIDPTLGLDRTQEYALIKRDAQDETPQEVAFDEYPVVVPKRAAMLLDRIMSALQ
KAVDDDNSMSSKREPKFIRGNKTYWRCYFNAVSCF-

```
B.tabaci_Allatostatin_CC         EPKFIRGNKTYWRCYFNAVSCF
D.citri__Allatostatin_CC         .........TYWRCYFNAVSCF
N.viridula__Allatostatin_CC      .........IYWRCYFNAVTCF
A.pisum__Allatostatin_CC         NQQ..KG.RLYWRCYFNAVSCF
T.castaneum__Allatostatin_CC     .........VYWRCYFNAVTCF
C.floridanum_Allatostatin_CC     .GQ.AKG.RVYWRCYLNAVTCF
C.glomerata_Allatostatin_CC      .GR.ING.SVYWKCYFNAVTCF
C.rubecula_Allatostatin_CC       .GR.ING.SVYWKCYFNAVTCF
```

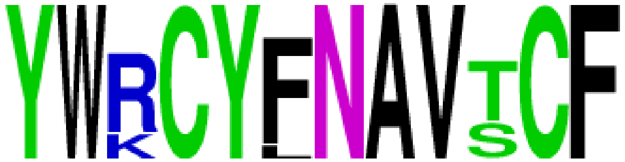

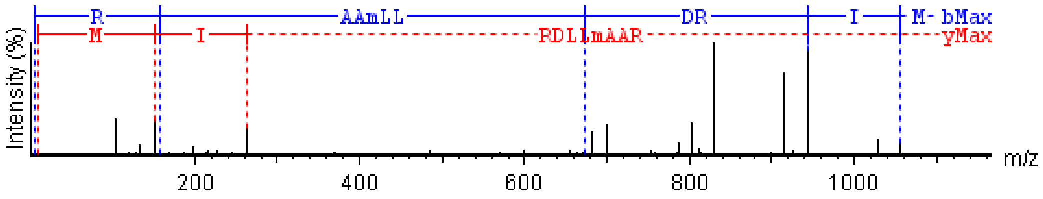

Allatostatin CCC

>*B. tabaci*_ Allatostatin CCC XM_019053899.1

MTTIARTIVFLLISMLSFTWVIGKALDQPSEKERLYNEIDQLVDDDGSAETALINYLFAKQIANRLRSQMDVTE
LQRKRSYWKQCAFNAVSCFG-

B.tabaci___Allatostatin_CCC	.SYWKQCAFNAVSCF-amide
D.citri__Allatostatin_CCC	.SYWKQCAFNAVSCF-amide
A.pisum__Allatostatin_CCC	.SYWKQCAFNAVSCF-amide
Z.nevadensis__Allatostatin_CCC	.SYWKQCAFNAVSCF-amide
Locusta__Allatostatin_CCC	.SYWKQCAFNAVSCF-amide

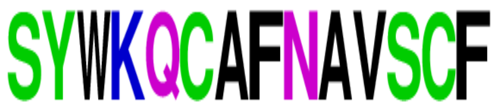

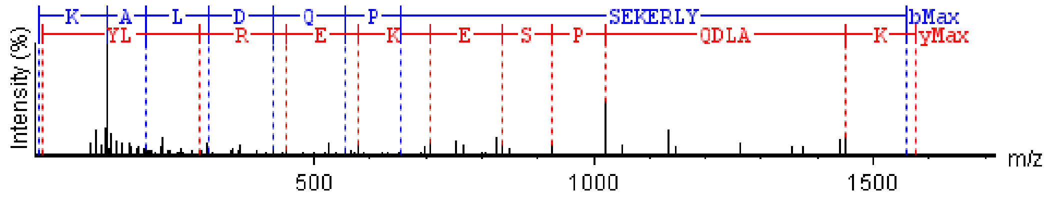

Allatotropin

> *B. tabaci* Allatotropin XM_019055215.1

MVRPMLRLSAHLVWVWVWVWVAMLIGASLVEEAEAQAQRSISMRSYYRGGGGKTPARTIRGFKQAALST
ARGFGKRDSPLTLPLAVPMARDDLEMAASQLQLNNRDSLPLEWILEGMQSNPVLARLFFQRLIELNHDATAA
AELSSNEALSPLYIAEKSKDDPPQRQDPDDVIM-

```
B.taba_i__Allatotropin          GFKQ..AALSTARGF -amide
B.mori__Allatotropin            .FKN..VEMMTARGF -amide
D.citri__Allatotropin           GFRDKVASLQTARNF -amide
R.prolixus_Allatotropin         GFKN..VQLSTARGF -amide
A.pisum_a_Allatotropin          GFKN..MDLSTARGF -amide
T.pretiosum_Allatotropin        GYQP..DQLMTAISF -amide
N.vitripennis_Allatotropin      GFQP..EYISTAYGF -amide
P.puparum_Allatotropin          GFQP..EYISTAYGF -amide
```

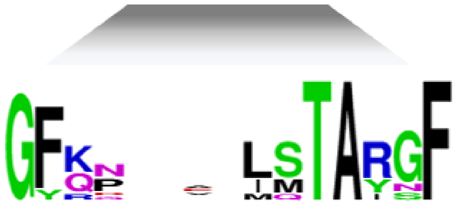

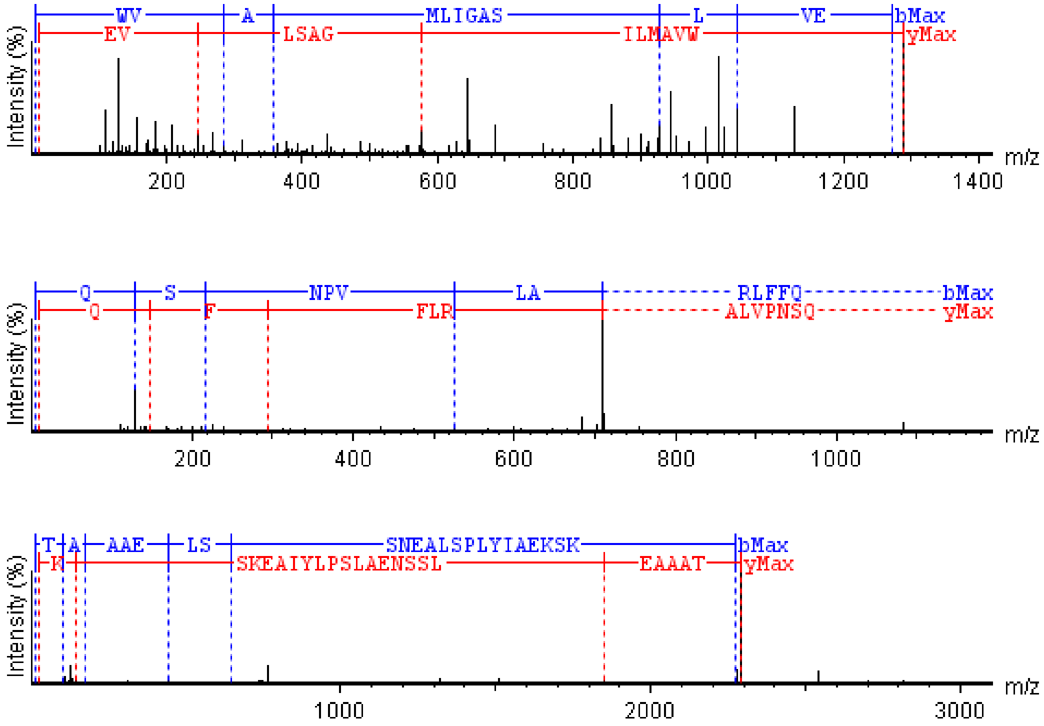

Bursicon alpha

>*B.tabaci* _Bursicon alpha XM_019055262.1

MFRSWNRTFLVWWLCLVLDGLVLGQDSPRVVSDECTVTPVLHVLQYPGCVPKPIPSFACTGRCSSYIQVSGSKIWQMERSCMCCQESGEREASVSLFCPKARPGEKKFRKVTTKAPLECMCRPCTSIEESSVVPQEIVNYAGEQPISGHFAKPE-

```
B.tabaci_Bursicon_alpha     ...............................................QDSPRVVS.....DECTVTPVLHVLQYPGCVPKPIPSFACTGRCSSYI..QVS
A.pisum_Bursicon_alpha      ...............................................DNGNGVVVTARSSDDCQVTPVIHVLQYPGCVPKPIPSFACTGRCSSYL..QVS
B.mori_Bursicon_alpha       ...............................................HEVQLPPGTKFFCQECQMTAVIHVLKHRGCKPKAIPSFACIGKCTSYV..QVS
N.viridula_Bursicon_alpha   ..............................................QNDGTKRPA.....DECQVTPVIHVLQYPGCVPKPIPSFACTGRCSSYI..QVS
N.lugens_Bursicon_alpha     .............................................FQEDAKPAVS....SDECQVTPVIHVLQYPGCVPKPIPSFACTGKCSSYL..QVS
A.armilla_Bursicon_alpha    CPLDARSTVHGFFDFINLPSCARYVQVFSSSTSKCAVVTGLLICLLYDNVKAILG....VDECRAVPVFHVLKRDGCLPRTIPSFACRGRCSSYLFPQVS
C.solmsi_Bursicon_alpha     ....................................................KVG....VDDCQITPVIHVLQYSGCVPKPIPSFACIGQCSSYL..QIS

B.tabaci_Bursicon_alpha     GSKIWQMERSCMCCQESGEREASVSLFCPKARPGEKKFRKVTTKAPL.ECMCRPCTSIEESSVVPQEIVNYAGEQP..ISGHFAKPE..
A.pisum_Bursicon_alpha      GSKIWQMERSCMCCQESGEREASVSLFCPKAKQGEKKFRKVTTKAPL.ECMCRPCTGIEESAVIPQEMSNYAADEPP.INGHFSKSI..
B.mori_Bursicon_alpha       GSKIWQMERTCNCCQESGEREATVVLFCPDAQNEEKRFRKVSTKAPL.QCMCRPCGSIEESSIIPQEVAGYSEEGP..LYNHFRKSL..
N.viridula_Bursicon_alpha   GSKIWQMERSCMCCQESGEREASVTLFCPKAKPGEKKFRKVMTKAPL.ECMCRPCTSVEESAVIPQEIAGLTDEGP..LNQHFVKPQ..
N.lugens_Bursicon_alpha     GSKIWQMERSCMCCQESGEREASVSLFCPKAKPGEKKFRKVNTKAPL.DCMCRPCTGVEETSVIPQEIAGYADEGP..MNNHFRLHSSR
A.armilla_Bursicon_alpha    GSKFWQMERSCMCCQESGEREASVSLFCPNPKPGERKFLKVRSTFPVHQSIC.....................................
C.solmsi_Bursicon_alpha     GSKIWQMERSCMCCQESGEREASISLFCPKAKAGERKFRKVITKAAL.DCMCRPCTDIEEYAIIPQEIADLADNGPFTSAAHFRHTL..
```

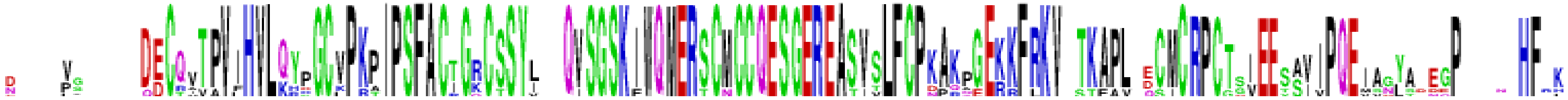

Bursicon beta

>*B. tabaci*_Bursicon beta XM_019055040.1

--------RREARKCGGRGKDVGRSHPLRPFHSRSPPTAHPQHSIKNKLKMRISSIVLWASFLMVKCSNEEECETLPSEIHITKEEFDELGRLQRTCNGDVAVSKCEGACNSQ
VQPSVATPNGFLKECFCCRESYLRERVITLDHCYDPDGIRITVEGKATMDIKLREPDQCKCYKCGDYSR

```
B.tabaci_Bursicon_beta       ......CETLPSEIHITKEEFDELGRLQRTCNGDVAVSKCEGACNSQVQPSVATPNGFLKECFCCRESYLRERVI
A.pisum_Bursicon_beta        ......CETLPSELHIIKEEFDELGRLQRTCNGDIAVNKCEGACNSQVQPSVITPSGFLKECYCCRETFLRERMI
B.mori_Bursicon_beta         ......CETVASEVHVTKEEYDEMGRLLRSCSGEVSVNKCEGMCNSQVHPSISSPTGFQKECFCCREKFLRERLV
D.citri_Bursicon_beta        ......CETLPSEIHIIKEEFDELGRLQRTCNADIAVNKCEGACNSQVQPSVITPNGFLKECYCCRESYLRERVI
N.lugens_Bursicon_beta       ......CETLPSEIHITKEEFDELGRLQRTCNGDVAVNKCEGACNSQVQPSVVTPTGFLKECFCCRESFLRERTI
D.alloeum_Bursicon_beta      ......CETLMSDIHVTKEEFDEGGILLRTCSDDLSVTKCEGFCNSQVQPSIMTATGFLKDCYCCRESYLKERNI
L.clavipes_Bursicon_beta     ......CETLQSEIHIAKDEKDETGGLIRTCSTDILVTKCEGSCNSQVQPSVKTSTGFLKECFCCRESYLKERLI
M.demolitor_Bursicon_beta    ......CETLMSEVHITKDEYDNSGGLIRTCSDDVSVTKCEGYCNSQIQPSVISSTGFVKECYCCRESYFKEKVV

B.tabaci_Bursicon_beta       TLDHCYDPDGIRITVEGKATMDIKLREPDQCKCYKCGDYSR
A.pisum_Bursicon_beta        ALTHCYDPDGVRLTSDKIATLEVKLKEPADCKCFKCGDFSG
B.mori_Bursicon_beta         TLTHCYDPDGIRFEDEENALMEVRLREPDECECYKCGDFSR
D.citri_Bursicon_beta        TLTHCYDPDGMRLTSEKMATLDIKLKEPADCKCYKCGDYSR
N.lugens_Bursicon_beta       TLTHCYDPDGMRLTGAPHSQMDVRLREPAECKCYKCSDYSR
D.alloeum_Bursicon_beta      ILHHCYSPDGIKLVESETATMEIKLREPADCECFKCGDFSR
L.clavipes_Bursicon_beta     LLDHCYDVDGVRLEGEILGTMEIKLREPAECKCTKCGGFSR
M.demolitor_Bursicon_beta    KLNHCYNSDGEKMETEKYATMEIKLREPHDCKCYKCTDFLR
```

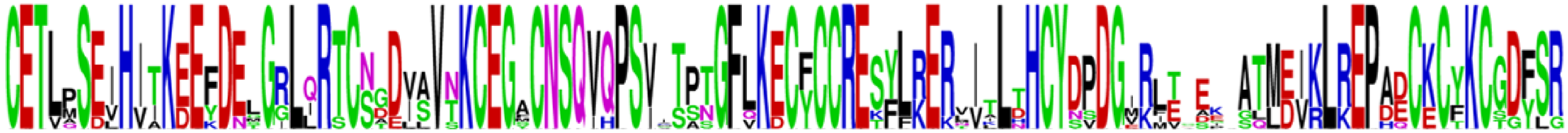

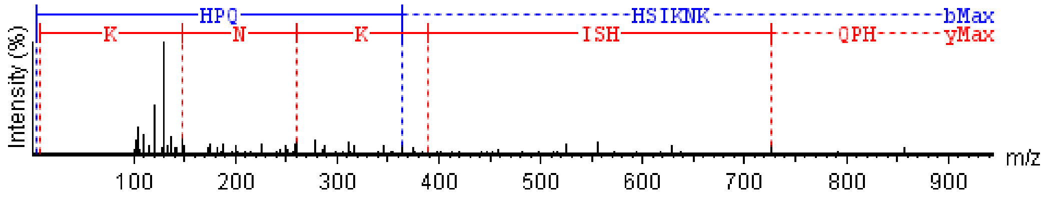

CAPA

>*B. tabaci*_CAPA XM_019040406.1

MSESIMAYFLVLCTVLVAVNQAQGLVSSNARSRRETKGLFPFPRVGRRGGEMAWMLGSNDLMDRQVKKQGLMAFPRVGRSGNAF
NDRVEGGALWIGPRLGRDIRLNIPKDISPWTLITLREFEAPPESETNGAPHETREEIFDYDLQASPKQQDISNNISH-

B.tabaci_CAPA_1-1	.........ETKGLFPFPRV	-amide
B.tabaci_CAPA_1-2	.........QG..LMAFPRV	-amide
B.tabaci_CAPA_1-3	SGNAFNDRVEGGALWIGPRL	-amide
N.viridula_CAPA_1-1	..........DAGLFPFPRV	-amide
N.viridula_CAPA_1-2	.........EQ..LIPFPRV	-amide
N.viridula_CAPA_1-3	SGPKRNGASGNGGLWFGPRL	-amide
P.puparum_CAPA_1-1	.........WT..MFPFPRV	-amide
P.puparum_CAPA_1-2	..........SFGLVKYPRV	-amide

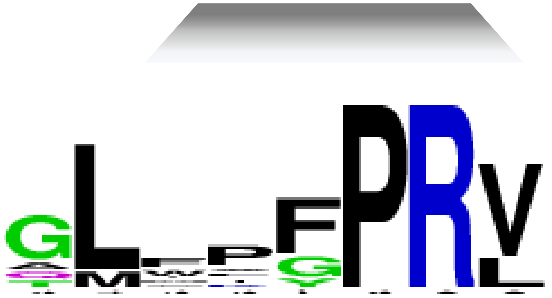

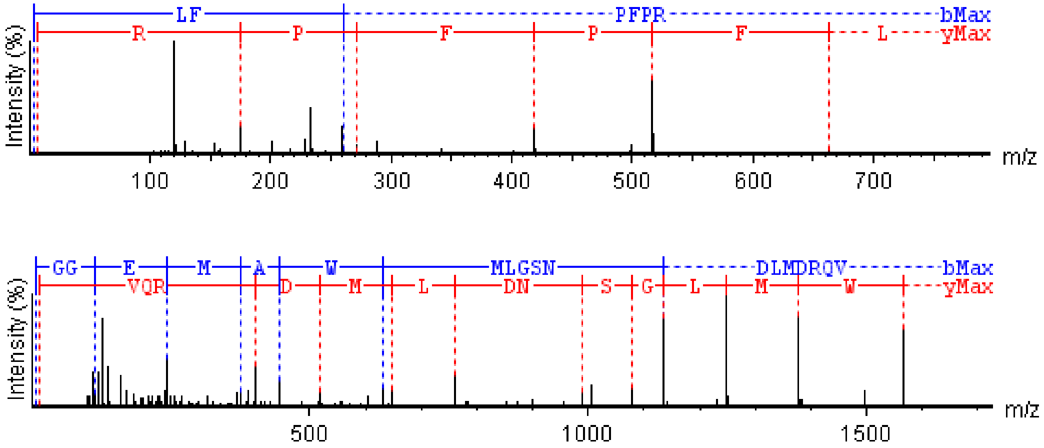

CCAP

***>B. tabaci*_CCAP XM_019043555.1**

MRWSQSLPCLLLLAVFSFFFVLVKADDVVIQKRAYNQELEEAVYDPKKKKPFCNAFTGCGRKRSDESMPLIDLNAEPVEEISR
QILSEAKLWEAIQEARLELLKKHAALEKLQNARLEAKGLPFTMMRRRRTSYKPY-

B.tabaci_CCAP	PFCNAFTGC -amide
A.pisum_CCAP	PFCNAFTGC -amide
B.mori_CCAP	PFCNAFTGC -amide
D.citri_CCAP	PFCNAFTGC -amide
P.stali_CCAP	PFCNAFTGC -amide
T.castaneum_CCAP	PFCNAFTGC -amide
L.crustacean_CCAP	PFCNAFTGC -amide

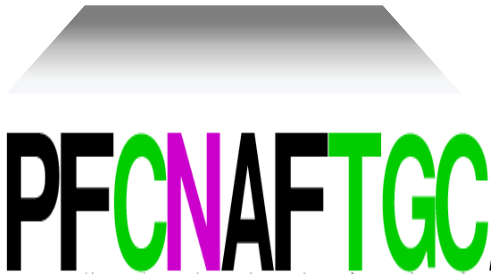

CCHamide-1

>*B. tabaci* _CCHamide 1 XM_019062177.1

MSLLQVLYLLVLASACIAAVSGSCLNYGHSCWGAHGKRSGSGSGSEDVSLDNWLISRLLAEGSRRGVPSSTMTRQTPERNWAAAPS
LPHKVNLQAIRWLTRGRFPFRLLEENLTRGEGEPILGTSEQEMNEIATGSDSSTGHSKDQELVLLEQPPKLIKILDQP-

B.tabaci_CCHamide_1	.....SCLNYGHSCWGAH	-amide
A.pisum_CCHamide_1	.....GCAMFGHSCYGAH	-amide
D.citri_CCHamide_1	CAAGSACLSYGHSCWGAH	-amide
N.viridula_CCHamide_1	......CMSYGHSCWGAH	-amide
Z.nevadensis_CCHamide_1	...TGSCLSYGHSCWGAH	-amide
A.armilla_CCHamide_1	.....SCAQYGHSCWGGH	-amide
C.solmsi_CCHamide_1	.....SCAQYGHSCWGGH	-amide
D.collaris_CCHamide_1	.....SCLSYGHSCWGAH	-amide
M.spermotrophus_CCHamide_1	.....SCLSYGHSCWGAH	-amide

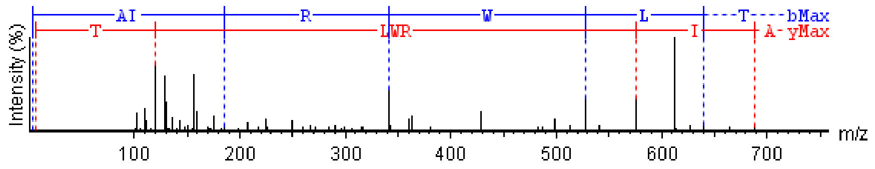

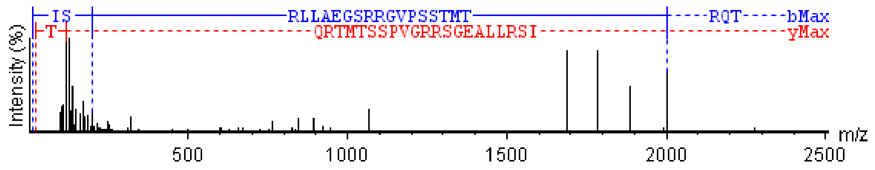

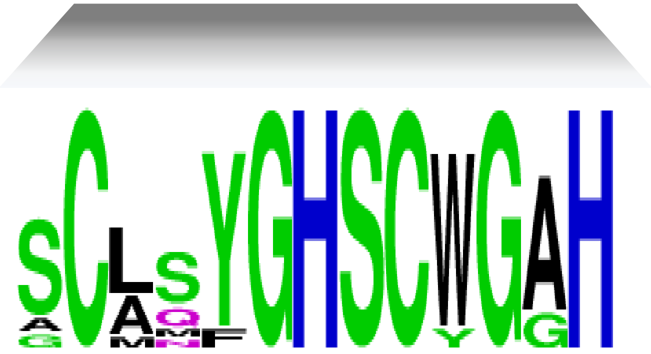

CCHamide-2

>*B. tabaci* _CCHamide2 XM_019047280.1

MRAACAETLFTLLGLNLLCHVFSAADASAKRGCSAFGHSCFGGHGKRFDPEAMEVGAGAAAAMADILDDQTTRDRDRDRNN
RDQDLGLLASIAAPRDLATASGNSQNILKALMDCQAIKQMCVTQAIGVSKIKRPSDTSSYNTEFSDNYH-

B.tabaci_CCHamide-2	.GCSAFGHSCFGGH	-amide
D.citri_CCHamide-2	.GCASFGHSCFGGH	-amide
N.viridula_CCHamide-2	GGCSSFGHSCFGGH	-amide
Z.nevadensis_CCHamide-2	.GCSAFGHSCFGGH	-amide
C.floridanum_CCHamide-2	.GCSAFGHSCYGGH	-amide
D.collaris_CCHamide-2	.GCAAYGHSCFGGH	-amide
L.clavipes_CCHamide-2	.GCGAFGHSCYGGH	-amide
M.spermotrophus_CCHamide-2	.GCSAFGHSCYGGH	-amide

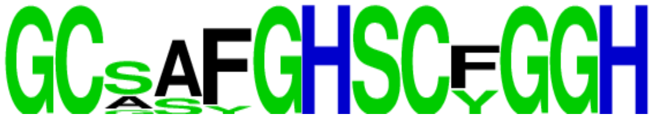

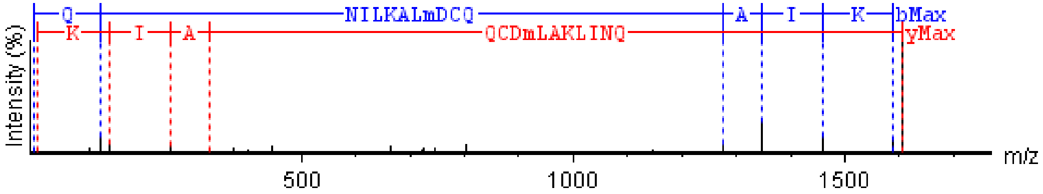

CNMamide

> *B.tabaci*_CNMamide 1 XM_019053859.1

MAQLTGMSHSLLISLVIVCTCLSSLVKAEEELKQAPAFTDSEKPFKLGELLQRLQAAKQALDRERIIEQASKDGKDLFEYLGENYYGSDSSDTPIAPPGYRVRLISTPEAARDDYLYG
KDKRGNYMTLCHFKICNMGRKRWKSWP-

> *B.tabaci*_CNMamide 2 XM_019045455.1

MSEYLLLLFFSTLGLVAPQRSLTRDGSYSKQSPELTESTQQQTGNEIQRILETLRLSRELSEEGRQADDWRQESGLDYPAESSGNYGGGMSAVPSLFNQRLMMTLRSMPKDDSAL
GTGKRGKYMSLCHFKICNMGRKRWKAWP

B.tabaci_CNMamide_1a	GNYMT.LCHFKICNM	-amide
B.tabaci_CNMamide_1b	GNYMT.LCHFKICNM	-amide
B.tabaci_CNMamide_2	GKYMS.LCHFKICNM	-amide
D.citri_CNMamide	GSYMA.LCHFKICNM	-amide
N.viridula_CNMamide	ASYMS.LCHFKICNM	-amide
P.stali_CNMamide	ASYMS.LCHFKICNM	-amide
C.glomerata_CNMamide	TSYMA.LCHFKICNM	-amide
C.rubecula_CNMamide	TSYMA.LCHFKICNM	-amide
Z.nevadensis_CNMamide_A	GNYMS.LCHFKICNM	-amide
Z.nevadensis_CNMamide_B	GNYPPPLCYFKICNM	-amide

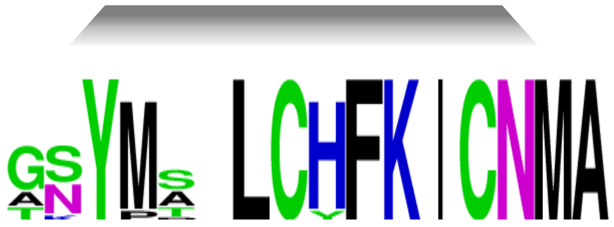

Corazonin

> *B . tabaci_* Corazonin XM_019042986.1

MPMRQWLICVVVAIWLADSLAQTFQYSHGWTSGKKRSGSGLLGPGALSAASASARRDAGVDRDREEPQPLPYDLAHDLAAAPP
LLASLPSGAAAATLRRLALEDGRRVCRMILSIAESQNCFKIKEDFFRITDDA

B.tabaci_Corazonin	QTFQYSHGWTS	-amide
B.mori_Corazonin	QTFQYSRGWTN	-amide
N.viridula_Corazonin	QTFQYSRGWTN	-amide
S.invicta_Corazonin	QTFQYSRGWTN	-amide
A.armilla_Corazonin	QTFQYSHGWTN	-amide
C.glomerata_Corazonin	QTFQYSRGWTN	-amide
C.rubecula_Corazonin	QTFQYSRGWTN	-amide
D.alloeum Corazonin	QMVQYSRGWRG	-amide

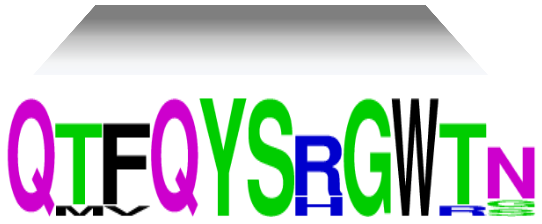

Diuretic hormone 31

>*B. tabaci* _Diuretic hormone XM_019055541.1
MAVTSGLLLGILILLTATQASESVPFSAGRKSYMSEMEEPDTEMMLEMLARLGQSIIRANDLENSKRGLDLGLSRGFSGSQAAKHLM
GLAAANYAGGPGRRRRASNILPNEI-

B.tabaci_Diuretic_hormone_31	GLDLGLSRGFSGSQAAKHLMGLAAANYAGGP	**-amide**
B.mori_Diuretic_hormone_31	AFDLGLGRGYSGALQAKHLMGLAAANFAGGP	**-amide**
D.citri_Diuretic_hormone_31	GLDLGLSRGFSGSQAAKHLMGLAAANYAGGP	**-amide**
N.lugens_Diuretic_hormone_31	GLDLGLSRGFSGSQAAKHLMGLAAANYAAGP	**-amide**
A.armilla_Diuretic_hormone_31	GLDLGLSRGFSGSQAAKHLMGLAAANYAGGP	**-amide**
C.solmsi_Diuretic_hormone_31	GLDLGLSRGFSGSQAAKHLMGLAAANYAGGP	**-amide**
Cviridula Diuretic hormone 31	GLDLGLSRGFSGSQAAKHLMGLAAANYAGGP	**-amide**

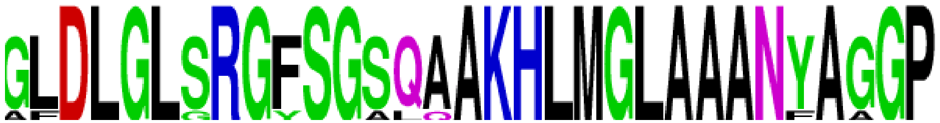

Diuretic hormone 45

> *B. tabaci* _ DH45 XM_019041531.1

---------LKFQMAELLKVVCIGTVMLMLSGGAAGYYRGLEEEVEQIDAVPPDIESSPYLLPKLSHHFAPFDPQPPNGLWKHVS
DPNLYILSERESQDKEGELKSKRNGRNGLTGNGPSLSIVNPLEVLRQRLLLEIARRRMRQSEDQIQANRELLKTIGKRSLAP
ERRRDPGPAPKEHRANQRPDTENHSAHSDRWNRHTYNYAENA

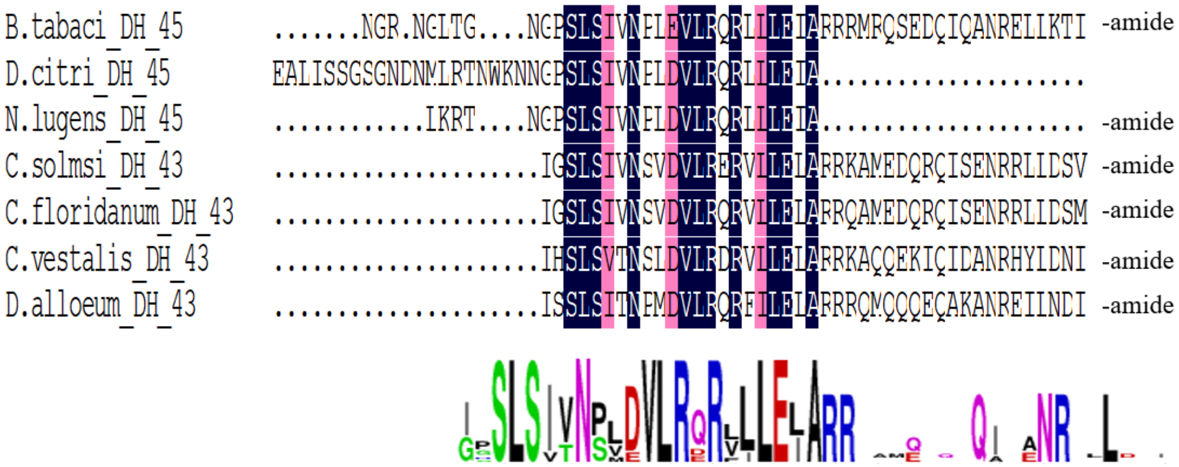

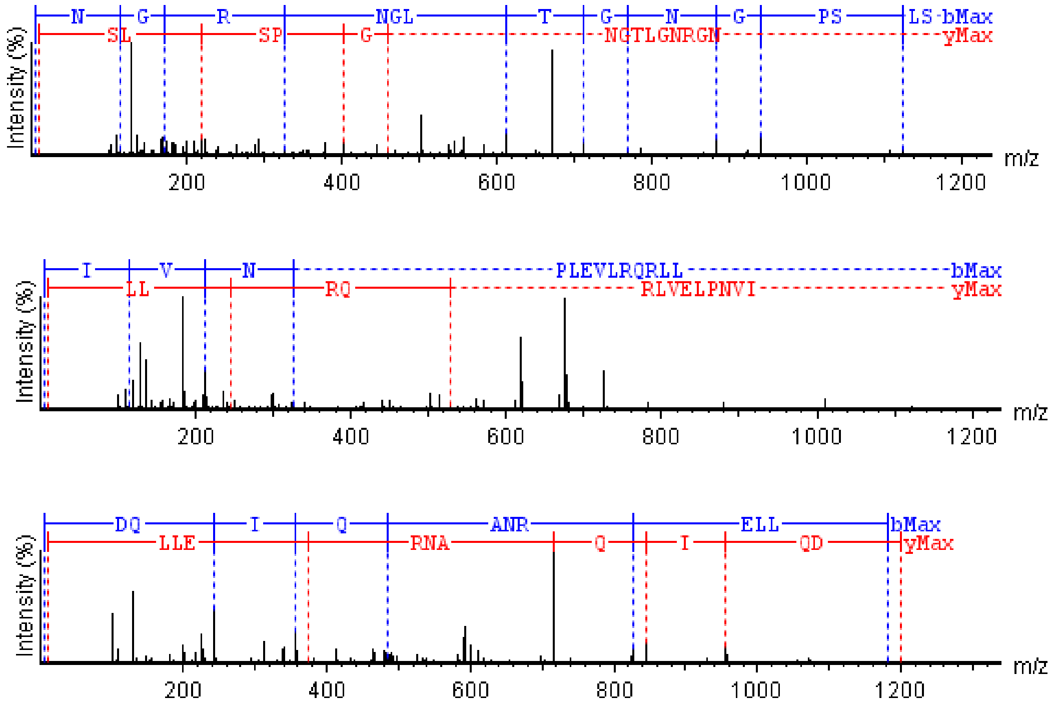

ETH

> *B. tabaci*_ETH XM_019048228.1

MKLSHHRPFVLMIGFTIFGSVLAEPRPVKGDDLSTLSETKDKREISVEAEVGERLRRSDDFFLKASKSVPRIGRGRRGDDFFIKASKNVPRVG
RRNTEFLKASKAIPRIGKKNSDPFLIGVKSVPRIGRSGETDEQSPVHKREDTFLAVNDGGLWGVWPWFRNQDFPGIAPSKRSSYILSSYNDSP
LRDKQTAWSPVDTIAQEVELGPPIYLEEQV-

B.tabaci_ETH_1-1	. . . SDDFFLKASKSVPRI	amide
B.tabaci_ETH_1-2	. . . GDDFFIKASKNVPRV	amide
B.tabaci_ETH_1-3	. . . . NTEFLKASKAI PRI	amide
B.tabaci_ETH_1-4	. . . NSDPFLI GVKSVPRI	amide
A.pisum_ETH	GFAGEEFFLKASKSVPRI	amide
D.melanogaster_ETH_1-1	DDSSPGFFLKI TKNVPRL	amide
D.melanogaster_ETH_1-2	. . . GENFAI KNLKTI PRI	amide
B.dorsalis_ETH_1-1	. NESPGFFLKI TKNVPRL	amide
B.dorsalis_ETH_1-2	. . . SDSYFLKNMKTI PRI	amide
Z._nevadensis_ETH_1-1	DETGTNFFLKSSKSVPRI	amide
Z._nevadensis_ETH_1-2	. . . SEYDFLKASKEI PRI	amide

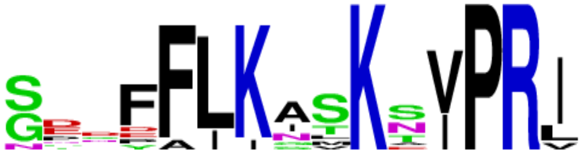

EH

> *B. tabaci*_EH 1 Scaffold2557

MSFLKTTAIMASLGLVLFALLLSEAEANQIGICMRNCILCKKMLGNFFEGHLCADTCLKYKGKMIPDCEDIASISPF
LNKYA

> *B. tabaci*_EH 2 Scaffold2557

---------NCAQCAKALGKYFMPQPCAEYCVSFPFIENKFDCYKLSSVGRYLRRLLVF

```
B._tabaci_EH_1          .......................................NQIGICMRNCILCKKMLGNFFEGHLCADTCLKYKGKMIP...DCEDIASISPF.......
B._tabaci_EH_2          .......................................NCAQCAKALGKYFMPQPCAEYCVSFP..FIENKFDCYKLSSVGRYLRRLLVF
L._migratoria_EH_1      ...............................SAVGVCIRNCAQCKKMFGPYFEGQLCGDACLKFKGKMVP...DCEDAASIAPFLSKLE..
L._migratoria_EH_2      ...............................NAVSVCIRNCAQCKKMYGPYFEGQLCADACLKFGGKMMP...DCEDAASIAPFLNKLE..
Z._nevadensis_EH_1      MEQRKISEAVVLAMSVAFLAATVVVPSGATSYSIGVCIRNCAQCKKMFGPYFEGQLCADACVKFKGKIIP...DCEDLASIAPFLNKFE..
Z._nevadensis_EH_2      ..............................GSNVGVCITNCGQCKQMYGHYFEGQVCVESCLSTNGNLLP...DCNNPNTLRGLLKRLY..
A._pisum_EH_1           .............................DMADVAMCIRNCAQCKKMLGDYFEGPLCADTCVKFKGKMIP...DCENIDSIAPFLNKLE..
A._pisum_EH_2&3         .............................DMADVGLCIRNCAQCKKMLGAYFEGPLCADACVKFKGKMIP...DCENIDSVAPFLNKLE..
```

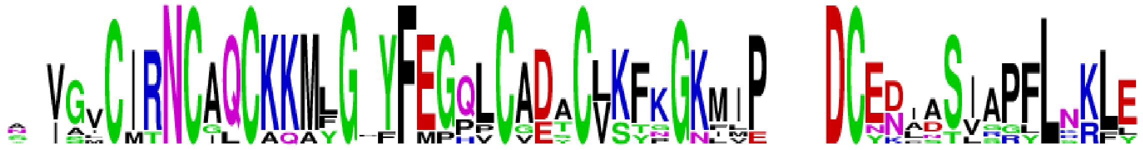

FMRFamide

>*B. tabaci*_FMRFamide XM_019044377.1

MMNLKLCYFSFFVLVYEWISTDLVEAEKVIGLDTGSLDDDDTEVGVVQKRFVQRAIDPLMRRSPLDKNFMRFGKRSEDKGHQDEPELDEIINMTPSDYKLIADDSEPKAKRVKQDF
IRLGRGKQDFIRFGRGKQDFIRFGRGKQDFIRFGRGKQDFIRFGRGDASNLLHYPETNYDLEVAEDDFRNADDLAERDPRAKNSFIRFGRAENFIRFGRNAEVGTPSDNINYRSKRSG
DEFRFARAGDPLPALFVADVNPVEIRRSSGFIRLGRAKATGSKEPEMGDRRKRSLDDVDSSTPEPKVVPKIGFKGLVVVPPGSEGGVEPEPEMDGSENGSRNETEDGYSTEMNDFPV
TEPPEKNFHIPSIRHKRRTYEDYRPRDLAMLNNNVYDALAGSADCSPMVRLGPSLSKDYVVKVG-

B.tabaci_FMRFamide_1-1	. . . S P L DKNFMRF **-amide**
B.tabaci_FMRFamide_1-2	. . . . . VKQDFIRL **-amide**
B.tabaci_FMRFamide_1-3	. . . . QDFIRF **-amide**
B.tabaci_FMRFamide_1-4	. . . . QDFIRF **-amide**
B.tabaci_FMRFamide_1-5	. . . . QDFIRF **-amide**
B.tabaci_FMRFamide_1-6	. . . . QDFIRF **-amide**
B.tabaci_FMRFamide_1-7	. . . . . AKNS FIRF **-amide**
B.tabaci_FMRFamide_1-8	. . . . AE NFIRF **-amide**
B.tabaci_FMRFamide_1-9	. . . . S S GFIRL **-amide**
A.pisum_FMRFamide_1-1	. . . S AMDKNFMRF **-amide**
A.pisum_FMRFamide_1-2	. . . . . VDS NFIRF **-amide**
A.pisum_FMRFamide_1-3	. . . . DS NFIRF **-amide**
A.pisum_FMRFamide_1-4	HYDVDVDGL EVRF **-amide**
B.mori_FMRFamide_1-1	. . . S AI DRS MIRF **-amide**
B.mori_FMRFamide_1-2	. . . . S AS FVRF **-amide**
B.mori_FMRFamide_1-3	. . . . DP S FIRF **-amide**
B.mori_FMRFamide_1-4	. . . HRARNHFIRL **-amide**

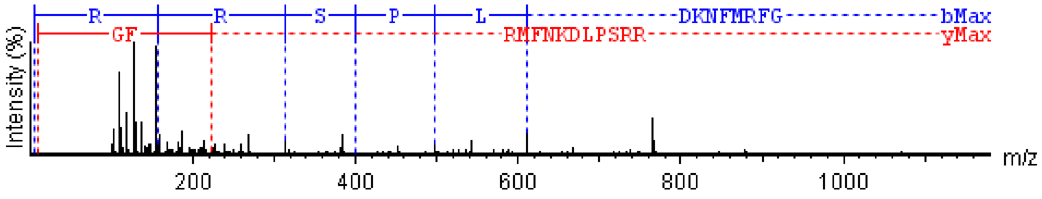

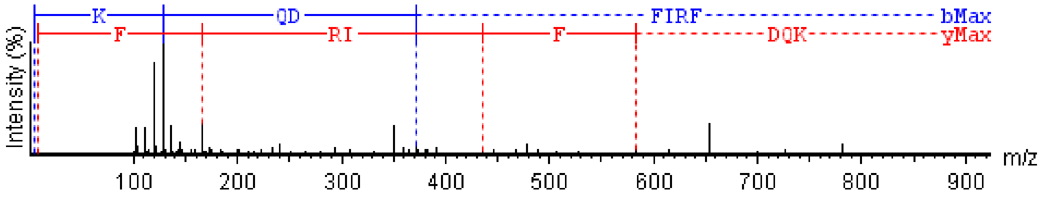

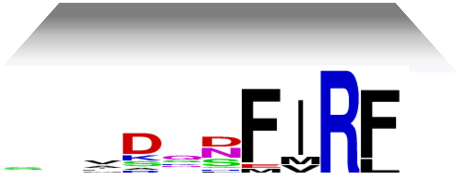

Insulin-related peptide

> *B. tabaci* _IRP 1 KF055836.1

MRNQKPMLLLIVTSFVTLILIDGRIARGQHQFARDARSGAARYCGKRMAEMLMMVCRGALSKRTLQKLDNEYQSLGDETDTRLNLRELDYPGAL
FGQFYPNEFQIFNKAKALSTINDENFRRRRRGIVEECCYNACSKDDLRLYCAHFK-

> *B. tabaci* _IRP 2 XM_019053653.1

-------KMSSPKSTVALIVTSAVTLILINGHFARGQPHFTRAARSDLPHLCGKRLSDALNIICRGGGPPFYFKRASQRFEDEYHDASDLSMVSRDLDYPG
ALSSQLSSNEFPVVSETEMLSAATDDNFRRPRRQIVEECCLKPCTEDHLRSYCGTFH-

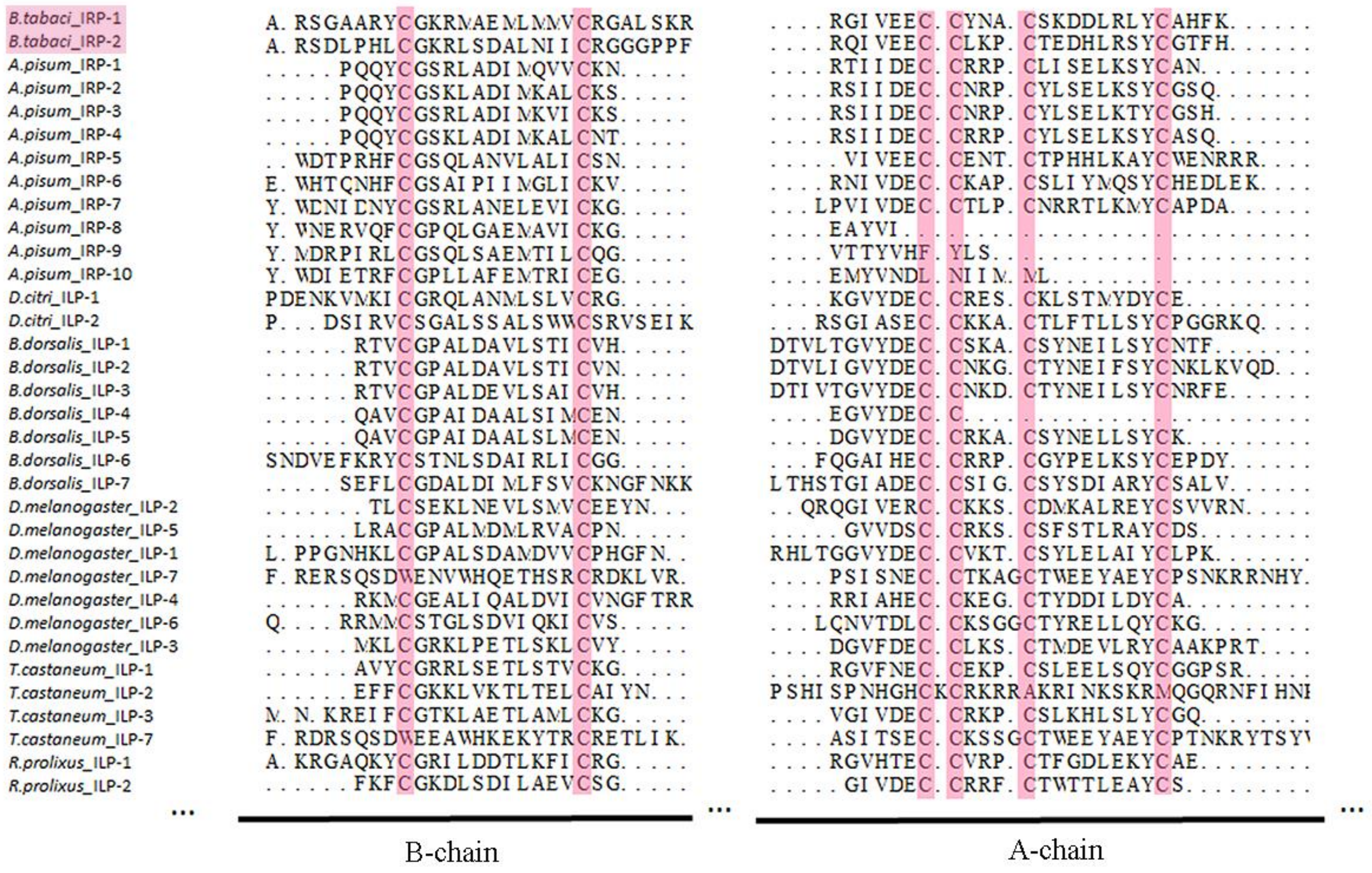

Ion transport peptide

> *B. tabaci* _ITP short isoform XM_019044831.1

MHQSTQVLLLVSWCLMSNILAGPTSRALLGHSFNKRSFFNLECKGVYDKSIFAKLDRICEDCYNLFREPQLHSQCRSGCFTSEFFEACLEALLLKEEEMSIYRKVGYLREKRN-

> *B. tabaci* _ITP long isoform XM_019044830.1

MHQSTQVLLLVSWCLMSNILAGPTSRALLGHSFNKRSFFNLECKGVYDKSIFAKLDRICEDCYNLFREPQLHSQCRKNCFTTDYFKGCLEVLLLEDEMEKIQTWIKQLHGAESGV-

```
B.tabaci_ITP_1       SFFNLECKGVYDKSIFAKLDRICEDCYNLFREPQLHSQCRSGCFTSEFFEACLEALLLKEEEMSIYRKVGYLRE.....
B.tabaci_ITP_2       SFFNLECKGVYDKSIFAKLDRICEDCYNLFREPQLHSQCRKNCFTTDYFKGCLEVLLLEDEMEKIQTWIKQLHGAESGV
B.mori_ITP           SFFTLECKGVFDAAIFARLDRICDDCFNLFREPQLYTLCRAECFTTPYFKGCMESLYLYDEKEQIDQMIDFV.......
N.viridula_ITP       SFFDLSCKGVYDKSIFARLDRICEDCYNLFREPQLHSLCRKNCFTTDYFKGCLDVLLLQDEMENIQTWIKQLHGAEPEV
R.prolixus_ITP_1     SFFDLQCKGVYDKSIFARLDRICEDCYNLFREPQLHSLCRSDCFASKYFAGCLEALLLREEENKFFQMVEFLG......
R.prolixus_ITP_2     SFFDLQCKGVYDKSIFARLDRICEDCYNLFREPQLHSLCRKNCFTTDYFKGCLEVLLLQDEMENIQTWIKQLHGAEPEV
C.glomerata_ITP      SYFDLGCKGVYDKSIFARLDRVCEDCFNLFREPQLHTLCRQDCFRTNYFKSCTQVLTLEDEEEKFQEMIEYL.......
C.rubecula_ITP       SYFDLGCKGVYDKSIFARLDRVCEDCFNLFREPQLHTLCRQDCFRTNYFKSCTQVLTLEDEEEKFQEMIEYL.......
F.arisanus_ITP       SFMELQCKGIYDKSIFARLDRICEDCYNLFREPQLHTLCRQDCFQTKYFTSCIQALLLEDEKEKFLEMVEYL.......
```

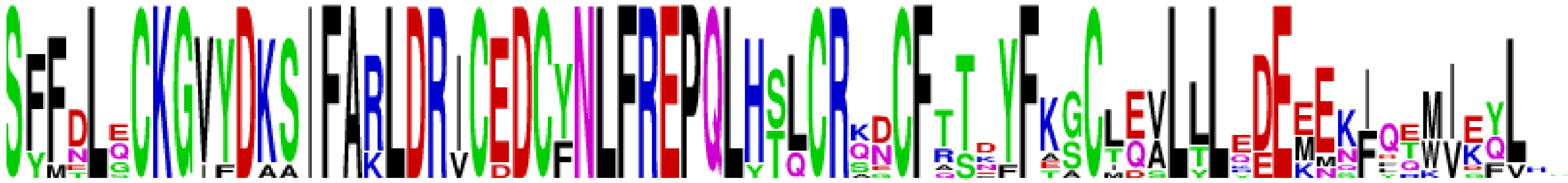

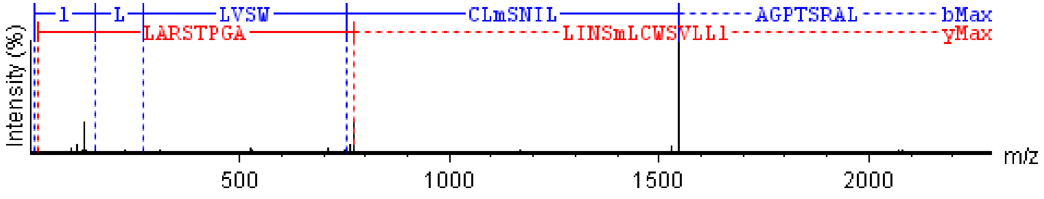

Leucokinin

> *B. tabaci* _Leucokinin XM_019054656.1

MTLAMSGSLHKFRMLIAWFPILVLPVLASYARAKPEEAEEVSQTSATRDGLSDASLYDQSEPATSPACDTSADSDIESVLQEQLPPENDSWPSDEDLSDYDPDDWADGQHRREKR
QAEGQNNDPLDFLEHLDPKRAKPAFNSWGGKRTSSFHSWGGKRSSEDSIDALEETKRNDPFIILKFADSLYNTNGGFKRRPSFNSWGGKRAGTAAFNSWSGKRAGAAAFNSWGG
KRGPNFNSWGGKRAPPKPAFNSWGGKRGPAFNSWGGKRGPAFNSWGGKRANFNSWGGKRGPAFNSWGGKRSPAFKSWGGKRANFNSWGGKRANFNSWGGKRSPAFNSWG
GKRAPAFNSWGGKRAASGSTYNAQGGKRGPAFYSWGGKRSSNENYNLGQENKETGILELVKEPGNLDQPISLNKREATKSTAPEAGPSDKNSDNTHTLVKREVDSREGNLDPSI
AAVEKLYRDFKEREKKCAKSKSIVHGERVKHLLNYILNLRLSDREPEEKKSVIHKMLLRAADYSPDSSTFTQSRRGAEFHSWGGKRSEVNSAWYGHD-

Bemisia_tabaci1	. . AKPAFNSWG	-amide
Bemisia_tabaci2	. . . TSSFHSWG	-amide
Bemisia_tabaci3	. . . RPSFNSWG	-amide
Bemisia_tabaci4	. AGTAAFNSWS	-amide
Bemisia_tabaci5	. AGAAAFNSWG	-amide
Bemisia_tabaci6	. . . GPNFNSWG	-amide
Bemisia_tabaci7	APPKPAFNSWG	-amide
Bemisia_tabaci8	. . . GPAFNSWG	-amide
Bemisia_tabaci9	. . . GPAFNSWG	-amide
Bemisia_tabaci10	. . . . ANFNSWG	-amide
Bemisia_tabaci11	. . . GPAFNSWG	-amide
Bemisia_tabaci12	. . . SPAFKSWG	-amide
Bemisia_tabaci13	. . . . ANFNSWG	-amide
Bemisia_tabaci14	. . . . ANFNSWG	-amide
Bemisia_tabaci15	. . . SPAFNSWG	-amide
Bemisia_tabaci16	. . . APAFNSWG	-amide
Bemisia_tabaci17	AASGSTYNAQG	-amide
Bemisia_tabaci18	. . . GPAFYSWG	-amide
Bemisia_tabaci19	. . . GAEFHSWG	-amide

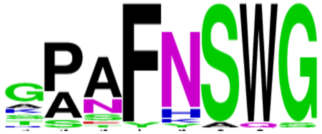

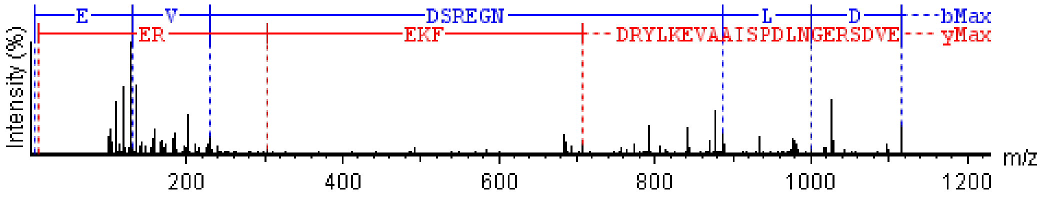

>*A. pisum* Leucokinin X4 - - - - - - - - - - - - - -

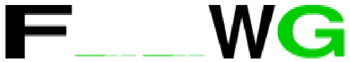

>*O. abietinus* Leucokinin X7 - - - - - - - - - - - - - -

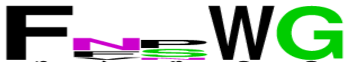

>*R. prolixus* Leucokinin X11 - - - - - - - - - - - - - -

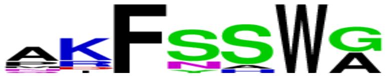

Myossuppressin

> *B. tabaci* _ Myosuppressin XM_019053051.1

MRCVLVIFSFLLAAVQLMGRVTEAMPPTLCISNPIEEVPLRVRKVCAALSSIYELAGAMESYLDTEGHPQNDAVFS
HHRQILENSLRKRVLGDDEPPQQLGAKRPDHMFMRFGRR

B.tabaci___Myosuppressin_	. . P DHMF MRF-amide
R.prolixus_Myosuppressin	QDI DHVF MRF-amide
A.pisum__Myosuppressin	QDL DHVF LRF-amide
N.viridula__Myosuppressin	QDL DHVF LRF-amide
N.lugens__Myosuppressin	QDVDHVF LRF-amide
A.armilla_Myosuppressin	. DVDHVF LRF-amide
C.floridanum_Myosuppressin	. DVDHVF LRF-amide
C.rubecula_Myosuppressin	. DVDHVF LRF-amide

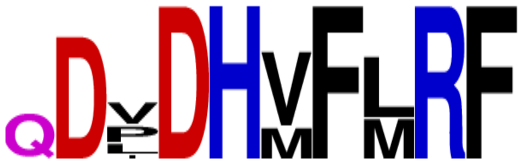

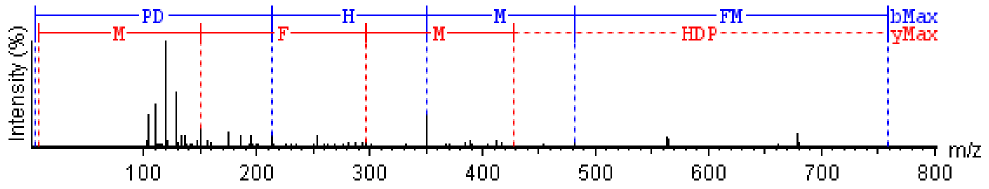

Natalisin

>*B. tabaci*_Natalisin XM_019054608.1

MCSLLEPSASMAPVLLVVFLATAFHQVCMTSGDARVGRSDVREVLRGQIDPSFWPTRGRRGGSSEESEPPFWANRGREFQSEEEQPPFWGNRGRTLDQDPDTHHHSRPPVEALLAVDRDPEALIPHIQKWFNVRKRQQRLASCCDTPFSANYSMYVEEPQHILLERRGGSEEKDDQFWVSRGRREATEKRESNLERLNREFSPLYEDAEEGPSVQKRSEPPPAPKVEDPNAEDAKFWKSLASTIGAKRARPSNASDAEPQQ

```
B._tabaci1-1         ........................SDVREVLRGQI DPSFWPTR -amide
B._tabaci1-2         ........................SSEE......SEPPFWANR -amide
B._tabaci1-3         ......................EFQSEE......EQPPFWGNR -amide
B._tabaci1-4         ..........................GG...SEEKDDQFWVSR -amide
Z._nevadensis1-1     .....................VTRSDVRATLGNKPEPGFWPAR -amide
Z._nevadensis1-2     ...................DEPNVSPDG...TND.DDPFWVAR -amide
Z._nevadensis1-3     ......................S.DHDS...LESAEDPFWPTR -amide
Z._nevadensis1-4     ......................G.LLES...LS.AGEPFWAAR -amide
Z._nevadensis1-5     ......................GSLLDS...LS.AEVPFWAAR -amide
D._citri1-1          ......................AKSSEEE....VDEPPFWAQR -amide
D._citri1-2          ........................DNSD...DDHNYSNFWASR -amide
C._glomerata1-1      MVEHPFYVDEPGWVMLSESNTNLMSNDNKLI KADQDPFYLAR -amide
C._glomerata1-2      .........................SDI...SDLLHEPFFISR -amide
```

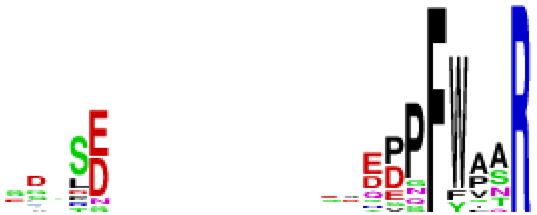

Neuroparsin

> *B. tabaci* _ Neuroparsin XM_019057891.1

MQLTFGVLVTSFLVTLLHPSLRTVVGGSLKLKLCLPCIGEECNLLPEEECLYGLVKNHCGYWECGKGPGQICGGPSKIFGECGDGMHCKCDRCVGCSSKTLDCDLNTSIC-

```
B.tabaci__Neuroparsin         .......GSLKLKL.....CLPCIGEECNLLPEEECLYGLVKN.HCGYWECGKGPGQICG      47
R.prolixus_Neuroparsin        ..........VFYG.....CVPCIGDECNLNPGN.CPYGIVRD.PCGRLVCAAGPGERCG      43
C.floridanum_Neuroparsin      LSIGRPYPTIIERSEQRSECESCAGE.CDK.....CKYGHAFSRFCGVEECLKGPGESCG      54
D.alloeum_Neuroparsin         ......HPALRIRDVERAGCRPCGDE.CEA.....CEYGVAYSPFCGVLECRKGPDEHCG      48
F.arisanus_Neuroparsin        ......HPFIRLSDLEKMSCRPCGED.CDG.....CEYGVVFSPLCRALECRKGPDEHCS      48
L.boulardi_Neuroparsin        ......HPMIKDREEERLLCETCGDD.CDK.....CKFGVTFSSLCEVWQCQRGPGEICG      48
C.solmsi_Neuroparsin          ......HPTIIERGEQRSECEGCADE.CEK.....CKYGFAFSNWCGLKECLKGPGEMCG      48

B.tabaci__Neuroparsin         GPSKIFGECGDGMHCKCDRCVGCSSK.......TLDCDLNTSIC...............      84
R.prolixus_Neuroparsin        GRDFHLGKCGEGLSCKCGKCRGCSIKQIMN..GRIDCDTTNPMCQ..............      86
C.floridanum_Neuroparsin      GMRNKYGECGDGMYCRCNKCNGCSSDTLEC..HSVVCPIYETRSGHPVAHTNSLMLLDK.     111
D.alloeum_Neuroparsin         ARG....TCGDGMYCLCERCFGCSTDNLKCSDLTPTCLPRETRYRNLQRSRQFTMV....     100
F.arisanus_Neuroparsin        TEN....SCAEGLSCICEKCIGCSIEKLKCSDSILTCLPRQGRYKHLERSRQFSMV....     100
L.boulardi_Neuroparsin        GRGDKYGVCGDGLSCHCNKCVGCSIDSLDC..YVNNCLPRTEIQLSHPNRFNELI.LGK.     104
C.solmsi_Neuroparsin          GVRNKYGACGEGMYCRCNKCTGCSIDTLEC..FSGFCPIYEQMQLRHPEHMQGLQ.LDK.     104
```

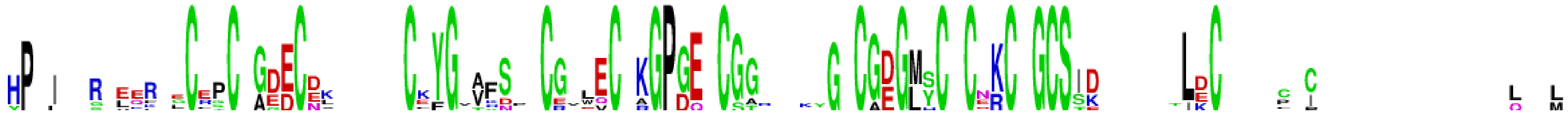

Neuropeptide F

> *B. tabaci* _Neuropeptide F XM_019051892.1

MIGSLNFAVASLCTLIWISLAMPSSCEPLPMGNDHAPRTGRNKPFGSPDELRSYLDELGQYFAVVGRPRFGKRTLLPLLAGPSMKD
PSNHRIGLGAAFPPLDSSDVYRLVYSPENDVS-

NPF 1

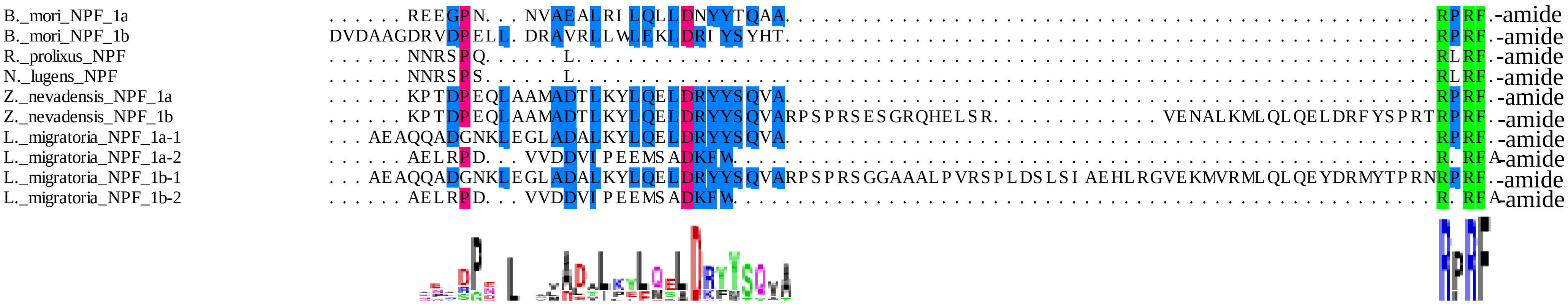

NPF 2

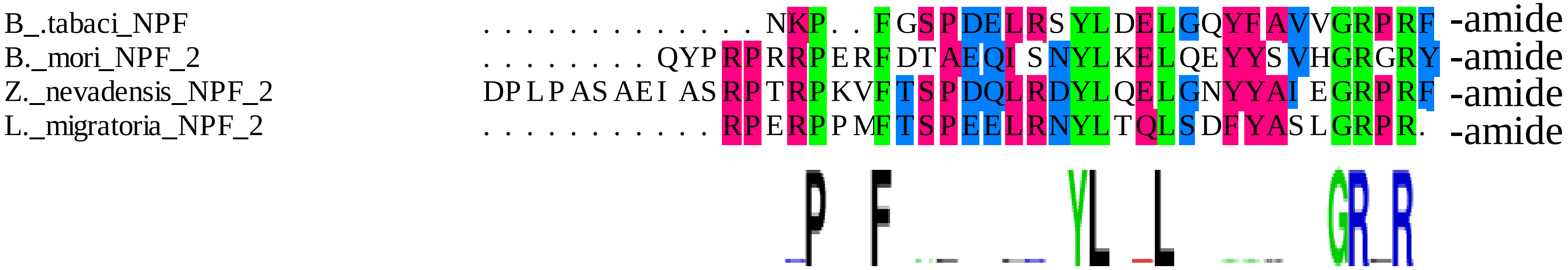

Orcokinin-A

> *B. tabaci* _Orcokinin-A XM_019055006.1

MQSPLAQPRIWILVLQFVVLFAPSHLYPVEDQESEAVYRSSNGPRVPHLGDALLRDLDLLRNRASYYAAQSRQYGPLVSRWEKR
FDSLDGSTLGEQKRNFDEIDRSGFNKFTHKRNFDEIDRSGFDSFVKRQRNFDEIDRAGFVGFNKRSVPDAAYQKPPSKAAN-

B._tabaci_Orcokinin-A_1-1	. . NFDEI DRSGFNKFTH
B._tabaci_Orcokinin-A_1-2	. . NFDEI DRSGFDSFV.
B._tabaci_Orcokinin-A_1-3	QRNFDEI DRAGFVGFN.
Z._nevadensis_Orcokinin-A_1-1	. SGLDSLSGVTFGWN. .
Z._nevadensis_Orcokinin-A_1-2	. . . LDSLRGI TFGNQ. .
Z._nevadensis_Orcokinin-A_1-3	. . NFDEI DRSGFNSFV.
Z._nevadensis_Orcokinin-A_1-4	. . NFDEI DRSGFDGFV.
Z._nevadensis_Orcokinin-A_1-5	. . NFDEI DRVGFGSFV.
L._migratoria_Orcokinin-A_1-1	. DEPNDVEEGLV. . . . .
L._migratoria_Orcokinin-A_1-2	. . HLDNI GGGHLL. . . .
L._migratoria_Orcokinin-A_1-3	. . NLDGLGGGHLLRQT.
L._migratoria_Orcokinin-A_1-4	. SGLDSLSGATFGEQ. .
L._migratoria_Orcokinin-A_1-5	. . . LDSLSGI TFGNQ. .
L._migratoria_Orcokinin-A_1-6	. . NFDEI DRSGFNSFI .
L._migratoria_Orcokinin-A_1-7	. . NFDEI DRSGFDRFV.
L._migratoria_Orcokinin-A_1-8	. . NFDEI DRSGFSGFV.

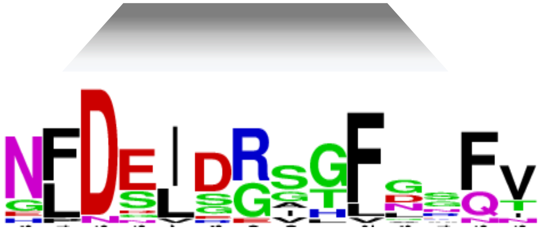

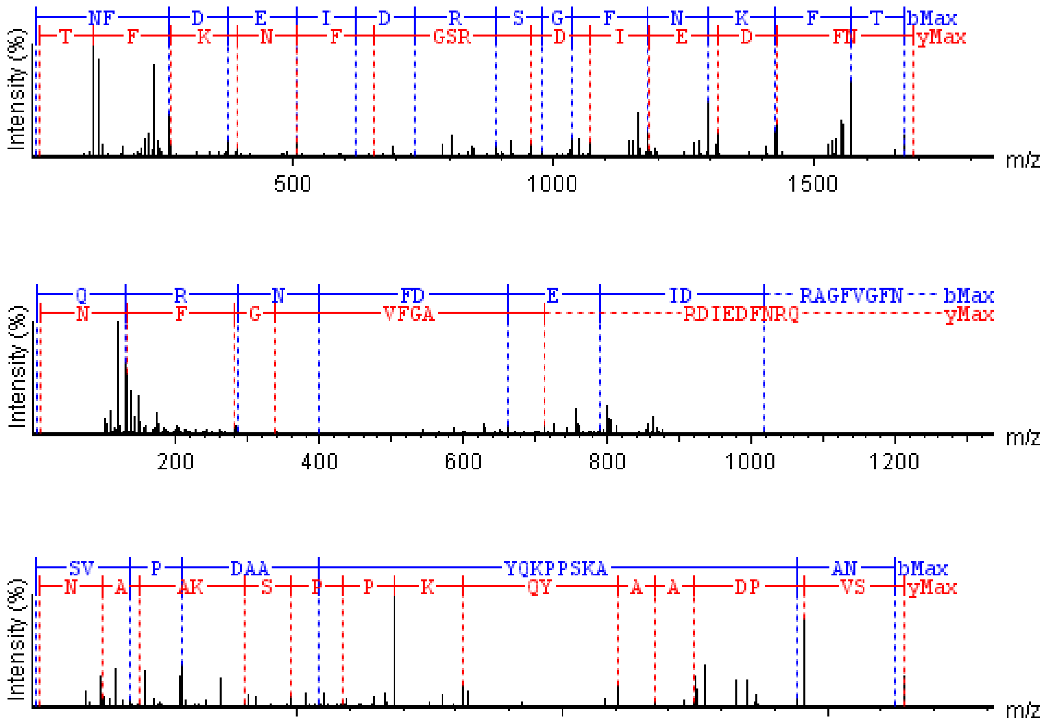

Orcokinin-B

> *B. tabaci* _Orcokinin-B XM_019055005.1

MQSPLAQPRIWILVLQFVVLFAPSHLYPVEDQESEAVYRSSNGPRVPHLERNRYERPTQLYNDPLLDQALNEVEYTRHRNRIEHGSPEAVPDLESGFPPRCMGLTCLGEETEMGD
GFRFSHARGKRSEVAIGGTGTLDKLGGANLIRKRFFQPEVRNREPGLDATWHLGRNNAEDRDSIAVDFSKRGLFDSFFSTPRPSFDSRVAKEHPFLATSQSGLFKDYNSLLQITPA
PPTQSSFSLGNLFWGGQTSKRTGHLDRLGGANLVKRRDSDDGYFESVGGGSQENGVQGPRVFLGDDKLSLAKSRIHNKPSEDGEVLFDFLKGTVVGQDGDSVDFGFIHGEPYL
KSSRVDSRISGDNPFQGFLSNDYIENLEKSNLQRRGYLDSLGGSNLQKRRVLDQLGGQNLQKRLLDSLGGQNLQKREYLDALGGQNLQKRYRERQSLRKRYLDSLGGQNLQK
KSHLDSLGGQNLQKRNFFDFLGDSSSDEGLAGVVNDTNGPGNRVDEIIYVSHSVKRNAGNETTPEGMGAVAKVEIIGAPQQGENKELFRVNLSEGNNPTKFTETSSSQNLDPLG
GANLIRNLDALDG

B._tabaci_1-1	. . . . SEVAIGGTGTLDKLGGANLI
B._tabaci_1-2	. . . . TGHLDRLGGANLV
B._tabaci_1-3	. . . . . . ISGDNP . . FQGFLSNDYIENLEKSNLQ
B._tabaci_1-4	. . . . GYLDSLGGSNLQ
B._tabaci_1-5	. . . . RVLDQLGGQNLQ
B._tabaci_1-6	. . . . LLDSLGGQNLQ
B._tabaci_1-7	. . . . EYLDALGGQNLQ
B._tabaci_1-8	. . . . YRERQSLR
B._tabaci_1-9	. . . . YLDSLGGQNLQ
B._tabaci_1-10	. . . . SHLDSLGGQNLQ
B._tabaci_1-11	NAGNETTPEGMGAVAKVEIIGAPQQGENKELF
B._tabaci_1-12	. . VNLSEGNNPTKFTETSSSQNLDPLGGANLI

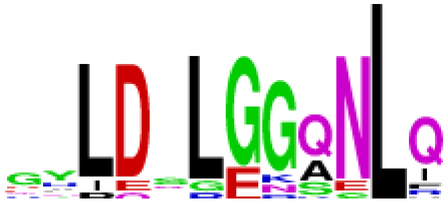

*L. migraroria*_Orcokinin-B X31 - - - - - - - - - - -

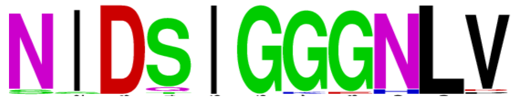

*Z. nevadensis*_Orcokinin-B X18 - - - - - - - - - - -

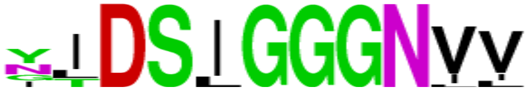

*B. mori*_Orcokinin-B X7 - - - - - - - - - - -

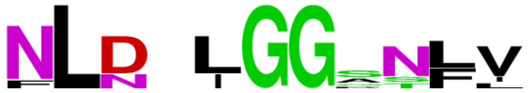

*T. castaneum*_Orcokinin-B X5 - - - - - - - - - - -

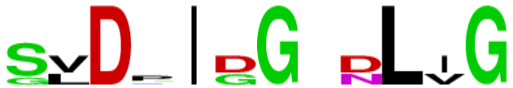

PBAN

> *B. tabaci* _ PBAN XM_019040288.1

--------DKVFCFRNFEMGYLIVAVITSALISFCQGIQQMDPRAISRSDILEEAMLLGLEVGASDGVDSGRKRSGKASFNRADLWFGPRLGRKKRNSEE
ELGPEIPDKDEESILEFIKSSSPWVLIPLKEKANTRSMNYTPRLGRSSKEEEEDFIPEITRSTPFVPRLGKRRNNQIFSPRLGRSDLYYSPRS

B.tabaci_PBAN_1-1	.......................RSGKAS.........FNRADLWFGPRL	-amide
B.tabaci_PBAN_1-2	RNSEEELGPEIPDKDEESILEFIKSSSPWVLIPLKEKANTRSMNYTPRL	-amide
B.tabaci_PBAN_1-3	..........SSKEEEE...DFIPEIT.............RSTPFVPRL	-amide
B.tabaci_PBAN_1-4	....NNQIFSPRL	-amide
R.prolixus_PBAN_1-1	....NTVNFSPRL	-amide
R.prolixus_PBAN_1-2	..............DEE...VVFTETS.............RSPPFAPRL	-amide
H.zea_PBAN_1-1	..LFVFLAVFTTSSVLGNNNDVKDGAASG.....AHSDRLGLWFGPRL	-amide
H.zea_PBAN_1-2	............S.......LAYDDKS............FENVEFTPRL	-amide
H.zea_PBAN_1-3	......LSDDMPATPADQ..EMYRQDP........EQIDSRTKYFSPRL	-amide
H.zea_PBAN_1-4	....TMNFSPRL	-amide

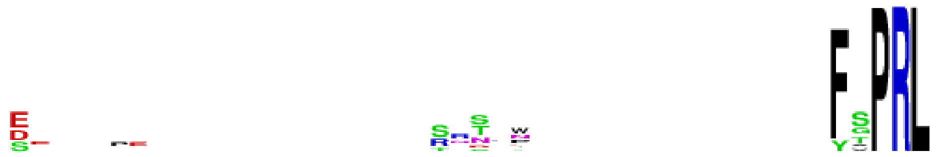

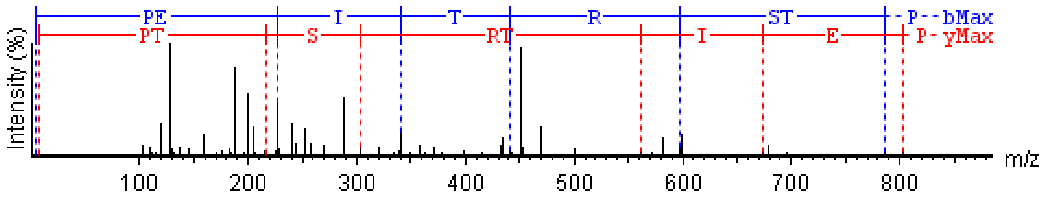

Proctolin

>*B. tabaci_* Proctolin XM_019061130.1

MLRVTLICSVIFFLLVSTKIPVDARYLPTRSMDDRLNRLRELLRDLSFVDRNDRPHSGSNPELGPETDASSSQVDPLFYPFD
SQRLPLPRRFTGTSTN-

B.tabaci_Proctolin	RYLPT
A.pisum_Proctolin	RYLPT
D.citri_Proctolin	RYLPT
N.lugens_Proctolin	RYLPT
L.migratoria_Proctolin	RYLPT
Z.nevadensis_Proctolin	RYLPT

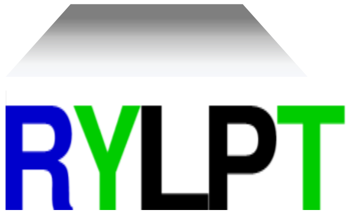

RYamide

>B. tabaci_ Ryamide XM_019048381.1

---------IRTEPKSALMHNLWNRFTTCRLLELELPELLAERGMNPVTSAHVRERWRSPTSGVMSSTALRFSRLTAHKVWNKTGPGFWGWLGWGQERMPGG
AEGGRCAERTSRRFAEEKPPGKASTLRRSSNSALSSVAVQKALVKGFATCPLGSPARSKMLGATVIVESCICLLIFVSTSNSQEFFSTGGRYGKRIASEESFD
TGRPSDTSVLERRSLSITPDNSPLLEVSGRNDRFFMGSRYGKRALDLLKSDFRDVTGARITCVYIGFEDLFRCSNGKDRKFHEVESLQREENK

B._tabaci__RYamide1-1	SKMLGATVIVESCICLLIFVSTSNSQEFFSTGGRY	-amide
B._tabaci__RYamide1-2	.........................NDRFFMGSRY	-amide
Z._nevadensis_Ryamide1-1	.........................AQFYTSG.RY	-amide
Z._nevadensis_Ryamide1-2	....................DLAQR.SMFWSGSRY	-amide
Z._nevadensis_Ryamide1-3	............................FFIGSRY	-amide
C._glomerata_RYamide1-1	.........................PNFYTHG.RY	-amide
C._glomerata_RYamide1-2	..................EETPPTGEVNFNFGSRY	-amide
C._glomerata_RYamide1-3	.....GEN.IEPKATNDKSNELMARVDRFYLGSRY	-amide
L._boulardi_RYamide1-1	.........................NNFYTSG.RY	-amide
L._boulardi_RYamide1-2	..................EESAAPVGEVFLAGSRY	-amide
L._boulardi_RYamide1-3	....SGMMGMKNSRTGPKVSDMAPRVDRFYTGSRY	-amide

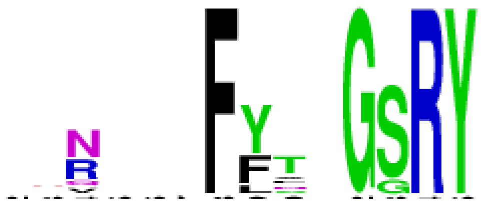

SIFamide

>*B. tabaci_* SIFamide XM_019048993.1

MKRGSGFYFRAVLVLLFLMHISLTIARKPPLNGSIFGKRVSTIEYDVGKTLSAVCEVAVEACSSWFPEEENK-

B.tabaci_SIFamide	..RKPPLNGSIF	-amide
A.pisum_SIFamide	.FRKPPFNGSIF	-amide
B.mori_SIFamide	..RKPPFNGSIF	-amide
D.citri_SIFamide	..RKPPFNGSIF	-amide
N.viridula_SIFamide	..KKPPFNGSIF	-amide
R.prolixus_SIFamide	..KKPPFNGSIF	-amide
T.castaneum_SIFamide	..RKPPFNGSIF	-amide
F.arisanus_SIFamide	AYRKPPFNGSIF	-amide
M.demolitor_SIFamide	AYKKPPFNGSIF	-amide

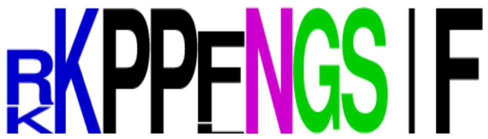

Short Neuropeptide F

>*B. tabaci*_short Neuropeptide F XM_019052600.1

MKYSQIAKLWSVVFCIVLLLEDLVNATPYYSDYDAARDLYELLMQKEALNELAENHRMARKSARTPSLRLRFGRR
SDPNMSSFQQSPTLRLRFGKRSEAVPKDQIDGFDQHFLSEVVKET-

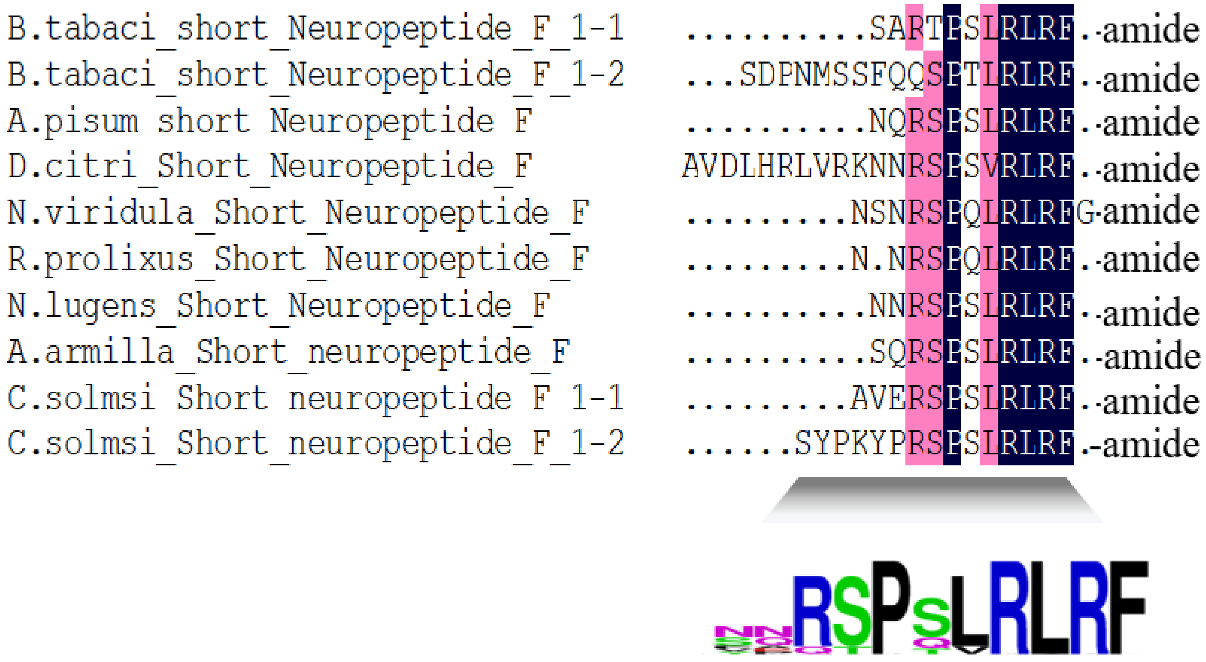

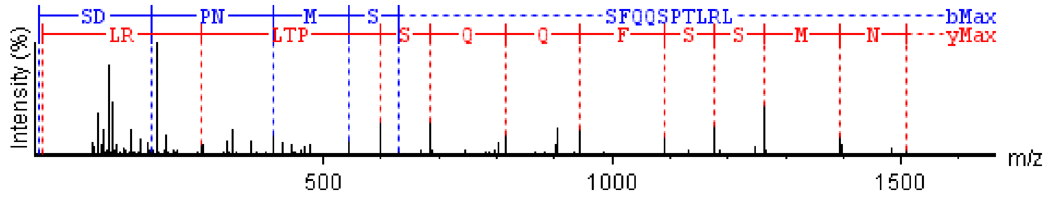

Tachykinin

> *B. tabaci*_Tachykinin XM_019045753.1

MYSLLLLSLFTLLGLGPAPSCGAIEKRSSFDYELQDKKEGPLGGGGGGGSGELVHEGDVYDYAGLEKRAPMGFHGMRGKKKTNNGLMGFV
GTRGKKFDYSDADFQLLDAGIAPYLVGEKRGPSQAFFGMRGKKVPSGSNFFGVRGKKAPSNANFFGVRGKKAPSGFIGMRGKKYSEDDRL
EELIDALKDYTLDSSREKRDIDSALSFQDFAAVRT-

B.tabaci_Tachykinin_1-1	...APMGFHGMR	-amide
B.tabaci_Tachykinin_1-2	TNNGLMGFVGTR	-amide
B.tabaci_Tachykinin_1-3	.GPSQA.FFGMR	-amide
B.tabaci_Tachykinin_1-4	.VPSGSNFFGVR	-amide
B.tabaci_Tachykinin_1-5	.APSNANFFGVR	-amide
B.tabaci_Tachykinin_1-6	...APSGFIGMR	-amide
L.migratoria_Tachykinin_1-1	..APSLGFHGVR	-amide
L.migratoria_Tachykinin_1-2	..APSLGFHGVR	-amide
L.migratoria_Tachykinin_1-3	..APMRGFQSVR	-amide
L.migratoria_Tachykinin_1-4	...ALKGFFGTR	-amide
L.migratoria_Tachykinin_1-5	..APQAGFYGVR	-amide
L.migratoria_Tachykinin_1-6	..GP.SGFYGVR	-amide
L.migratoria_Tachykinin_1-7	APL.SGFYGVR	-amide
L.migratoria_Tachykinin_1-8	AP.SAGFHGVR	-amide
L.migratoria_Tachykinin_1-9	AP..VGFYGTR	-amide
Z.nevadensis__Tachykinin_1-1	AP..SGFLGVR	-amide
Z.nevadensis__Tachykinin_1-2	AP.AMGFQGVR	-amide
Z.nevadensis__Tachykinin_1-3	GP.SMGFHGMR	-amide
Z.nevadensis__Tachykinin_1-4	AP.SMGFMGMR	-amide
Z.nevadensis__Tachykinin_1-5	AP.SLGFQGMR	-amide
Z.nevadensis__Tachykinin_1-6	APAANGFFGTR	-amide
Z.nevadensis__Tachykinin_1-7	VP.ANGFFGTR	-amide
Z.nevadensis__Tachykinin_1-8	GP.SAGFFAMR	-amide
Z.nevadensis__Tachykinin_1-9	AP..IGFLGTR	-amide

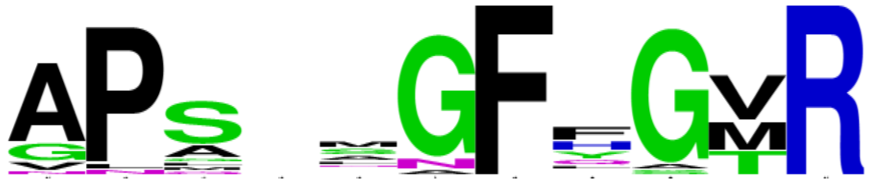

Supplement: Supplementary file 3 — Supplementary data S3 Predicted structures of neuropeptide precursors of Bemisia tabaci. Predicted signalpeptides (highlighted in yellow), cleavage signals (red), putative bioactive mature peptides (light blue), amidation signals (pink), N‐terminal Glutamate (Q) to Pyroglutamate (pQ) conversion (green) and cysteine residues (deep yellow) are indicated. [file INS-28-35-s003.pdf]
