# Supplementary material for: Genome‐wide identification of neuropeptides and their receptor genes in Bemisia tabaci and their transcript accumulation change in response to temperature stresses
Source: Insect Sci. 2020 May 25;28(1):35–46. doi: 10.1111/1744-7917.12751 (PMC7818427; doi:10.1111/1744-7917.12751)
Supplement: Supplementary file 4 — Supplementary data S4 The expression of neuropeptide precursor and receptor genes under temperature treatment in Bemisia tabaci. The quantitative real‐time polymerase chain reaction analysis results of neuropeptides in B. tabaci. Data are presented as means ± SE based on three independent experiments (*P < 0.05, **P < 0.01, ***P < 0.001, independent samples t‐test). [file INS-28-35-s004.pdf]

Supplementary data S4.

The expression of neuropeptide precursor and receptor genes under temperature treatment in *B. tabaci*. The qRT-PCR analysis results of neuropeptides in *B. tabaci*. Data are presented as means ± SE based on three independent experiments (* $P < 0.05$, **$P < 0.01$, *** $P < 0.001$, Independent Samples t-test).

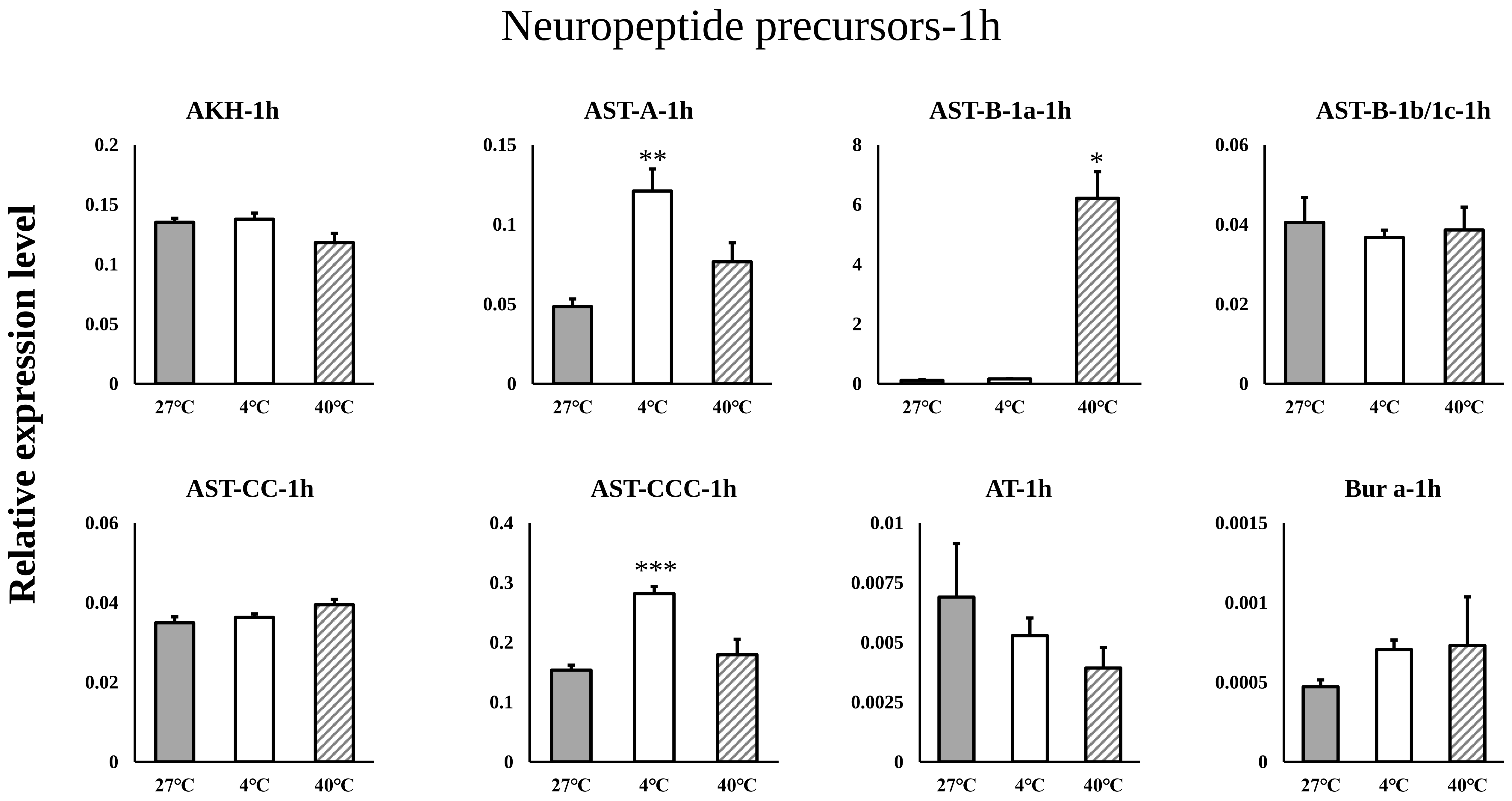
Neuropeptide precursors-1h
Relative expression level
AKH-1h
0.2
0.15
0.1
0.05
0
27℃
4℃
40℃
AST-A-1h
0.15
0.1
0.05
0
**
AST-B-1a-1h
8
6
4
2
0
*
AST-B-1b/1c-1h
0.06
0.04
0.02
0
AST-CC-1h
0.06
0.04
0.02
0
AST-CCC-1h
0.4
0.3
0.2
0.1
0
***
AT-1h
0.01
0.0075
0.005
0.0025
0
Bur a-1h
0.0015
0.001
0.0005
0

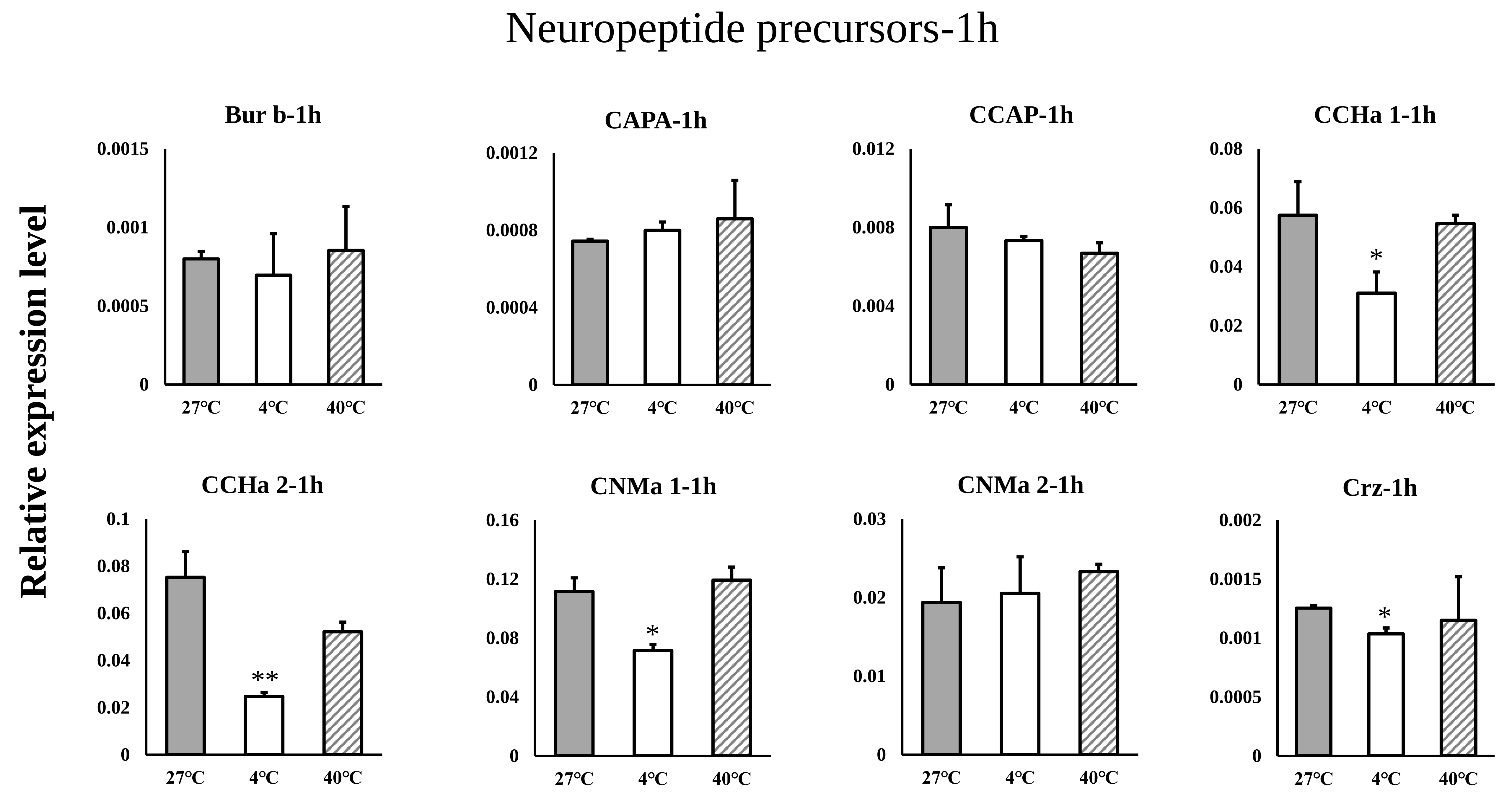
Neuropeptide precursors-1h
Relative expression level
Bur b-1h
0.0015
0.001
0.0005
0
CAPA-1h
0.0012
0.0008
0.0004
0
CCAP-1h
0.012
0.008
0.004
0
CCHa 1-1h
0.08
0.06
0.04
0.02
0
*
CCHa 2-1h
0.1
0.08
0.06
0.04
0.02
0
**
CNMa 1-1h
0.16
0.12
0.08
0.04
0
*
CNMa 2-1h
0.03
0.02
0.01
0
Crz-1h
0.002
0.0015
0.001
0.0005
0
*
27°C
4°C
40°C

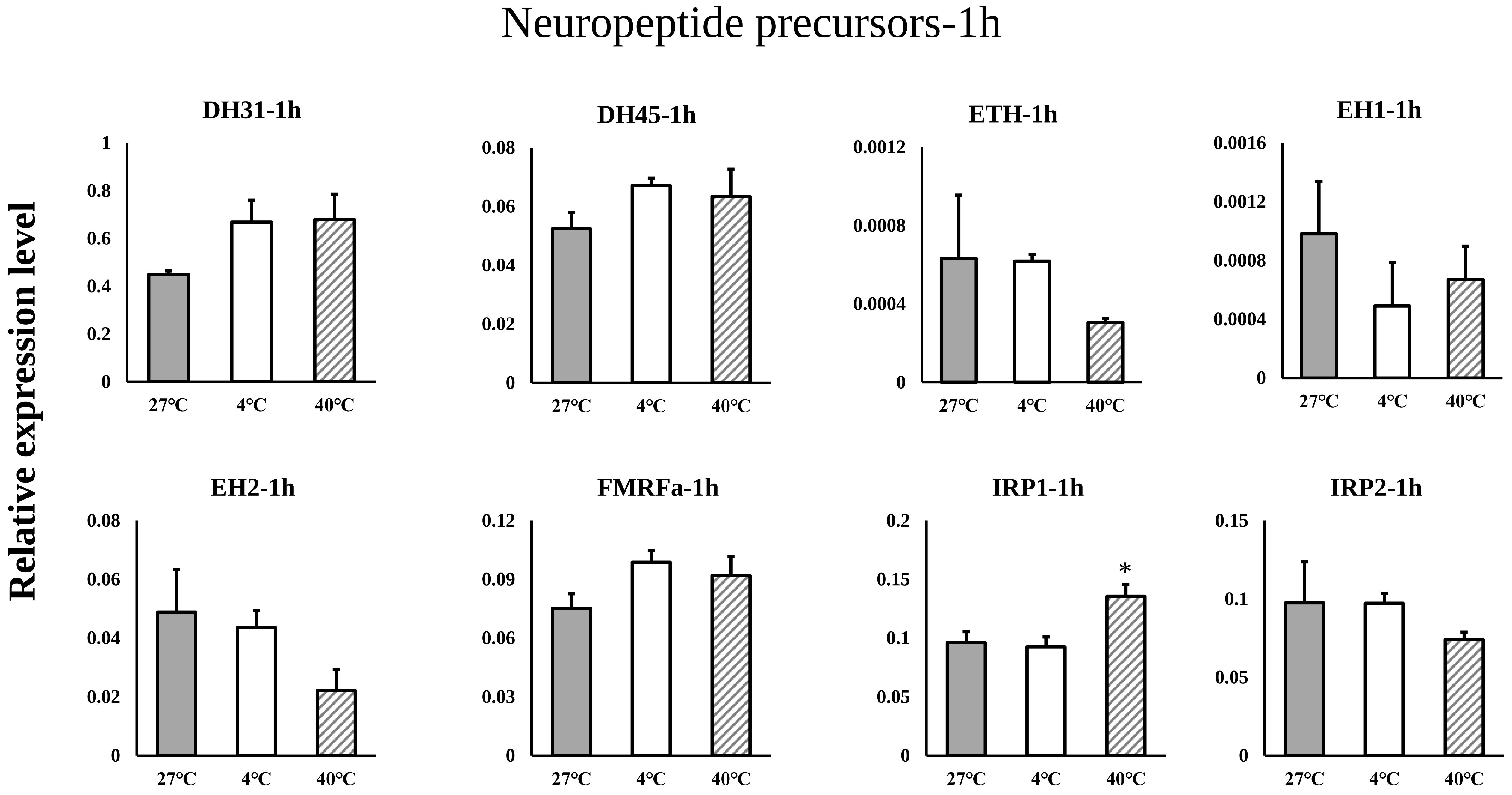
Neuropeptide precursors-1h
Relative expression level
DH31-1h
DH45-1h
ETH-1h
EH1-1h
EH2-1h
FMRFa-1h
IRP1-1h
IRP2-1h
1
0.8
0.6
0.4
0.2
0
0.08
0.06
0.04
0.02
0
0.0012
0.0008
0.0004
0
0.0016
0.0012
0.0008
0.0004
0
0.08
0.06
0.04
0.02
0
0.12
0.09
0.06
0.03
0
0.2
0.15
0.1
0.05
0
0.15
0.1
0.05
0
*
27°C
4°C
40°C

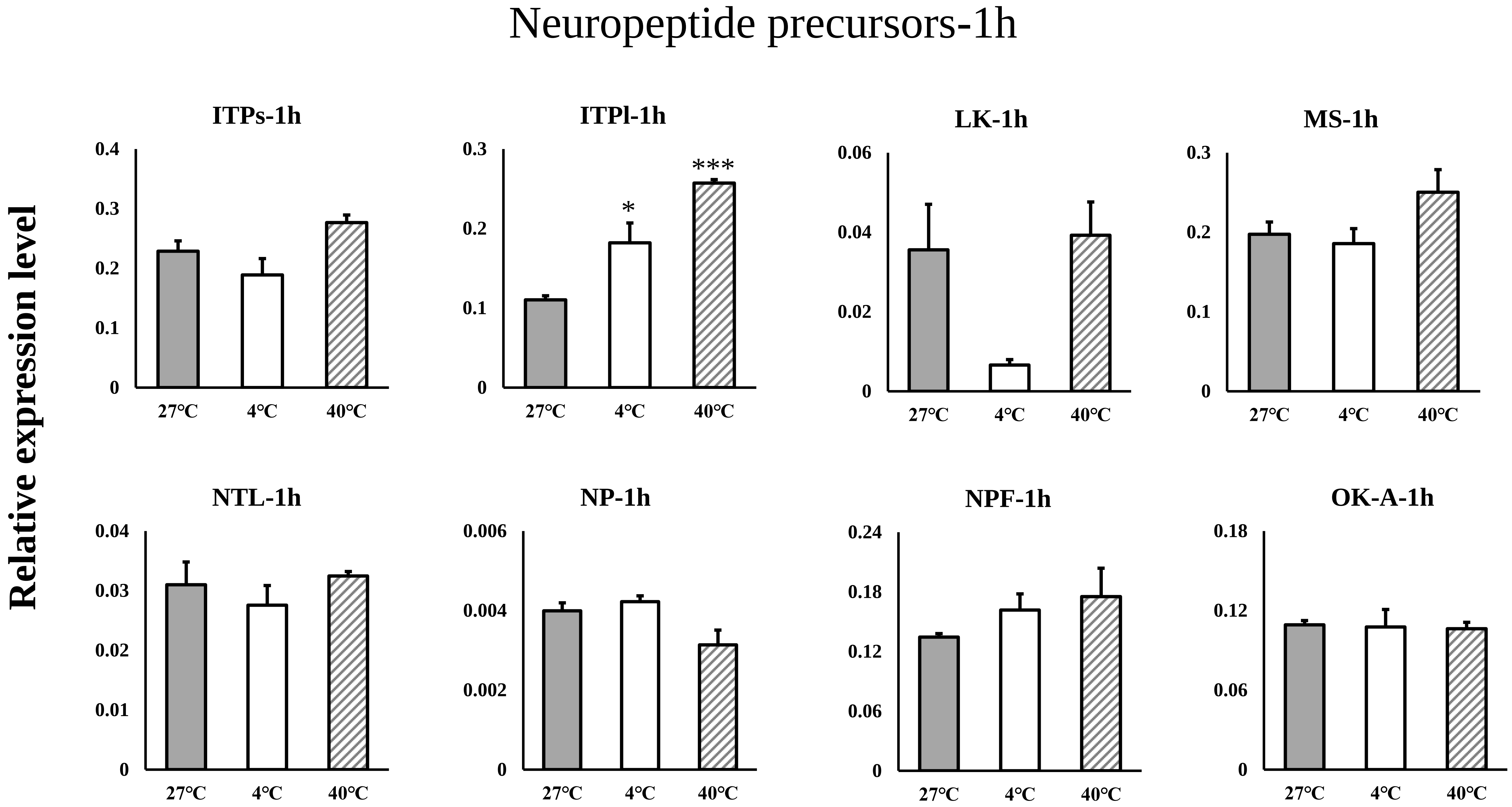

Neuropeptide precursors-1h
Relative expression level
ITPs-1h
ITPl-1h
LK-1h
MS-1h
NTL-1h
NP-1h
NPF-1h
OK-A-1h
27℃
4℃
40℃
*
***

Neuropeptide precursors-1h

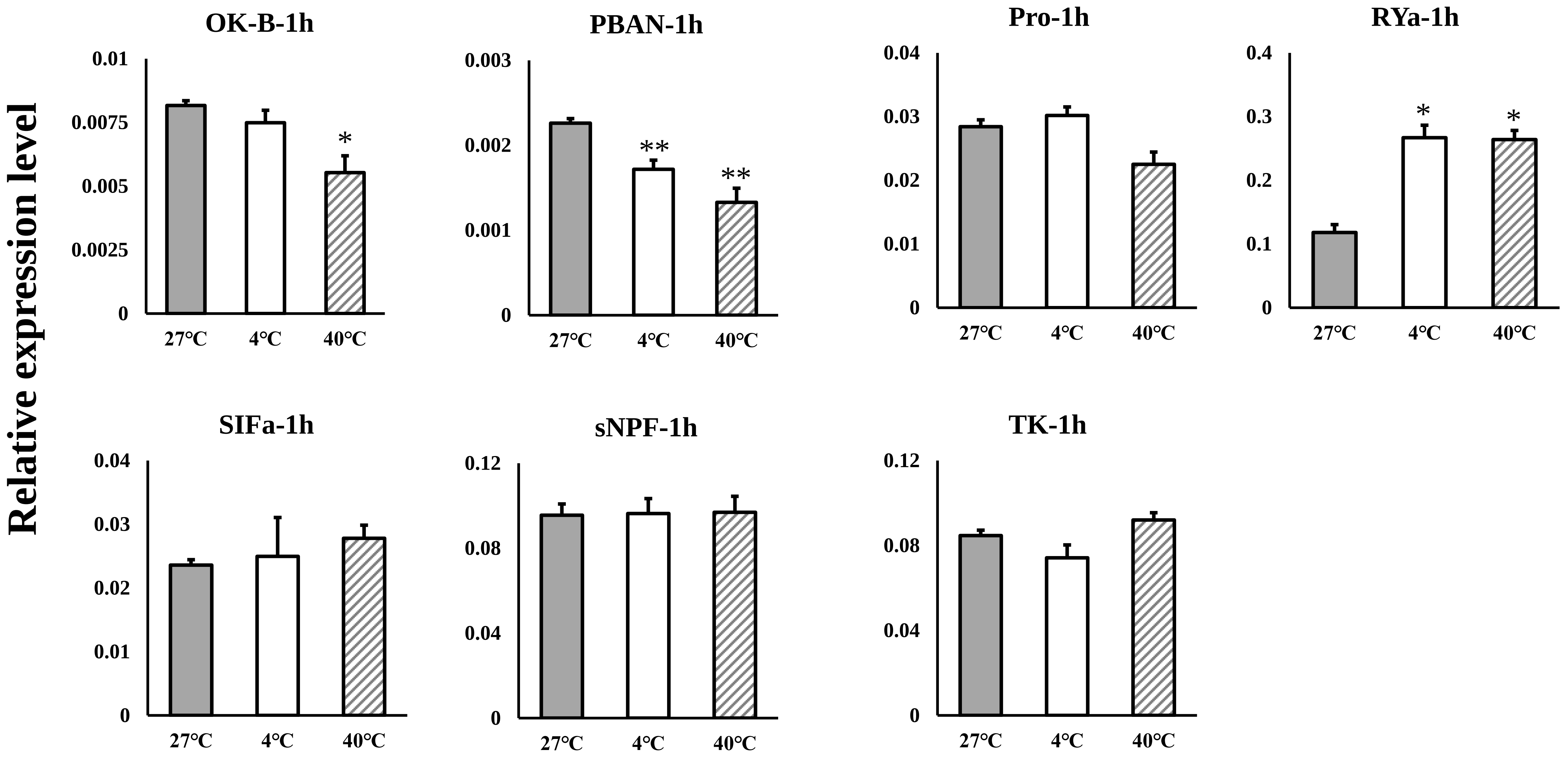

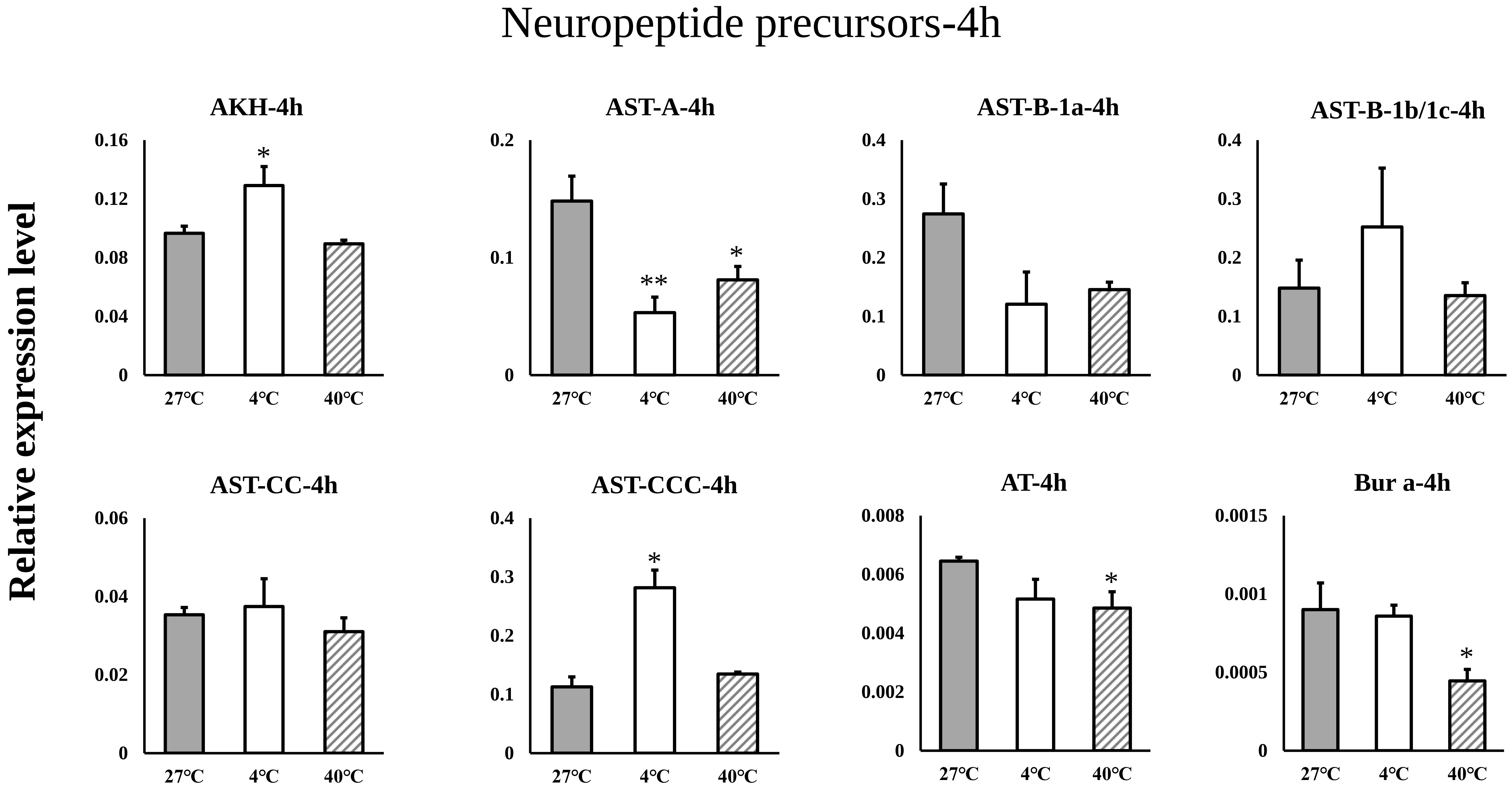

Neuropeptide precursors-4h
Relative expression level
AKH-4h
AST-A-4h
AST-B-1a-4h
AST-B-1b/1c-4h
AST-CC-4h
AST-CCC-4h
AT-4h
Bur a-4h
0
0.04
0.08
0.12
0.16
0.1
0.2
0.3
0.4
0.02
0.04
0.06
0.002
0.004
0.006
0.008
0.0005
0.001
0.0015
*
**
27°C
4°C
40°C

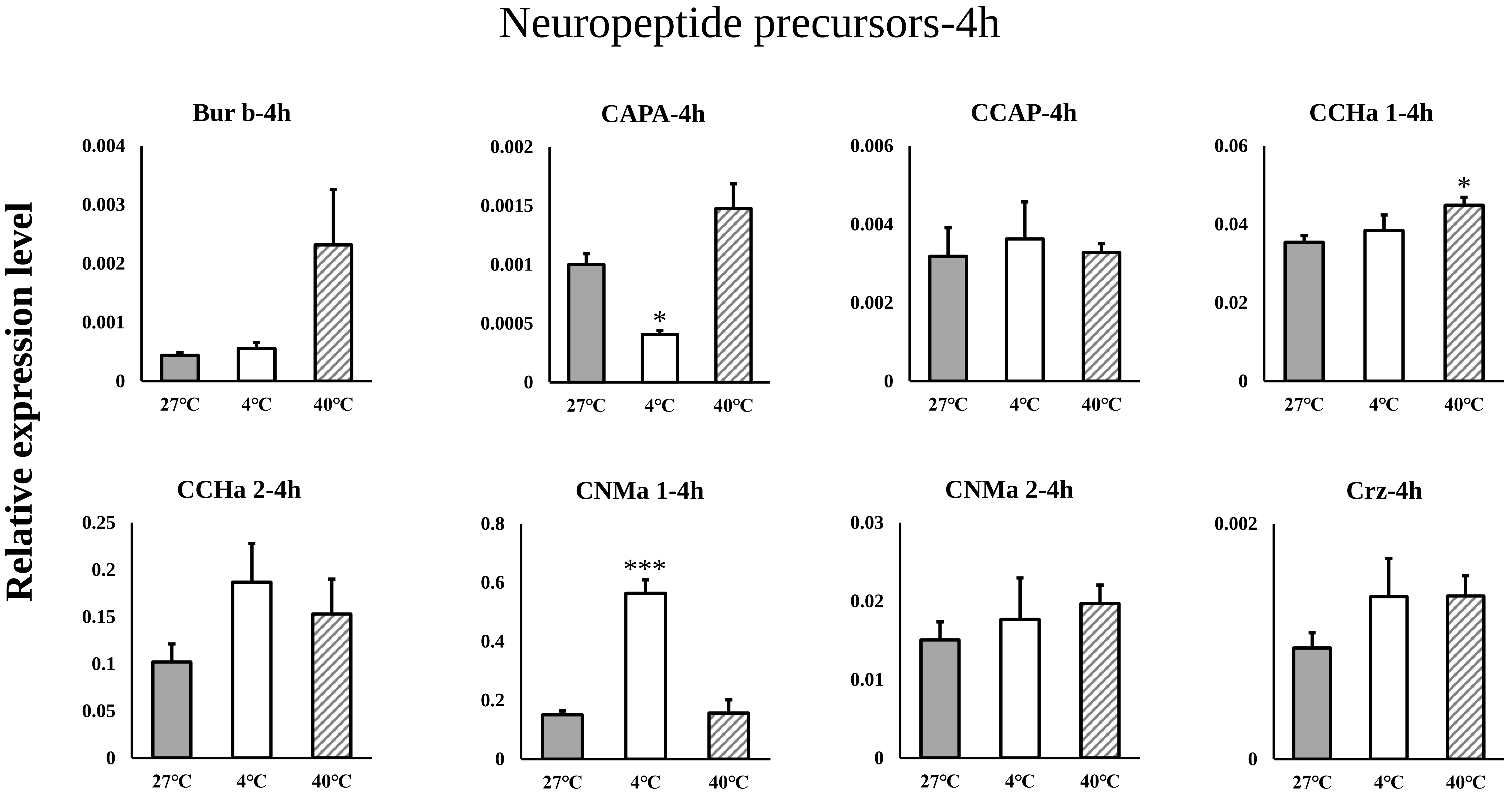
Neuropeptide precursors-4h
Relative expression level
Bur b-4h
CAPA-4h
CCAP-4h
CCHa 1-4h
CCHa 2-4h
CNMa 1-4h
CNMa 2-4h
Crz-4h
27℃
4℃
40℃
*
***

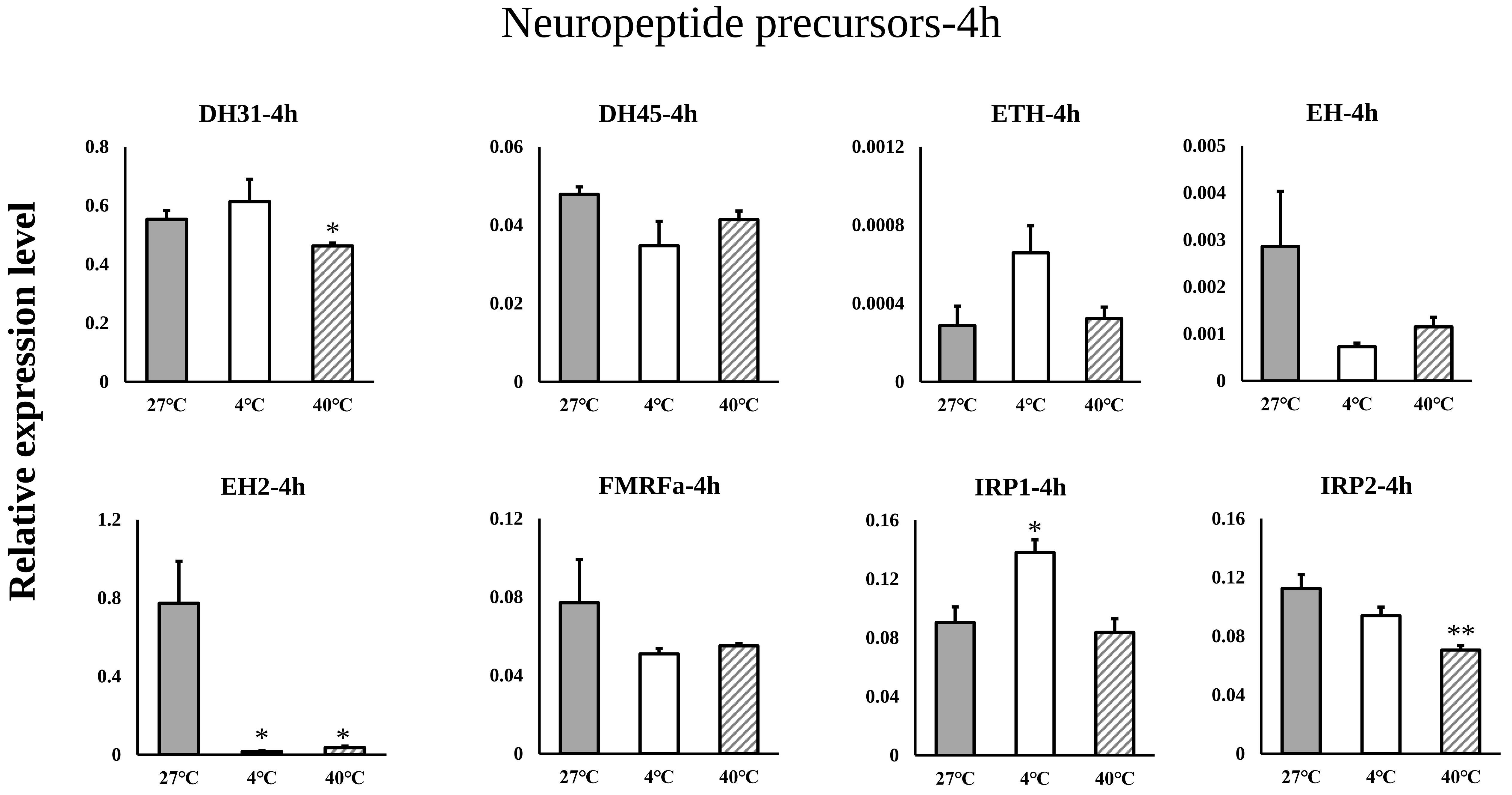
Neuropeptide precursors-4h
Relative expression level
DH31-4h
DH45-4h
ETH-4h
EH-4h
EH2-4h
FMRFa-4h
IRP1-4h
IRP2-4h
27℃
4℃
40℃
*
**

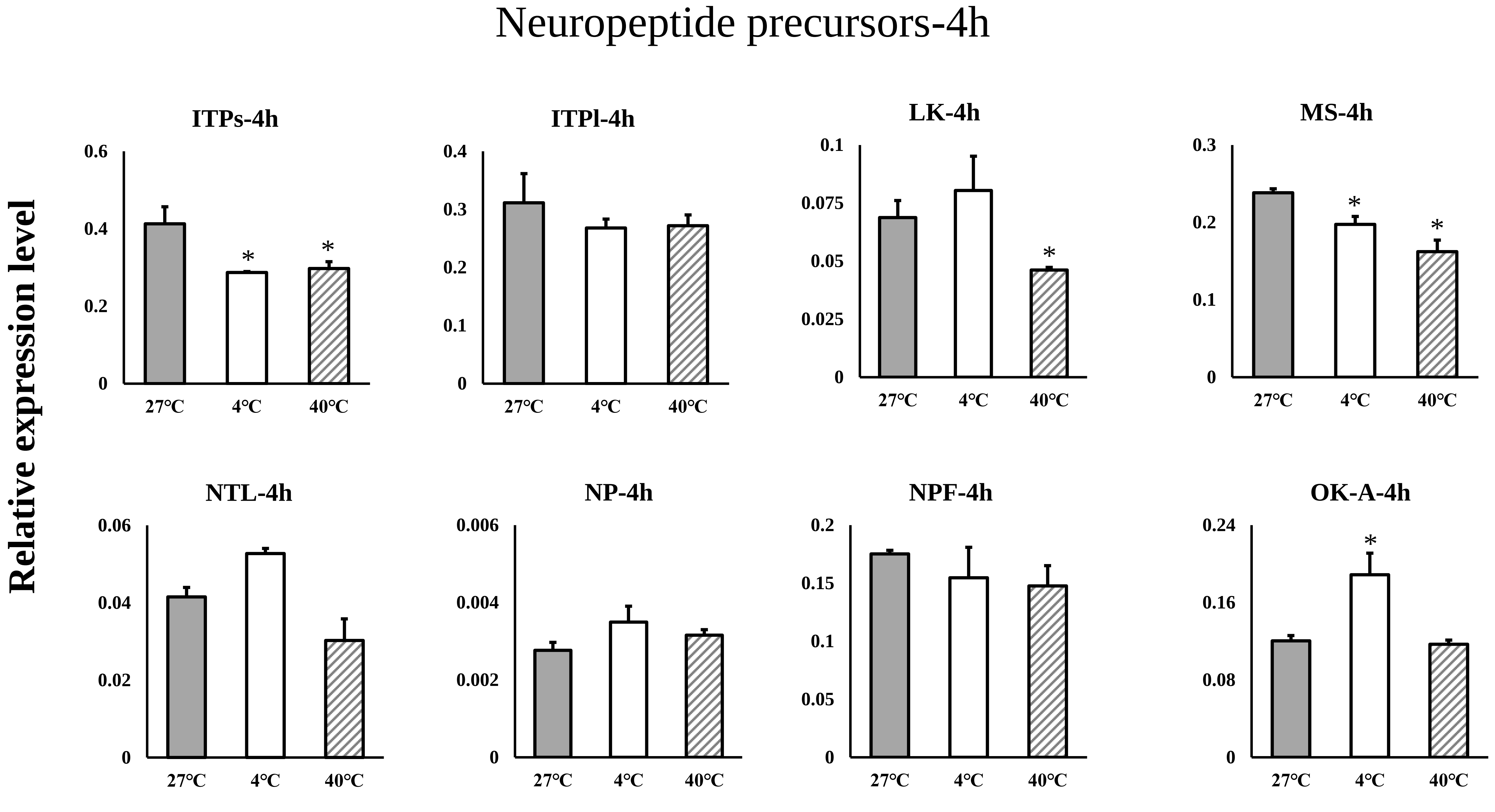
Neuropeptide precursors-4h
Relative expression level
ITPs-4h
ITPl-4h
LK-4h
MS-4h
NTL-4h
NP-4h
NPF-4h
OK-A-4h
27°C
4°C
40°C
*

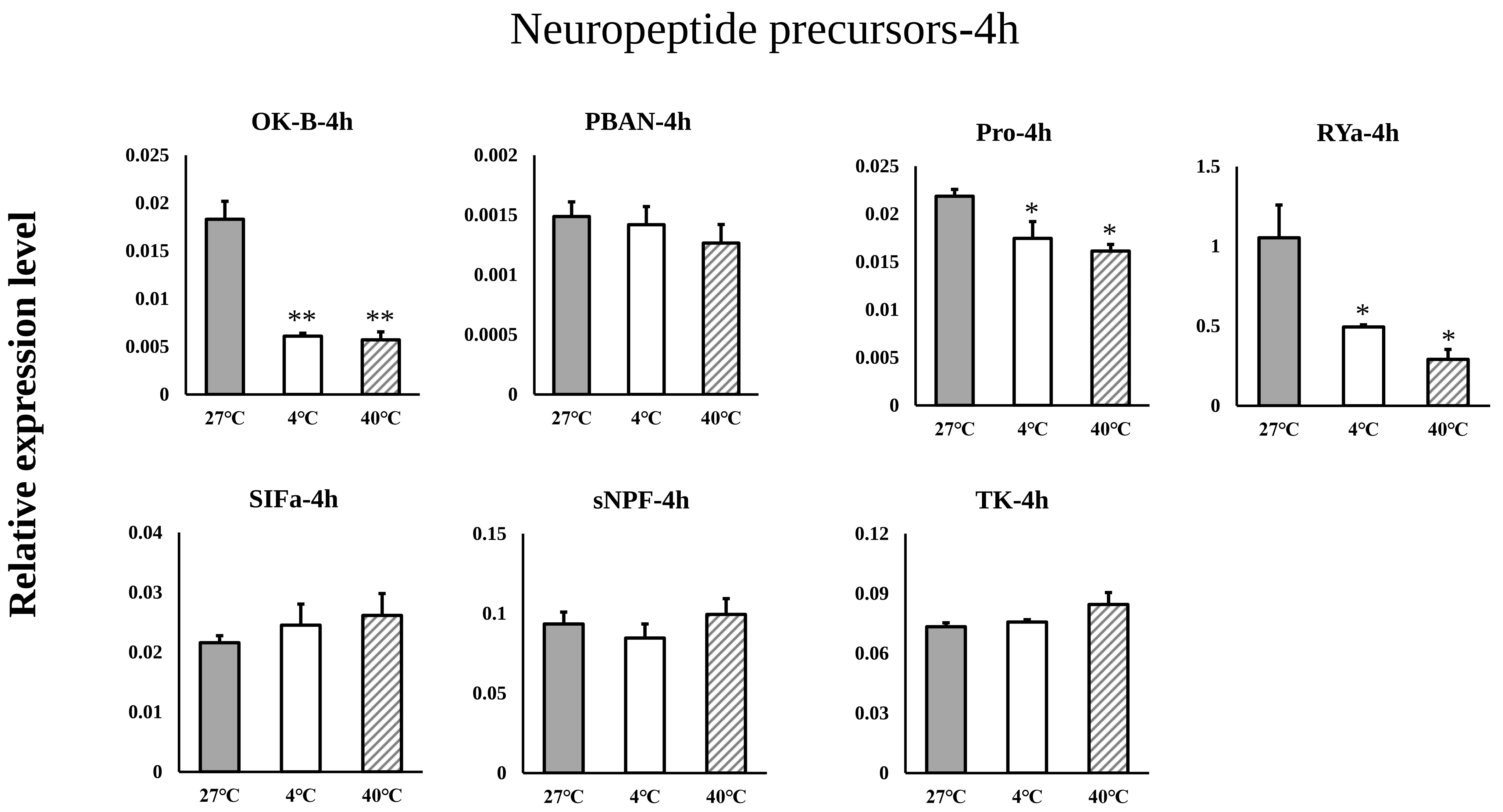
Neuropeptide precursors-4h
Relative expression level
OK-B-4h
0.025
0.02
0.015
0.01
0.005
0
**
**
27°C
4°C
40°C
PBAN-4h
0.002
0.0015
0.001
0.0005
0
27°C
4°C
40°C
Pro-4h
0.025
0.02
0.015
0.01
0.005
0
*
*
27°C
4°C
40°C
RYa-4h
1.5
1
0.5
0
*
*
27°C
4°C
40°C
SIFa-4h
0.04
0.03
0.02
0.01
0
27°C
4°C
40°C
sNPF-4h
0.15
0.1
0.05
0
27°C
4°C
40°C
TK-4h
0.12
0.09
0.06
0.03
0
27°C
4°C
40°C

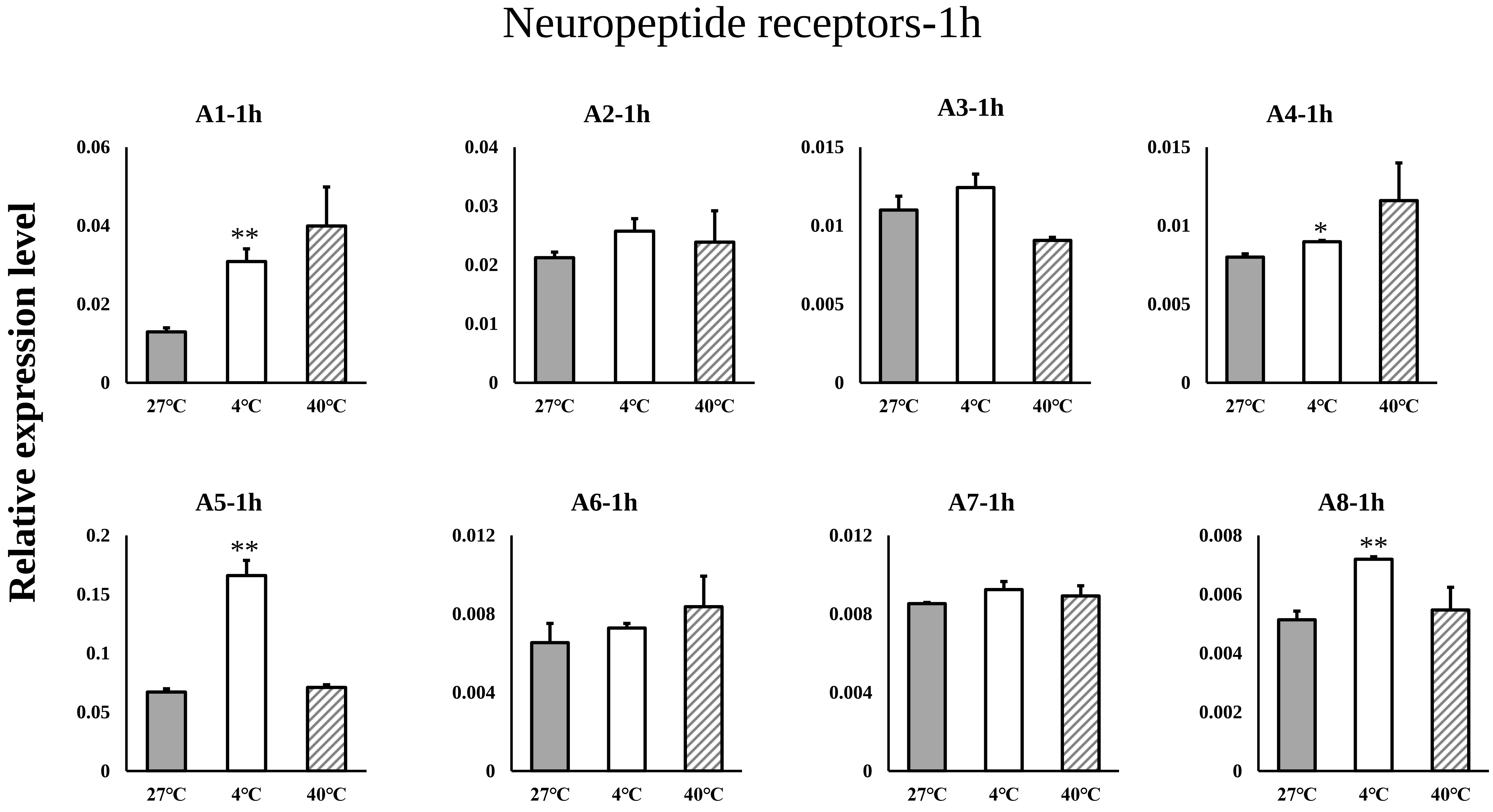
Neuropeptide receptors-1h
Relative expression level
A1-1h
0.06
0.04
0.02
0
**
27°C
4°C
40°C
A2-1h
0.04
0.03
0.02
0.01
0
27°C
4°C
40°C
A3-1h
0.015
0.01
0.005
0
27°C
4°C
40°C
A4-1h
0.015
0.01
0.005
0
*
27°C
4°C
40°C
A5-1h
0.2
0.15
0.1
0.05
0
**
27°C
4°C
40°C
A6-1h
0.012
0.008
0.004
0
27°C
4°C
40°C
A7-1h
0.012
0.008
0.004
0
27°C
4°C
40°C
A8-1h
0.008
0.006
0.004
0.002
0
**
27°C
4°C
40°C

Neuropeptide receptors-1h

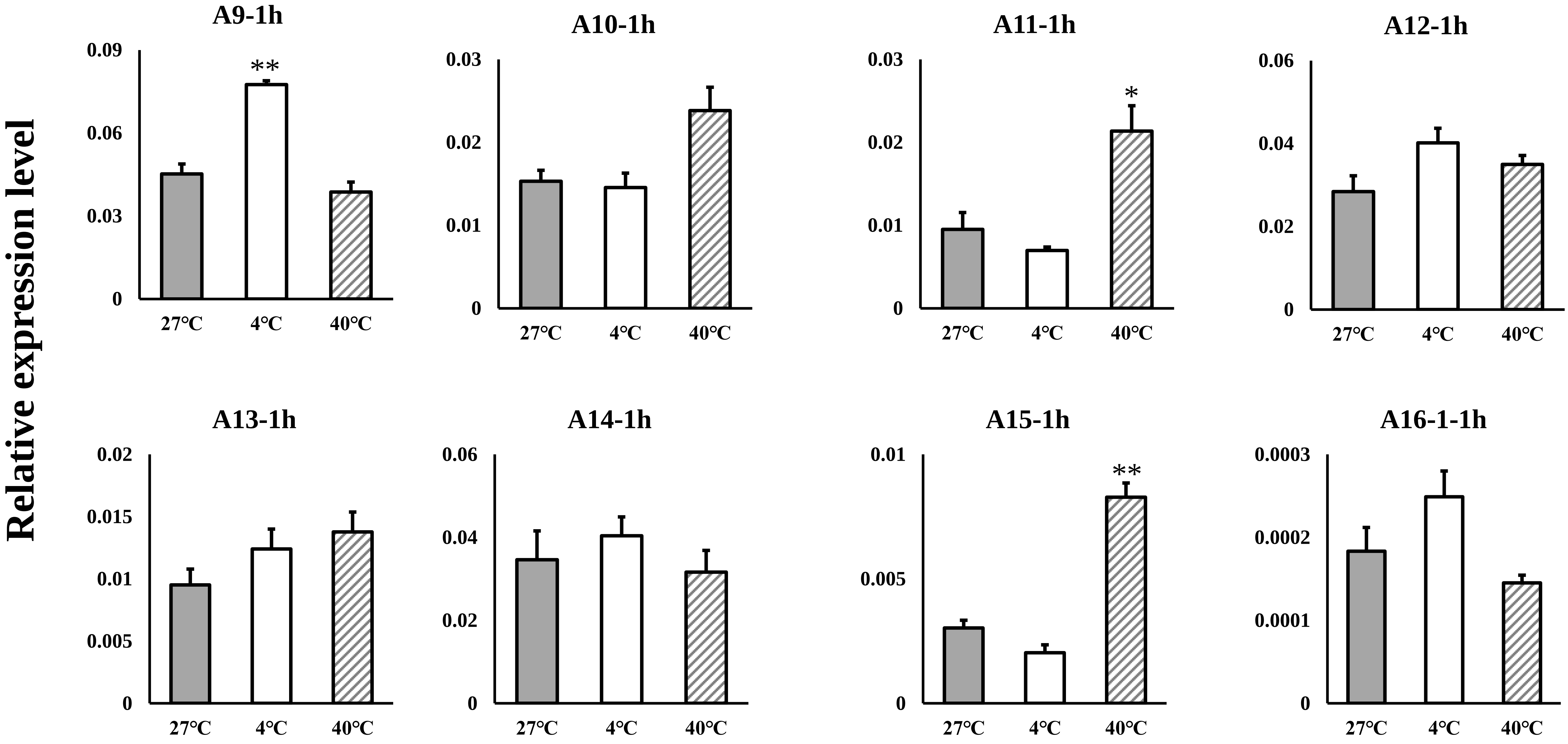

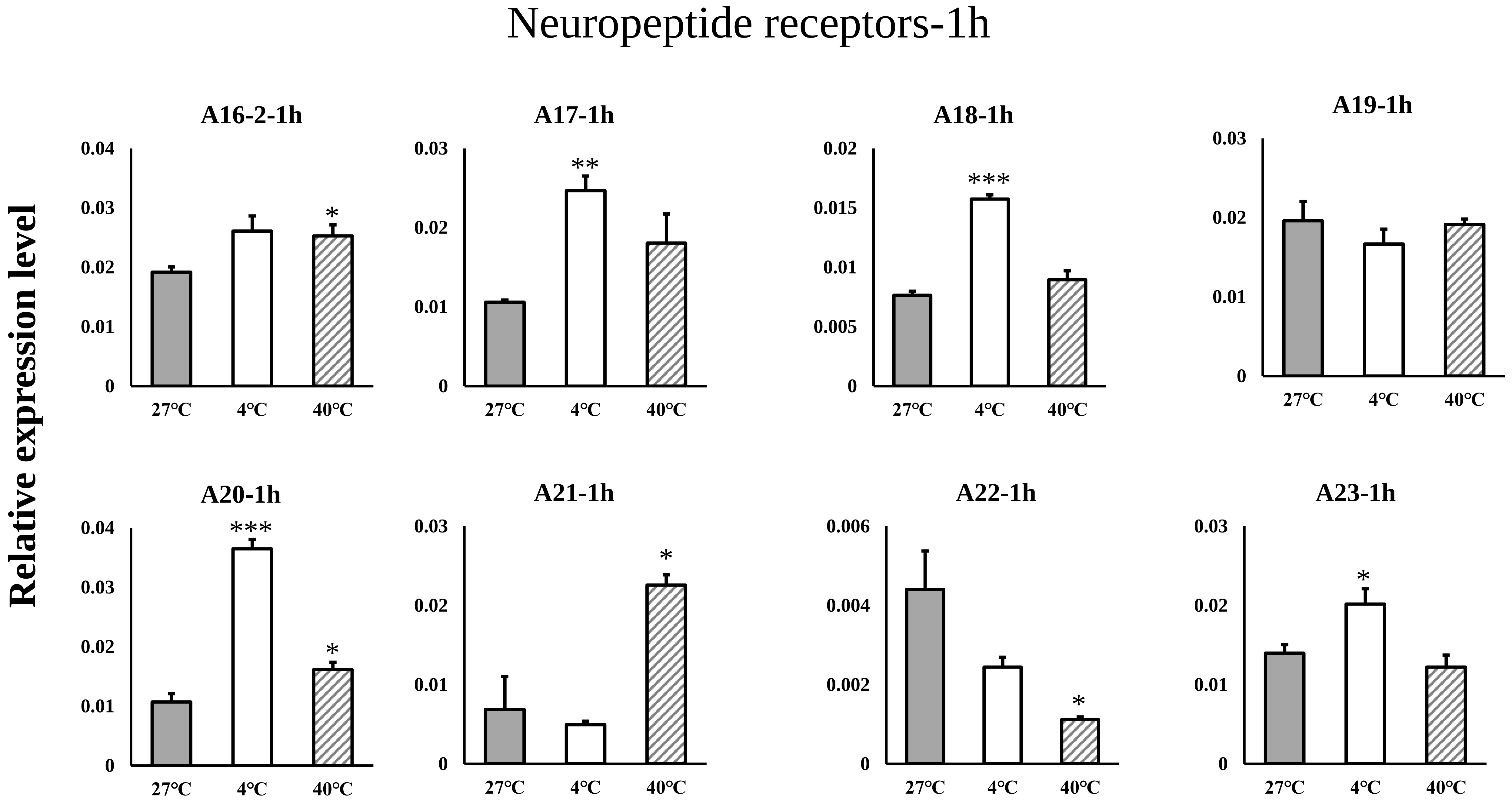

Neuropeptide receptors-1h
Relative expression level
A16-2-1h
A17-1h
A18-1h
A19-1h
A20-1h
A21-1h
A22-1h
A23-1h
27℃
4℃
40℃

Neuropeptide receptors-1h

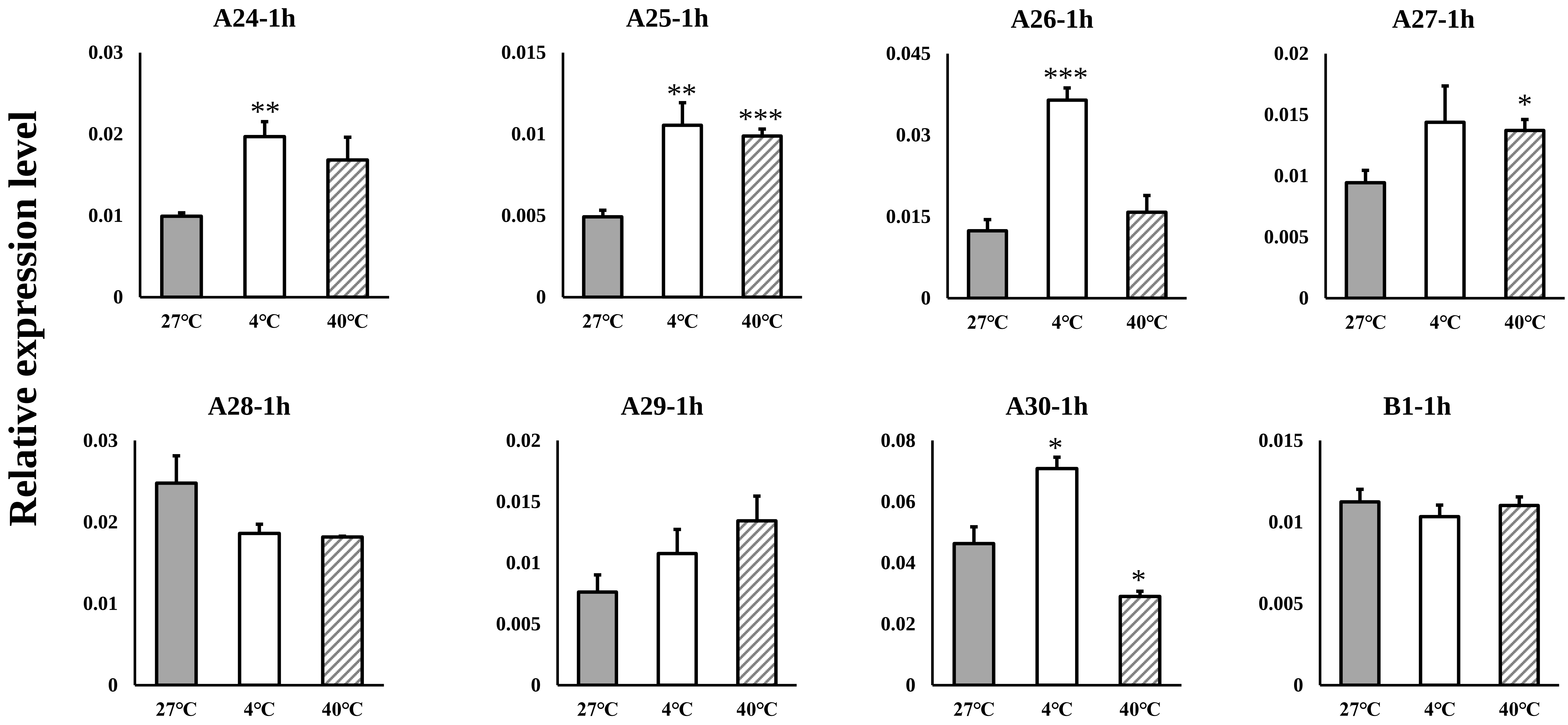

Neuropeptide receptors-1h

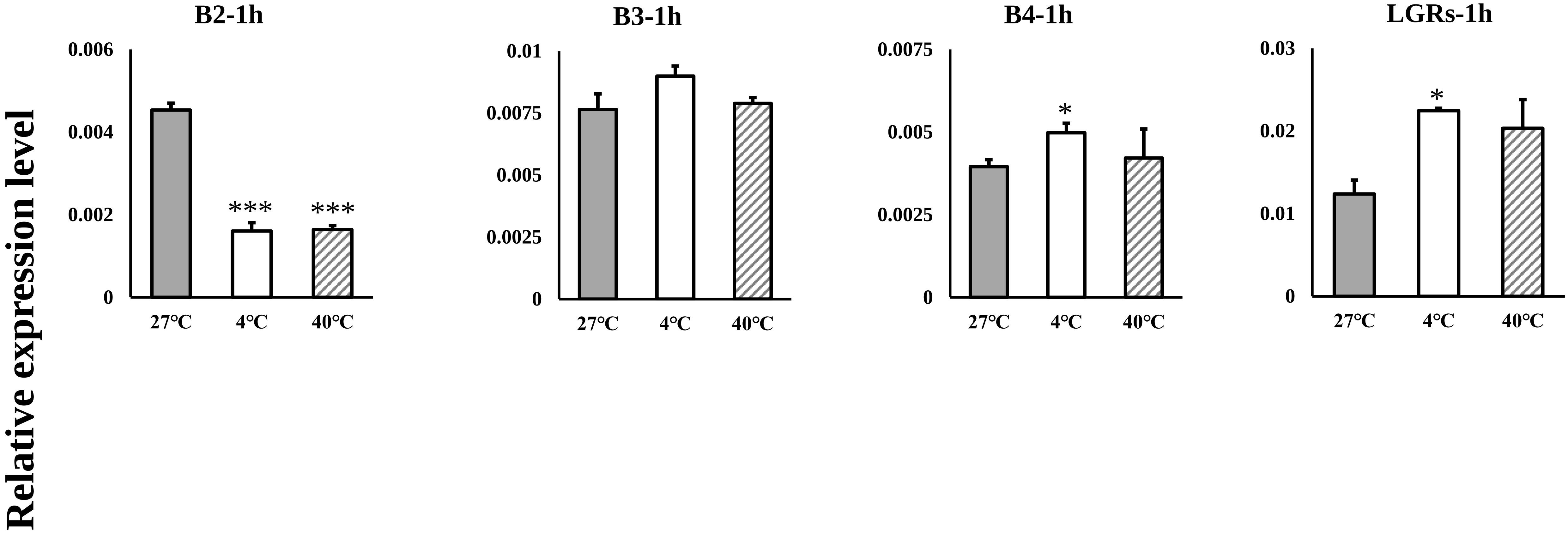

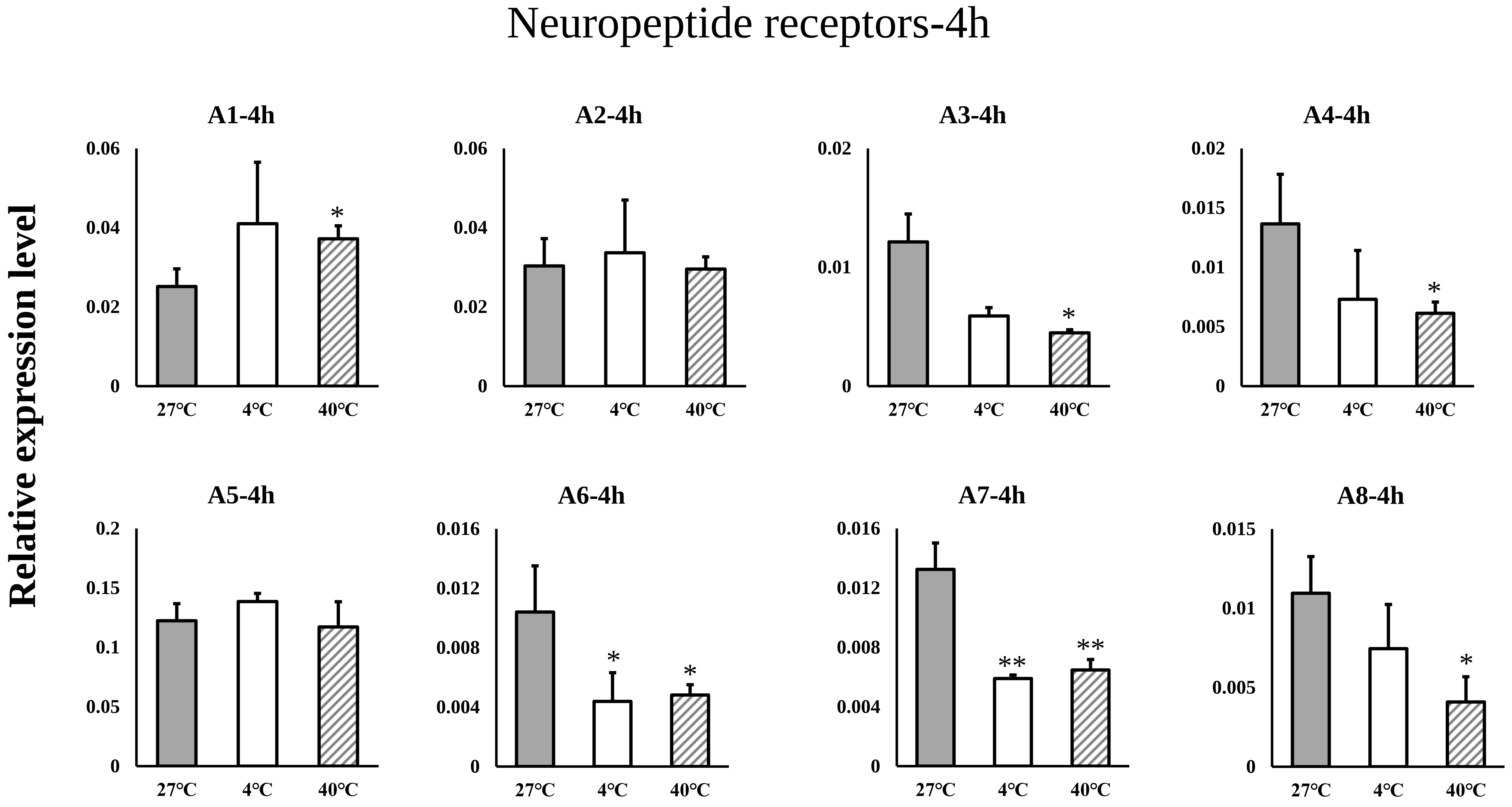
Neuropeptide receptors-4h
Relative expression level
A1-4h
A2-4h
A3-4h
A4-4h
A5-4h
A6-4h
A7-4h
A8-4h
27℃
4℃
40℃
0
0.02
0.04
0.06
0.01
0.005
0.015
0.05
0.1
0.15
0.2
0.004
0.008
0.012
0.016
*
**

Neuropeptide receptors-4h

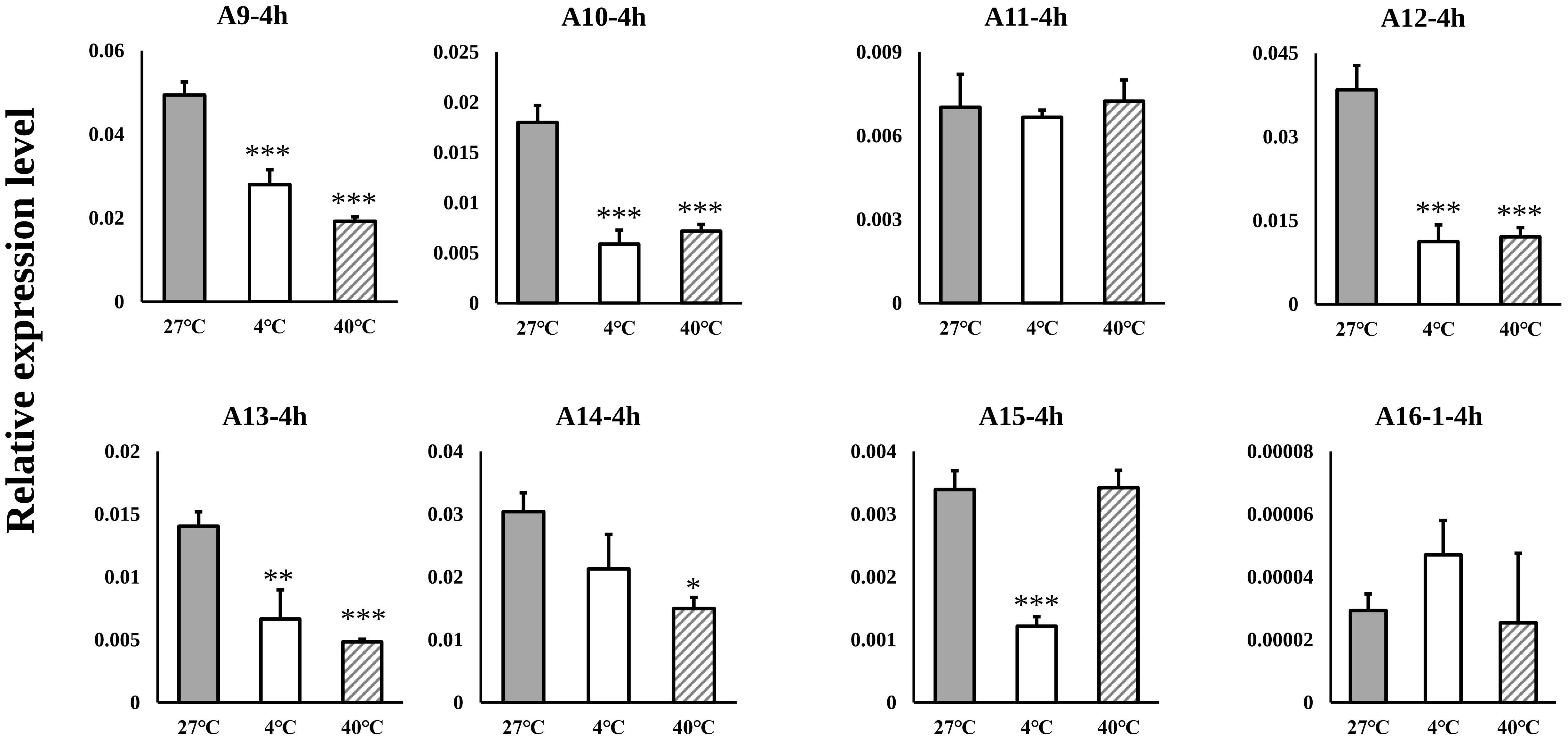

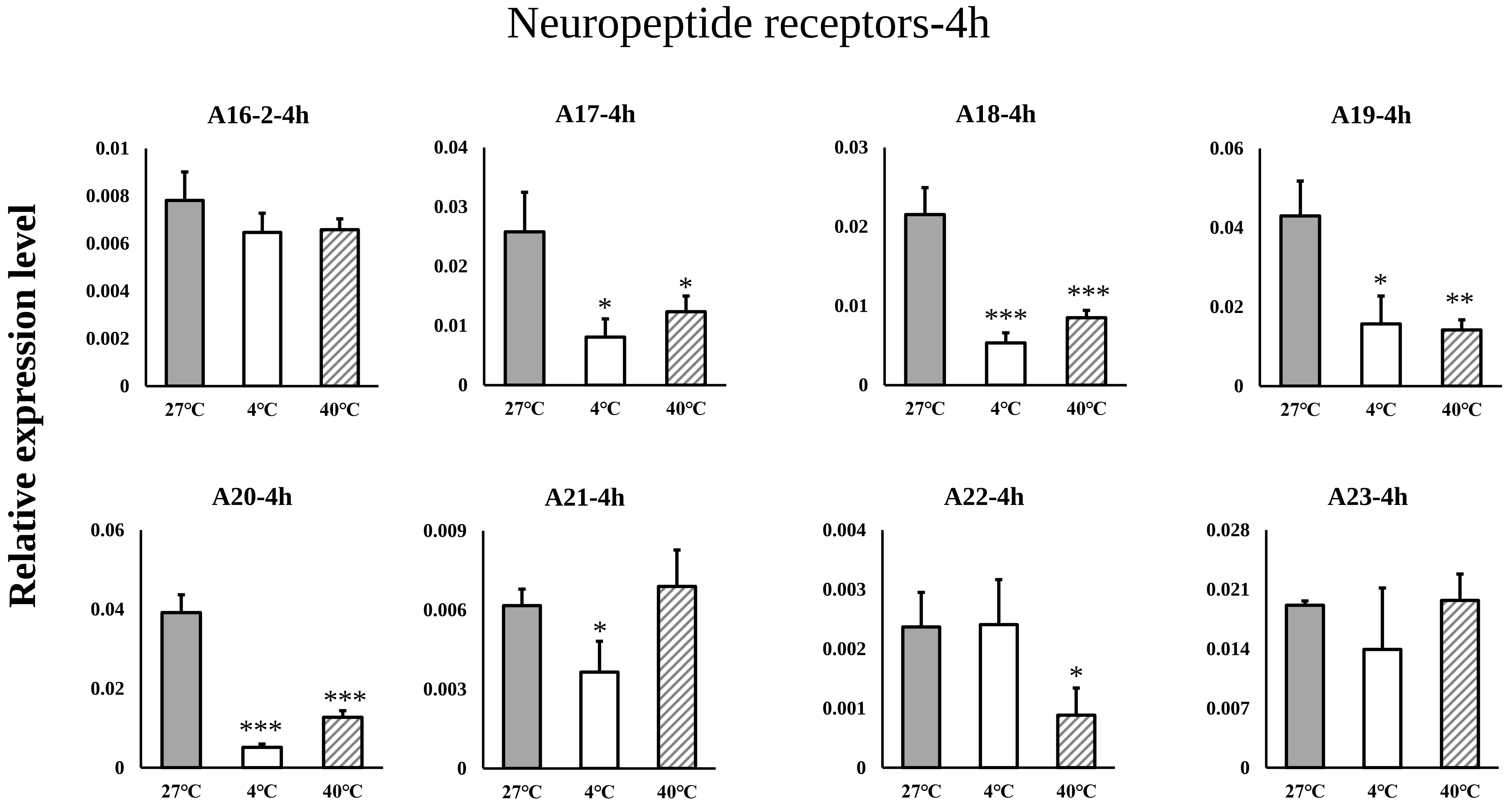
Neuropeptide receptors-4h
Relative expression level
A16-2-4h
0.01
0.008
0.006
0.004
0.002
0
27°C
4°C
40°C
A17-4h
0.04
0.03
0.02
0.01
0
*
*
27°C
4°C
40°C
A18-4h
0.03
0.02
0.01
0
***
***
27°C
4°C
40°C
A19-4h
0.06
0.04
0.02
0
*
**
27°C
4°C
40°C
A20-4h
0.06
0.04
0.02
0
***
***
27°C
4°C
40°C
A21-4h
0.009
0.006
0.003
0
*
27°C
4°C
40°C
A22-4h
0.004
0.003
0.002
0.001
0
*
27°C
4°C
40°C
A23-4h
0.028
0.021
0.014
0.007
0
27°C
4°C
40°C

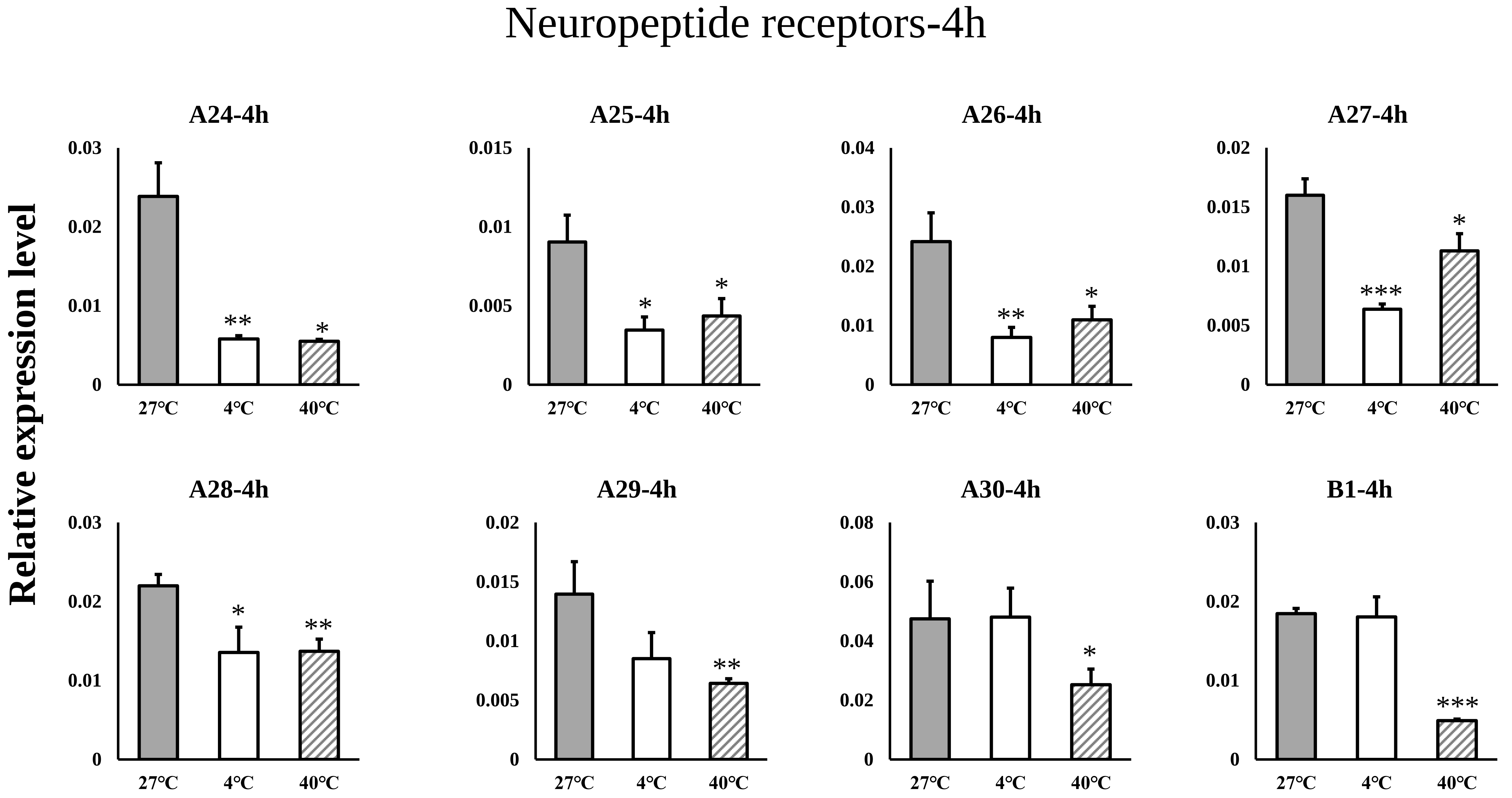
Neuropeptide receptors-4h
Relative expression level
A24-4h
A25-4h
A26-4h
A27-4h
A28-4h
A29-4h
A30-4h
B1-4h
0.03
0.02
0.01
0
0.015
0.01
0.005
0
0.04
0.03
0.02
0.01
0
0.02
0.015
0.01
0.005
0
0.08
0.06
0.04
0.02
0
27℃
4℃
40℃
*
**
***

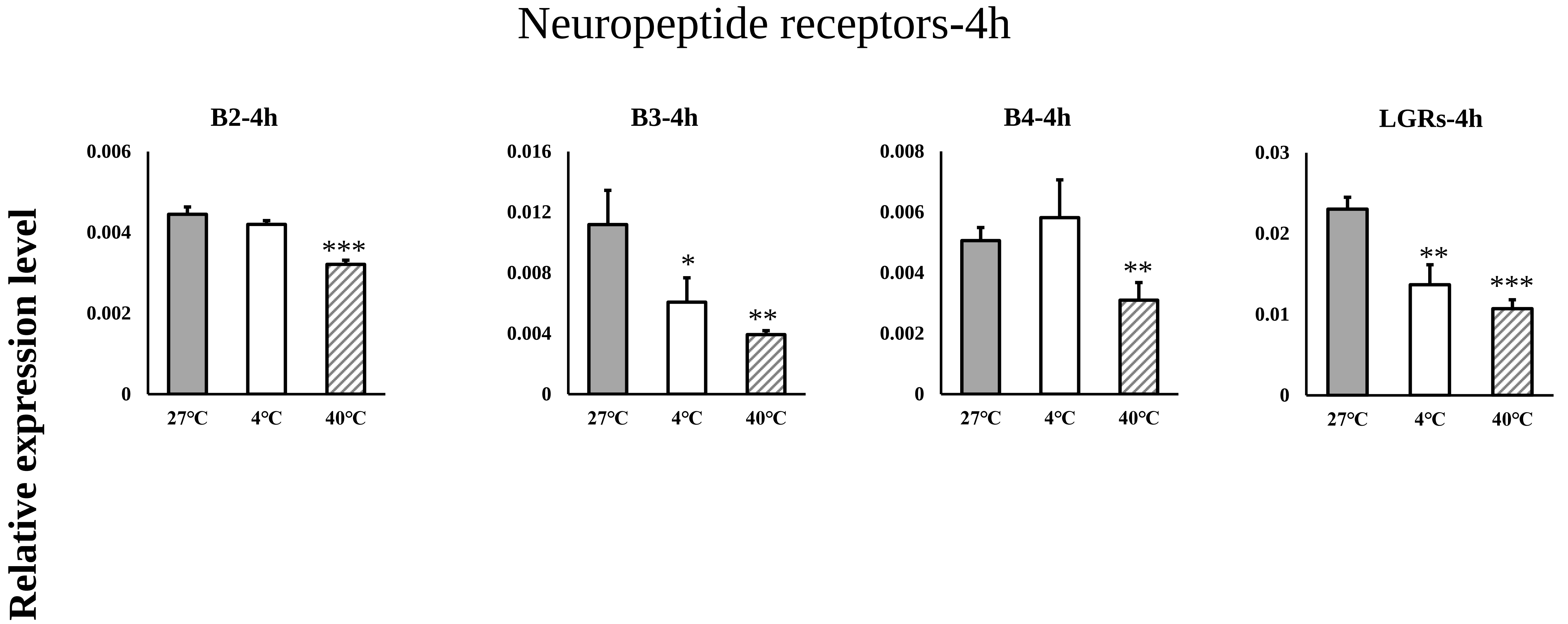
Neuropeptide receptors-4h
Relative expression level
B2-4h
0.006
0.004
0.002
0
***
27°C
4°C
40°C
B3-4h
0.016
0.012
0.008
0.004
0
*
**
27°C
4°C
40°C
B4-4h
0.008
0.006
0.004
0.002
0
**
27°C
4°C
40°C
LGRs-4h
0.03
0.02
0.01
0
**
***
27°C
4°C
40°C
