# Supplementary material for: Genome‐wide identification of neuropeptides and their receptor genes in Bemisia tabaci and their transcript accumulation change in response to temperature stresses
Source: Insect Sci. 2020 May 25;28(1):35–46. doi: 10.1111/1744-7917.12751 (PMC7818427; doi:10.1111/1744-7917.12751)
Supplement: Supplementary file 5 — Table S1 Neuropeptide G‐protein‐coupled receptor (GPCR) genes putative identified from Bemisia tabaci comparison with Diaphorina citri, Drosophila melanogaster and Zootermopsis nevadensis. [file INS-28-35-s005.pdf]

Table S1.
Neuropeptide G-protein-coupled receptor (GPCR) genes putative identified from *B. tabaci* Comparison with *D. citri*, *N. lugens*, *D. melanogaster* and *Z. nevadensis*. ND, not found; AS, absent in NCBI but published in the paper; *, incomplete or incorrect at NCBI in *Z. nevadensis*.

Receptor Name	*B.tabaci*	TMD (No.)	*D. citri*	*D. melanogaster*	*Z. nevadensis*	Putative Identification
Neuropeptide receptor A1	XP_018907642.1	7	MG550190	CG7285 CG13702	KDR19252*	Allatostatin-C receptor
Neuropeptide receptor A2	XP_018904604.1	7	MG550191	CG10001 CG2872	KDR11665*	Allatostatin-A receptor
Neuropeptide receptor A3	XP_018916865.1	7	MG550223	CG6515	KDR13925*	Natalisin receptor
Neuropeptide receptor A4	XP_018915862.1	6	MG550193	CG10823	KDR24477*	SIFamide receptor
Neuropeptide receptor A5	XP_018916522.1	7	MG550221 MG550222	CG11325	KDR09300	AKH receptor
	XP_018916523.1	7				
Neuropeptide receptor A6	XP_018915580.1	7	MG550195	CG5911	KDR21336* AS	ETH receptor
Neuropeptide receptor A7	XP_018896630.1	7	MG550196	CG7395	KDR21762 *	sNPF receptor
	XP_018896634.1	7				
Neuropeptide receptor A8	XP_018910474.1	7	MG550197	ND	KDR16982*	G-protein coupled receptor moody
Neuropeptide receptor A9	XP_018899885.1	7	MG550198	ND	KDR09651*	G-protein coupled receptor moody isoform X1
Neuropeptide receptor A10	XP_018916635.1	7	MG550199	CG16752	ND	Sex peptide receptor
Neuropeptide receptor A11	XP_018902557.1	7	MG550200	ND	ND	thyrotropin-releasing hormone receptor-like
Neuropeptide receptor A12	XP_018910014.1	4	MG550201	ND	AS	neuropeptide Y receptor type 1-like
Neuropeptide receptor A13	XP_018902188.1	7	MG550202	CG13803 CG8985	KDR23127	Myosuppressin receptor
Neuropeptide receptor A14	XP_018898689.1	7	MG550203	CG30106	KDR14578* AS	CCHamide-2 receptor
Neuropeptide receptor A15	XP_018898508.1	7	MG550204	CG14593		CCHamide-1 receptor
Neuropeptide receptor A16	XP_018905595.1	7	MG550205	ND	KDR20270* KDR20271*	Allatotropin receptor
	XP_018905596.1	7				

Neuropeptide receptor A17	XP_018909507.1	6	MG550206	ND		
Neuropeptide receptor A18	XP_018901010.1	7	MG550208	CG16726	KDR16422*	CNMamide receptor
	XP_018901008.1		MG550207			
Neuropeptide receptor A19	XP_018913547.1	7	ND	CG2114	KDR23532	FMRFamide receptor
Neuropeptide receptor A20	XP_018911170.1	7	MG550209	CG5811	KDR17076* KDR17079*	RYamide receptor
Neuropeptide receptor A21	XP_018916041.1	7	MG550210	CG10698	KDR13524*	Corazonin receptor
Neuropeptide receptor A22	XP_018911137.1	7	MG550211	ND	AS	RYamide receptor-like receptor
	XP_018911135.1	7				
	XP_018911138.1	6				
Neuropeptide receptor A23	XP_018897331.1	7	MG550212	CG10626	KDR12839*	Leucoinin receptor
Neuropeptide receptor A24	XP_018903913.1	7	MG550213	CG7887	KDR21658*	Tachykinin receptor
	XP_018903912.1	7				
Neuropeptide receptor A25	XP_018899674.1	7	MG550214	CG14575	KDR15327*	CAPA receptor
Neuropeptide receptor A26	XP_018915813.1	7	MG550215	ND	KDR19745	neuropeptide Y receptor type 2-like
Neuropeptide receptor A27	XP_018896491.1	7	MG550216	CG6111	KDR17833* KDR18368*	CCAP receptor
Neuropeptide receptor A28	XP_018900843.1	7	MG550217	CG1147	KDR21020*	NPF receptor
Neuropeptide receptor A29	XP_018916857.1	7	MG550218 MG550219 MG550220	CG9918	AS	Pyrokinin-1 receptor
Neuropeptide receptor A30	XP_018906643.1	7		CG8784 CG8795		Pyrokinin-2 receptor
Neuropeptide receptor B1	XP_018908584.1	7	MG550228	CG32843	KDR16174*	DH31 receptor
Neuropeptide receptor B2	XP_018912492.1	5	MG550229	CG13758	KDR09011*	PDF receptor
Neuropeptide receptor B3	XP_018897695.1	7	MG550230	ND	KDR11499*	Neuropeptide receptor B3
Neuropeptide receptor B4	XP_018910930.1	7	MG550231	CG12370 CG8422	KDR16173*	DH44 receptor
Leucine-rich repeat G-protein-coupled receptor 1	XP_018911955.1	6	MG550225 MG550226	CG7665	KDR15184* KDR06730*	Bursicon receptor
